# The effectiveness of a new parent education intervention program for primary school-age children and parents in Hong Kong

**DOI:** 10.1371/journal.pone.0331308

**Published:** 2025-09-15

**Authors:** Heidi Ka Ying Lo, Tommy Kwan Hin Fong, Calvin Pak Wing Cheng, Vivian Wai Yan Lui, Yiu Pan Wong, Phyllis Kwok Ling Chan

**Affiliations:** 1 Department of Psychiatry, The University of Hong Kong, Hong Kong SAR, China; 2 Department of Psychiatry, Queen Mary Hospital, Hong Kong SAR, China; Fondazione Policlinico Universitario Agostino Gemelli IRCCS, Universita' Cattolica del Sacro Cuore, ITALY

## Abstract

In Hong Kong, there is a recognised service gap and societal need for effective and evidence-based parenting education within mental health interventions. This study indtrocues the Parent Education in Mental Health (PEMH) intervention based on a theoretical framework. From 2021 to 2022, 2,246 parents from six elementary schools in Hong Kong participated in the PEMH program. An initial evaluation was conducted to asesses the effect of PEMH, with assessments at baseline and post-program. Parental stress was assessed by the Parental Stress Scale (PSS-18), parental self-efficacy was measured by the General Self-Efficacy Scale (GSE-10), the parenting style was evaluated by the Parenting Styles and Dimensions Questionnaire (PSDQ), and children’s mental health outcomes were measured using the Strengths and Difficulties Questionnaires (SDQ). Results showed that participation in the PEMH program led to significant improvements in parental stress, general self-efficacy, children’s mental health outcomes and shifts towards an authoritative parenting style. Focus groups conducted as part of the study highlighted the importance of social support in managing childcare stress and identified key components of the program for future enhancements. Parental stress was significantly associated with self-efficacy, parenting styles, and perception of children’s difficulties, while socioeconomic factors including higher educational level and income were associated with better parental and child outcomes. Our findings underscore the effect of PEMH in strengthening parental outcomes and child well-being, suggesting that a broader imputation of PEMH could be beneficial, particularly for parents of preschoolers.

## Introduction

### Background

Mental disorders among children and adolescents under 18 years old are a significant global concern [1], with an estimated prevalence of 13.4% (95 percent confidence interval [CI] = 11.3–15.9%) [2], with the prevalence exaggerated to 37.1% during the COVID-19 pandemic [3]. The common mental disorders in this age group include anxiety disorders, disruptive behavior disorders, Attention Deficit and Hyperactivity Disorder (ADHD), and depressive disorders [2]. The mental health scenario is particularly challenging in Hong Kong, where the prevalence of mental disorders in this age group was approximately 16.4%. The number of children and adolescents known to mental health services has more than doubled from 18,974 cases in 2011−12–43,589 cases in 2020−21 [2,4,5]. Compounding these challenges, Chinse parents with stronger traditional values often feel a sense of shame (‘ lose face’) towards their children’s mental health issues, which could be barriers to mental health services [6,7]. The limited resources of mental health services and stigma towards children’s mental health problems indicate an urgent need to strengthen early support and evidence-based approaches at the community level in Hong Kong [8]. Parenting has a pervasive impact on a child’s early development [9]. Educating parents about mental health can have wide-ranging benefits for children, from better cognitive and psychosocial development to protection from disease and mortality, resulting in better child health and development [10]. There is, therefore, a societal need for an effective parenting education program in mental health in Hong Kong.

In Hong Kong, several positive parenting programs, including Triple-P [11], Parent-Child Interaction Therapy (PCIT; [12]), and the 6As positive parenting program [13], have been conducted. However, these programs mainly focus on positive reinforcement, behavior training, and instilling positive principles of parenting through a universal-level approach [14]. These interventions do not cover both universal and indicated prevention involving preventive and remedial levels of interventions [15]. Moreover, these programs generally lack a theoretical framework that caters to the diverse needs of parents with children at different stages of development.

Parenting interventions have shown positive impacts on a range of parental and child outcomes [16]. Changes in processes of change in parent attitudes or perceptions of parenting styles have been directly observed [17]. Positive parental styles are associated with improved mental health outcomes in children [18]. Furthermore, enhancing parental self-efficacy, defined as parental confidence in using effective strategies and behaviors to influence child development and well-being, is another important aspect highlighted in parenting interventions [19,20]. Parental self-efficacy is positively correlated with positive parenting strategies, coping strategies, endurance in handling demanding situations, and socio-emotional, behavioral, and academic skills of children [20]. Furthermore, parental stress, impaired emotion regulation, and low self-efficacy are parenting-related factors that can impact a child’s social and emotional well-being [21]. Family stress theory suggests that parental stress may disrupt family equilibrium, thereby contributing to children’s emotional and behavioral problems [22]. Accordingly, self-report measures from parents, including assessments of children’s mental health outcomes, general self-efficacy, parental stress, and parenting styles, provide a convenient [17] and ecologically valid assessment method [23], considered superior in criterion and predictive validity [24]. Incoroporating a qualitative evaluation through focus groups could help ascertain the acceptance and perceived usefulness of the components of these parenting programs [25 ].

Moreover, the association between socioeconomic status, as indicated by factors such as income-to-needs ratio, parental education, and employment status, and the outcomes of parental interventions, as suggested in Western literature [25–27], may vary due to cultural distinctions [27]. For instance, a previous parental education program in Hong Kong reported lower family income levels and higher baseline parental stress [11]. Alternatively, it also indicated a correlation between higher educational level [28] was correlated to better effectiveness of the program. These observations highlight the need for systematic assessment of the underlying parental psychological attributes (e.g., parental stress, parental self-efficacy, and parenting style), and socioeconomic factors (e.g., family income, or educational level) that are associated with intervention effectiveness.

### Parent education in mental health (PEMH) intervention

The newly developed Parent Education in Mental Health (PEMH) intervention aims to provide a comprehensive, multi-level approach to parenting education. PEMH aims to equip parents with the knowledge and skills necessary to identify and address their children’s mental health concerns, enhance relationships, and effectively manage their own stress. Grounded in evidence-based theories including social learning theory in developmental psychology [19], positive parenting [29], mindful parenting [30], and parental resilience research under Bronfenbrenner’s ecological system theory [31,32], this program is led by registered social workers who have undergone four sessions of professional training in parents’ education by child psychiatrists.

By combining these educational theories and psychological principles, the program aims to (1) enhance understanding of the emotional and social development of children and adolescents; (2) strengthen the emotional bonding between parent and child; (3) enhance positive parental involvement in their children’s education, behavior, and well-being; and (4) provide stress management skills to reduce parental stress. This comprehensive program is structured into three levels, catering to the diverse needs of parents, ranging from universal education talks and workshops to targeted small group workshops and individualized case management for complex cases. Level one is for all parents (universal program) including education talks and workshops. Level two is an intermediate-level program and is provided to those who are interested in further strengthing their parenting skills. The format would be mainly small group workshops focused on specific topics. Level three mainly deals with complex cases, adopting one to one case management approach. Detailed theme, content, and concept are outlined in Tables 1 and 2 [33,34].

**Table 1:** Scope and Sequence of Theme, Content and Concept of Level 1 of the PEMH program.

Unit	Theme	Content	Concept
1	Understanding and addressing the emotional and social development of children and adolescents	Covering key emotional and social developmental milestones, effective strategies for supporting children’s emotional growth, and fostering healthy social relationships.	Based on social learning theory in developmental psychology, which examines how individual child grow and change throughout their lives.
2	Recognizing and addressing early mental health issues in children and adolescents	Discussing common mental health issues in children and adolescents, signs and symptoms, and evidence-based approaches to support their mental well-being.	Grounded with principle of early intervention, which focuses on identifying and addressing mental health concerns as soon as possible.
3	Mindfully coping with stress – Mindfulness-Based Stress Reduction (MBSR)	Introducing the principles of MBSR, and ways to incorporate these practices into their daily lives to better manage parenting stress.	With the concept of mindfulness, which involves developing non-judgmental awareness of one’s thoughts, feelings, and experiences.
4	Acute stress response – Trauma emotional processing	How to manage the unpredictable stresses in daily life.	It examines enhancing emotional processing with trauma-informed care.
5	Enhancing children’s homework, self-care, and daily routines through executive function	How to improve executive function for a set of cognitive skills that help individuals plan, organize, and complete tasks.	It focuses on limit and potential of cognitive load and executive functioning of the child.
6	New Parenting Discipline Model: Combining Skills and Mindful Education	Introducing effective disciplinary strategies with mindfulness techniques, enabling parents to foster a supportive and nurturing environment for their children.	Based on the principles of positive discipline and mindful parenting, which emphasize communication, empathy, and problem-solving.
7	Supporting the Growth of Introverted, Uneasy and Insecure children	Covering strategies for identifying and cultivating their strengths,as well as fostering their self-confidence and resilience.	Based on the strengths-based approach, it focuses on developing and nurturing the unique strengths and abilities of each individual.
8	The Joys and Challenges of Parenting Adolescents: Equipping Parents	Understand the needs adolescents, and how to support their resilience building with support for transitioning to secondary school.	The adolescent age during transition to secondary school faces its own unique challenges and opportunities, focusing on the unique needs and resilience building.

**Table 2:** Scope and Sequence of Theme, Content and Concept of Level 2 of PEMH program.

Unit	Level 2 Workshops	Content	Concept
1	Primary 1: Children’s discipline and education training – Basic course (4 sessions)	Topics include effective communication, setting boundaries, fostering a growth mindset, and understanding age-appropriate expectations.	Understanding observable behavior and positive discipline, it emphasizes the importance of empathy and problem-solving.
2	Primary 2: Children’s discipline and education training – Extended course (3 sessions)	Building on the basic course, it delves deeper into advanced strategies for discipline and education, such as conflict resolution, emotional regulation, and effective praise.	Based on social learning theory and self-determination theory, it aims to foster intrinsic motivation.
3	Primary 1–2: Enjoyable homework (5 sessions)	Developing a positive attitude toward homework by incorporating engaging activities, time management techniques, and strategies.	With techniques of cognitive load and gamification, transforming learning to an engaging and enjoyable experience.
4	Primary 3–4: Happy children’s charter (4 sessions)	Developing social-emotional skills, including empathy, assertiveness, and emotional intelligence, through interactive activities and group discussions.	Grounded in emotional intelligence theory, which highlights the importance of recognizing, understanding, and managing emotions in oneself and others.
5	Primary 3–4: Healthy internet usage Basic workshop (2 sessions) and Advanced workshop (4 sessions)	Developing responsible and safe internet usage, including topics such as online privacy, cyberbullying, and managing screen time.	Based on media literacy education, it helps parents to acknowledge the influence of media on behavior.
6	Primary 5–6: Adolescent discipline and education training (3 sessions)	Covering discipline and education strategies specific to adolescent development, including topics such as autonomy, identity formation, and peer relationships.	It specifically focuses on the stage of identity vs. role confusion, as well as principles from attachment theory, and Erikson’s stages of psychosocial development,
7	Primary 5–6: “Growing up” – Learning to interact with the opposite sex (6 sessions)	Developing healthy relationships with the opposite sex, exploring topics such as communication, boundaries, respect, and understanding differences.	Development and maintenance of effective communication and relationships with interpersonal skills.
8	Primary 6: Transitioning to secondary school support (3 sessions)	Supporting transition, through time management, goal setting, stress management, and building social connections.	It focuses on helping students develop effective learning strategies and habits, and encouraging eliciting appropriate resources based on principles from self-regulated learning

### Present study

In the present study, we first examined the parents’ self-efficacy, parenting styles, stress levels, and the child mental health outcomes before and after their participation in the PEMH program. Second, we employed a feedback survey and focus group approach to evaluate parental stress and difficulties in childcare, the mental health of parents and children, and program feedback. This two-year initiative in Hong Kong involved collaborations from psychiatrists, non-governmental organisations (NGOs), and six local primary schools, operationalized with three goals: (1) enhance parenting skills, (2) alleviate parental stress, and (3) improve parent-child communication. The workshops were led by social workers from three local non-government organizations, who were thoroughly trained by the research team prior to their involvement. The social workers underwent a total of 30 hours of training across four distinct sessions. The first three sessions were dedicated to preparing their knowledge and skill sets for the program, while the fourth session, conducted mid-program, focused on evaluations and consolidating their knowledge. Our primary objective was to pilot the PEMH program and hypothesize its favourable effects on parental strategies, self-efficacy, well-being, and parental stress; and children’s mental health outcomes. We also anticipated that socio-economic factors (e.g., educational level, monthly household income, etc.) would correlate with the parental and children’s outcomes at post-intervention.

## Methods

### Study design and participants

#### Quantitative.

Parents from the dedicated local primary schools were invited to participate via the notice by the primary school. The participation of parents was on voluntary basis. Parents were recruited for the study in July 2020 and 2021 respectively. Follow-up data were collected at the end of each academic year (i.e., 2021 July and 2022 July). This study was approved by the Institutional Review Board of The University of Hong Kong/Hospital Authority, Hong Kong West Cluster (HKU/HA HKW IRB) (reference no.: UW 20–472). All participating parents completed the baseline and follow-up self-rated questionnaires in traditional Chinese via the Qualtrics Survey platform (https://www.qualtrics.com), with completion of questionnaires as part of the regular service delivery practice. Hard copies of questionnaires were provided for those participants who were not familiar with digital tools. A 2x2 quasi-experimental approach, a widely adopted experimental design in parent-child service evaluations, facilitated the assessment of the intervention’s effects. This design differentiated between two groups: an intervention group and a control group (those not participating in the Parental Engagement in Mental Health (PEMH) program). Additionally, it measured changes over two time points (pre-intervention and post-intervention), thereby acting as a within-subjects factor. To ensure confidentiality, all parents completed the survey anonymously, and no individual participant information was identifiable during or after data collection.

#### Qualitative.

*Focus Groups:* We invited group participants to return the week one week following the final session of the program, to participate in a focus group (N = 6 focus groups). The focus groups were led by an independently trained research assistant (THFK), alongside three facilitators including representatives from non-governmental organizations, with no more than two representatives per group. The size of the focus group was designed to be a minimum 6 and, a maximum 9, to ensure a group size that was suitable for facilitating a discussion. We randomly assigned parents from six different participating schools to ensure diverse representation within each group. The sampling method for the focus group discussions combined randomised and convenience sampling. The focus group was designed to last for an hour for adequate discussion over the four major themes. We developed a semi-structured interview guide to examine parents’ acceptability and perceived effectiveness of the intervention. The focus group interview guideline was developed accord to Kallio et al. (2016) [35]. Based on the existing literature, we identified four topic areas for examination: 1) sources of parental stress and difficulties in childcare; 2) mental health of parents and children; 3) social support; 4) program comments and areas of improvement. The discussions, which lasted between 58–97 minutes (with an average of 77 minutes), served as a platform for parents to voice their experiences and concerns in a structured yet open environment. The semi-structured interview guide used was instrumental in steering the discussions but was flexible enough to allow parents to express their thoughts freely and extensively.

### Outcome measures

#### Sociodemographic questionnaire.

The parents’ basic demographics, including age, gender, years of education, place of birth, religion, marital status, number of children, occupation, household income, and mental health were collected. In addition, their children’s mental health, class and academic performance were collected upon study entry.

#### Parenting stress.

The stress levels of parents were examined by rating sentences representing positive parenthood themes and negative components through the Parental Stress Scale (PSS-18; [36]. The PSS-18 consisted of 18 items, with the response options for each item ranging from 1 (strongly disagree) to 5 (strongly agree), with higher scores indicating greater parental stress. The Chinese version of PSS-18 was demonstrated to be a reliable (Cronbach’s alpha of.89) and valid tool for measuring parental stress in the Chinese population in Hong Kong [3 3,37, 38].

#### Parenting style.

Parenting styles were assessed by the Parenting Styles and Dimensions Questionnaire (PSDQ) [4,39, 40]. This shorter reworking version of the Parenting Practices Questionnaire contained 32 items measuring the degree of authoritative, authoritarian, and permissive parenting styles. It was categorized into seven sub-categories: permissive; physical coercion; non-reasoning/ punitive; verbal hostility; warmth and support; autonomy granting; and regulation). Each item is scored on a 5-point Likert scale anchored from 1 (never) to 5 (always). A higher score indicated a broader use of the corresponding parenting practices. The Chinese version of the PSDQ was shown to be a valid and reliable tool for assessing parenting styles in the Chinese population, with a internal consistency with Cronbach’s alpha values ranging from.625 to.884 and retest reliability with correlation values between.537 and.832. [41].

#### Parents’ Self-Efficacy.

The level of confidence of parents in confronting their children was measured using the General Self-Efficacy Scale (GSE) [42]. The GSE consists of 10 items. Each item is answered on a 4-point Likert scale ranging from 1 (not at all true) to 4 (exactly true), with higher scores indicating greater self-efficacy. The Chinese version of the GSE showed satisfactory reliability and internal consistency of.91 in the Chinese population [43].

#### Children’s mental health outcomes.

The Chinese version of Strengths and Difficulties Questionnaires (SDQ) was a valid and sensitive measure of treatment outcomes in children’s mental health [44]. Parents were required to identify if the listed items applied to their children by answering “not true”, “somewhat true”, and “certainly true”. It consists of 25 items, which could be sub-categorized into 5 classes: emotional symptoms, peer relationship problem, conduct problem, hyperactivity/ inattention, and prosocial behaviour. The overall difficulty scores were calculated by summarising the item scores of the first four classes excluding prosocial behaviour. The Chinese version of SDQ demonstrated a reliable and valid instrument for measuring children’s attributes in the Chinese population, with Cronbach’s alpha values greater than 0.8 for the total difficulties scale and the hyperactivity subscale [45].

#### Feedback surveys.

A program feedback form was developed to assess participants’ satisfaction rate, feasibility, and usefulness. It consisted of 10 statements including “the program met your expectations.”, “ the program had a suitable duration.”, “I will participate in the program again.”, “I will recommend other people to participate in this program.”, “Overall, I am satisfied with this course”, and the other 5 statements on the respective techniques the program equipped parents with. Each item was rated on a six-point Likert-type scale from 0 (strongly disagree) to 5 (strongly agree). The response was collected through an anonymous method. A score of 3–5 for each satisfaction rating was considered satisfactory.

#### Data analysis.

***Quantitative:*** All analyses were performed using IBM SPSS Statistics (version 29) software [46]. Missing data for the outcome measures were handled by multiple imputations [47]. Descriptive statistics were utilized to summarize the sociodemographic characteristics of the participants, including frequencies and percentages for categorical data (e.g., gender, marital status, housing type), and means and standard deviations for continuous data (e.g., age, number of children, monthly household income). Since all participating parents were anonymized, all pre-post score differences, including stress, self-efficacy, parenting styles, and children’s attributes, were determined by two-tailed between-group independent sample t-tests. Given that individual parents were not identifiable in the dataset, this approach ensured confidentiality. *T*-tests were chosen to assess changes in parental stress, self-efficacy, parenting styles, and children’s mental health as measured by the Parental Stress Scale (PSS-18), General Self-Efficacy Scale (GSE-10), Parenting Styles and Dimensions Questionnaire (PSDQ), and Strengths & Difficulties Questionnaire (SDQ). Changes in specific parenting styles (permissive, authoritarian, and authoritative) were analyzed using t-tests to identify significant shifts towards more or less favourable parenting practices. For children’s mental health, the total difficulties score of the SDQ was a key outcome, with further analyses on sub-scales like emotional symptoms, peer relationship problems, conduct problems, hyperactivity/inattention, and prosocial behaviour. The mean (M) and standard deviation (SD) at baseline and follow-up for each measure were calculated, and *t*-tests were performed to determine statistically significant differences with a significance level set at *p* < 0.05. To understand the predictors of parental stress, a multiple linear regression analysis was conducted. This model included general self-efficacy, parenting styles, and SDQ scores as predictors. We reported regression coefficients (b), 95% confidence intervals, and *p*-values to indicate the strength and significance of each predictor (Table 5). The adjusted R^2^ value was provided to show the proportion of variance in parental stress explained by the model. To explore the correlations between sociodemographic variables (e.g., education status, housing, income) and psychological outcomes (PSS, GSE, SDQ scores), Pearson’s correlation was used, with correlation coefficients (r) and p-values calculated to determine the strength and significance of these relationships. Service satisfaction was quantitatively assessed by calculating the percentage of participants who rated their satisfaction as satisfactory (scores of 3–5) on the feedback surveys. Additionally, attendance records were analyzed as a proxy for engagement and satisfaction with the PEMH program, offering further context to the subjective feedback provided by participants.

***Qualitative*:** In this study, we employ the principle of content analysis to systematically analyze the textual data gathered from our focus groups, based on the semi-structured interview on “Parental Challenges,” “Mental Health Concerns,” “Perceived Support,” and “Feedback on PEMH Program”. We integrated the data to form a coherent picture of the overarching narratives. This step involved comparing and contrasting the categories to draw meaningful conclusions about the data. Specifically, three researchers independently coded the data to identify key themes and categories. To address cases of disagreement when assigning statements to categories, we held regular meetings to discuss and resolve discrepancies through consensus. If consensus could not be reached, a fourth researcher was consulted to make the final decision. This collaborative approach ensured the reliability and validity of our content analysis. To enrich the analysis and provide authenticity, direct quotes were selectively used to illustrate and support the thematic findings. This approach ensures that the participants’ voices are heard, providing depth to the narrative while maintaining scientific rigor. To enhance the trustworthiness of our analysis, we employed techniques of peer debriefing to confirm the accuracy of the interpretations. The peer debriefing procedure was carried out systematically to ensure the accuracy and trustworthiness of our analysis. Specifically, after the initial coding and thematic analysis, the results were shared with a group of external experts (including child psychiatrists, nurse, social workers and teachers) who were not involved in the data collection process. A structured protocol was followed during the debriefing sessions, which included guidelines for reviewing the data, providing feedback, and discussing any discrepancies. Meetings were held to discuss the feedback and incorporate the suggestions into the final analysis [48].

## Results

By the end of the 2020–2022 academic year, 2246 parents across the six dedicated primary schools have participated in the PEMH program. Among those, 1771 of them completed the baseline questionnaire, resulting in a baseline response rate of 78.9%. After excluding duplicate entries and incomplete responses, a total of 1393 (78.7%) valid responses were included in the final analyses.

Table 3 describes the sociodemographic characteristics of the participants. Of the respondents who participated in the study, 84.4% *(n* = 1169) were mothers, and 15.6% were fathers. Almost 90% of parents were aged between 31 and 50 years, with a mean age of 39.90 (SD = 8.01). One-third (33.2%) obtained a Bachelor’s degree or above. More than one-third of participants (37.4%) reported a monthly household income of less than HKD$20,000, with 36.5% having full-time work. About half (51.7%) of the sample had two children, yet 7.8% of overall respondents were divorced or separated, and 6.1% were not living with a partner or family. The mean age of children was 8.08 (SD = 3.25), with slightly more reported males (51.5%). Regarding the health profile, 5.4% of participants had a personal history of mental disorders, and 2.0% reported that their children had mental disorders.

**Table 3:** Sociodemographic characteristics.

	Overall (*n* = 1393)	
	*N*	%
Gender		
Male	216	15.6
Female	1169	84.4
Age		
18–30	37	2.8
31–40	638	48.5
41–50	572	43.4
51 or above	70	5.3
Marital status		
Married/ In relationship	1226	92.2
Divorced/ Separated/ Widowed	104	7.8
Number of child(ren)		
1	481	38.4
2	648	51.7
3	110	8.8
4 or more	14	1.1
Education level		
Elementary or below	67	4.9
High School/ College	852	61.9
University or higher	457	33.2
Housing		
Private	616	46.4
Home Ownership Scheme	121	9.1
Public	392	29.5
Others	198	14.9
Living status		
Living with Partner	623	47.1
Living with family	619	46.8
Living alone/ Others	81	6.1
Number of persons living together		
2 persons	37	3.0
3 persons	304	24.5
4 persons	481	38.7
5 persons or more	419	33.8
Occupation status		
Full-time	485	36.5
Part-time	113	8.5
Homemaker/ Unemployed/ Retired	731	55.0
Monthly household income (HK$)		
< 20000	476	37.4
20000–39999	336	26.4
40000–59999	127	10.0
≥ 60000	334	26.2
Sufficient income for daily needs		
Yes	909	70.5
No	381	29.5
Family history on mental disorders		
Yes	46	3.6
No	1247	96.4
Personal history on mental disorders		
Yes	70	5.4
No	1232	94.6
Child’s gender		
Male	645	51.5
Female	608	48.5
Child’s age		
5 or below	28	2.3
6–8	717	59.6
9–11	422	35.0
12 or above	37	3.1
Child’s history on mental disorders		
Yes	25	2.0
No	1235	98.0

*n* = total number of participants. Only valid responses were included.

The follow-up results were collected after the two-year intervention. There were 2172 survey responses, with 1297 (59.7%) responses from parents who participated in the PEMH programme and fully completed the questionnaires; and 600 survey responses were obtained from parents who did not participate in the PEMH program. The pre-and post- scale scores of PSS, GSE, PSDQ, and SDQ with *t*-tests analyses were displayed in Table 4. Parents participating in the PEMH program exhibited significantly reduced parental stress, as evaluated by PSS (*t*(1297) = – 2.81, *p* = 0.005), and enhanced general self-efficacy, as indicated by GSE (*t*(1297) = 2.21, *p* = 0.027). Moreover, a shift towards an authoritative parenting style was observed. Parents adopted significantly fewer permissive (*t*(1297) = – 4.60, *p* < 0.001) and authoritarian (*t*(1297) = – 4.39, *p* < 0.001) parenting styles, and significantly more authoritative parenting styles (*t*(1297) = 3.43, *p* = 0.001), as measured by PSDQ. Furthermore, children’s mental health outcomes showed significant improvement, evidenced by a significant decrease in the total difficulties score of SDQ (*t*(1297) = − 3.12, *p* = 0.002).

**Table 4:** Independent sample *t*-tests comparing baseline and follow-up assessment scores.

	Baseline		Follow-up			
	**M**	**SD**	**M**	**SD**	***t***	***p*-Value**
Parental Stress Scale (PSS-18)	52.14	3.47	51.77	3.77	– 2.81	0.005**
General Self-Efficacy Scale (GSE-10)	23.38	5.14	23.81	5.31	2.21	0.027*
Parenting Styles and Dimensions Questionnaire (PSDQ)						
Permissive	2.58	0.56	2.49	0.57	– 4.60	<0.001***
Authoritarian	2.24	0.54	2.12	0.50	– 4.39	<0.001***
Physical coercion	1.90	0.63	1.84	0.57	– 2.72	0.022*
Non-reasoning/ Punitive	2.05	0.63	1.94	0.57	– 5.36	<0.001***
Verbal hostility	2.77	0.67	2.60	0.66	– 6.88	<0.001***
Authoritative	3.86	0.51	3.92	0.51	3.43	0.001**
Warmth and support	4.01	0.57	4.07	0.55	2.89	0.004**
Autonomy granting	3.71	0.57	3.80	0.56	4.12	<0.001***
Regulation	3.85	0.59	3.90	0.61	2.21	0.027*
Strengths & Difficulties Questionnaires (SDQ)						
Emotional symptoms	2.77	2.01	2.55	1.99	– 3.00	0.003**
Peer relationship problems	2.69	1.69	2.65	1.68	– 0.73	0.468
Conduct problems	2.34	1.44	2.35	1.58	0.12	0.908
Hyperactivity/ inattention	4.56	2.32	4.21	2.35	– 4.15	<0.001***
Prosocial behaviour	6.72	1.89	6.87	1.95	2.08	0.038*
Total difficulties	12.37	5.26	11.75	5.34	– 3.12	0.002**

* *p* < 0.05, ** *p* < 0.01, *** *p* < 0.001. M = mean; SD = standard deviation. *t*-tests (*t*) comparing baseline and follow-up data.

**Table 5:** Regressions Analyses for Predicting Parent Stress from Self-Efficacy, Parenting Styles and SDQ scores.

Variable	*b*	95% CI	*p*
General Self-Efficacy Scale (GSE-10)	−0.15	(−0.14, −0.07)	<0.001^***^
Parenting Styles (PSDQ)			
Permissive	0.12	(0.42, 1.19)	<0.001^***^
Authoritarian	0.16	(0.72, 1.64)	<0.001^***^
Authoritative	−0.07	(−0.90, −0.06)	0.25^*^
SDQ Total Difficulties	0.24	(0.13, 0.21)	<0.001^***^

R^2^_adj_ = 0.26, *p* < 0.001. CI = confidence interval.

Table 5 shows a multiple linear regression which shows the association of parental stress based on their general self-efficacy, parenting styles, and children’s mental health outcomes. Overall a significant regression was found (F (5, 1297) = 90.42, *p* < 0.001), with an adjusted R^2^ of 0.26. Parents with less self-efficacy, having parenting styles of either permissive or authoritarian, and higher self-rated SDQ scores would tend to have higher parental stress. While parents with an authoritative parenting style would have less tendency to have higher parental stress. All of the above psychological measures (GSE-10 scores, PSDQ scores, and SDQ scores) were significant predictors of parental stress (PSS-18).

Table 6 shows the analysis using Pearson’s correlation to evaluate the associations between the demographic factors and the psychological outcomes. Participants who were not living in private housing, currently unemployed, or perceived their monthly household income as not enough for living were correlated with higher parental stress. Parents with higher educational levels, living in private housing, having more persons living together, being employed, having higher household incomes, or perceiving their monthly income as adequate for living were positively correlated with general self-efficacy scores. On the other hand, parents with lower educational status, not living in private housing, lower household income, perceived their income as not enough for living, having either parents themselves or their children having psychiatric history, or having male children of younger age were positively correlated with higher SDQ total difficulties scores.

**Table 6:** Pearson’s Correlation on parents’ sociodemographic variables and their psychological attributes.

	PSS-18		GSE-10		SDQ	
Variable	*r*	*p*	*r*	*p*	*r*	*p*
Constant						
Parent’s Sex	0.05	0.055	−0.01	0.349	−0.01	0.378
Parent’s Age	−0.03	0.168	0.01	0.320	−0.03	0.142
Education Status	−0.03	0.181	0.14	< 0.001***	−0.11	< 0.001***
Marital Status	−0.03	0.124	−0.01	0.337	−0.02	0.223
No. of Children	0.03	0.156	0.02	0.257	−0.01	0.386
Housing (Private Estate)	0.06	0.030*	−0.12	< 0.001***	0.11	< 0.001***
Living with Partners	0.00	0.441	−0.03	0.189	0.04	0.093
Persons Living Together	0.01	0.428	0.06	0.028*	−0.01	0.410
Working Status	0.06	0.025*	−0.06	0.023*	0.02	0.305
Household Income	−0.03	0.183	0.12	< 0.001***	−0.11	< 0.001***
Income enough for living?	−0.12	< 0.001***	0.11	< 0.001***	−0.12	< 0.001***
Personal History of Mental Disorders	0.04	0.110	−0.06	0.021*	0.07	0.013*
Child’s Sex	−0.03	0.127	0.05	0.065	−0.11	< 0.001***
Child’s Age	−0.01	0.353	0.03	0.127	−0.05	0.041*
Child Having Developmental Problems	0.04	0.074	−0.03	0.180	0.14	< 0.001***

Note. * *p* < 0.05, ** *p* < 0.01, *** *p* < 0.001.

### Service satisfaction

Feedback surveys revealed high satisfaction with the PEMH program (95.6%). Workshop satisfaction levels were 98.9% and 98.7% for levels 1 and 2, respectively, and talk satisfaction was reported at 99%. The program met expectations for 95.7% of participants, 95.6% would participate again, and 95.2% would recommend it to others. Up to 96.8% believed the PEMH program equipped them with stress management, developmental psychological needs of children, strengthening parent-child relationships, enhancing awareness, and boosting confidence in managing children’s mental health issues. Almost half of the parents attended at least one PEMH event, suggesting that parenting psychoeducation interventions like the PEMH program were feasible for working parents.

### Focus groups

We conducted 6 focus groups with 47 parents. Parents described parenting their child(ren), supporting their child(ren) with online learning, social norms/life pattern changes, arguments with children, exacerbated due to the COVID-19 pandemic, child(ren) not concentrating on study, lack of time to rest, were major sources of parental stress. Parents identified a wide range of mental health symptoms that fluctuated in their children’s behavior. They articulated low mood due to lack of personal time, anxiety about selecting a school for children, easily getting angry when arguing with children, and sleeping problems. Social support was generally described to be lacking: P8, a single parent, described, “ We need to find the resources by ourselves. NGOs lack promotion, there is not enough support for parents’ mental health issues.” P9, a mother of an 8-year-old girl shared, “I don’t know what platforms are available to provide workshops/ talks to relieve stress and emotions. We lack support in emotional support or parenting teaching.” The majority of participants enthusiastically shared that they enjoyed the PEMH program and appreciated the components of learning discipline and modern parenting skills in the PEMH program. However, participants advised improvements like face-to-face workshops and parenting strategies specifically for managing the confrontations with children due to work/study from home due to the COVID-19 pandemic.

## Discussion

The overall aim of the present study was to evaluate the initial effectiveness of a recently developed PEMH program, designed for parents of primary school-aged children and adolescents in Hong Kong. Our findings demonstrated significant reductions in parental stress and enhancements in parental self-efficacy. Notably, there was a shift towards a more favorable parenting style (albeit, a shift towards more authoritative parenting styles, characterized by reductions in both permissive and authoritarian practices), and mitigating children ’s mental health outcomes. Parental stress was significantly associated with self-efficacy, parenting styles, and children’s mental health outcomes, while socio-economic factors (including higher educational level and household income) were significantly associated with these parental and children’s mental health outcomes.

We observed significant alterations in parenting styles as a key objective of the PEMH program. While our findings do not definitively indicate a shift from permissive and authoritarian parenting towards authoritative parenting, the substantial reduction of permissive and authoritarian parenting styles could have positive implications for children’s psychosocial development. According to Baumrind’s parenting styles theory, authoritative parenting emphasizes the importance of balancing control and warmth in interactions with children. This parenting style is suggested to be universally optimal for supporting children’s development [49,50]. On the other hand, permissive environments tend to be associated with low levels of happiness, self-regulation, and authority problems in children; while authoritarian environments are linked to producing children with lower level of happiness, social competence, and self-esteem. Subsequently, neither permissive nor authoritarian parenting styles are recommended for optimal child development [51]. The observed changes in parenting styles towards a reduction in permissive and authoritarian approach post-PEMH program indicate potential positive effects on children’s well-being. By encouraging a more authoritative parenting style through the PEMH program, we aim to promote the development of healthy parent-child relationships and support children’s overall psychosocial functioning.

The findings of our study, which demonstrates improved parental self-efficacy, reduced parental stress, and improved children’s mental health outcomes, corroborated with the findings of past parental programs conducted in Hong Kong [11,28]. These findings have important implications for the development and implementation of effective parental training interventions, as they suggest these interventions can lead to improvements in both parent and child outcomes. The implementation of the PEMH program as an early intervention strategy for preschoolers holds significant promise. Early interventions are particularly valuable as they can contribute to optimal development and mental health by addressing early brain development [52], alleviating parental stress [53], and supporting families during a critical developmental period. By targeting this early stage, the PEMH program has the potential to promote long-term well-being for both parents and children [27].

Parental stress was significantly associated with self-efficacy, parenting styles, and perceptions of children’s difficulties, which aligns with previous studies (e.g., [20,54]). It is noteworthy that changes in parenting strategies may not directly lead to improvements in parental stress levels. Such strategies might be more likely to be employed and effective when there is a child conduct problem, and subsequently alleviate parental stress when the problem is managed. On the other hand, our observations indicate that parents with higher socioeconomic status (SES), including higher education level and household income, generally exhibited better parental and children outcomes from the PEMH program, consistent with the result of prior research parenting programs [26,55]. Parents with higher SES may possess higher levels of health literacy and access to resources, enabling better engagement with PEMH programs [56]. Conversely, parents with lower SES may encounter barriers such as limited resources, social support, and increased stress, hindering effective parental training [55]. However, these correlations do not imply causality, and other unaccounted factors may influence the observed relationships. Exploring strategies to enhance engagement and outcomes for parents with lower SES could be crucial for future success of parental education intervention [57] For instance, collaboration with community groups, such as parent-teacher associations, could provide additional support to parents with lower SES, including peer groups, mentoring initiatives, and access to resources [58]. By promoting these approaches, we can empower and support parents with lower SES, ultimately improving future PEMH program outcomes.

Feedback surveys and focus group results indicate that the PEMH program was generally acceptable and feasible. Notably, the fluctuation of parents’ mental health symptoms in response to their children’s behavior underscores the necessity for parents to comprehend the challenges their children face and maintain open lines of communication. Future research could examine how the program could be enhanced to better address these issues. Beyond acceptability, we observed that many parents perceived social support as insufficiently robust or specific, emphasizing the importance of social support in managing childcare stress and difficulties. The PEMH program might be enhanced by giving additional information on organisations that provide emotional and family support services. PEMH’s strengths includes offering discipline skills, parenting skills, and contemporary parenting practices, while offering flexible workshop and educational session choices. In future iterations of the program, these strengths could be enhanced and expanded.

Despite the use of validated measures with focus groups to evaluate the newly developed PEMH program with a relatively broad base of participants, several limitations warrant caution in interpreting the results. First, the use of an electronic platform for data collection could have led to a lower attribution rate without direct assessment from program contributors. Second, the concurrent COVID-19 pandemic during the PEMH implementation period (2021–2022) may have confounded the effects. Third, the off-label design and voluntary nature of the study may have limited the statistical power due to fewer survey responses collected post-program. Fourth, due to the Chinese social climate, where good news is reported but bad news is often ignored, parents may alter their responses to questions.

In conclusion, our study affirms the effectiveness of parental education in mental health. The PEMH program demonstrates a monumental potential in strengthening parenting outcomes and enhancing children’s well-being through increasing positive parental involvement in their children’s education, behavior, and well-being. This, in turn, helps alleviate parental stress and promote healthy family relationships. This study serves as a cornerstone in the field of parent education with respect to the importance of implementing evidence-based interventions like PEMH into primary schools and expanding the scope of early parental psychoeducational interventions for pre-school children.

## Supporting information

S1 File Exploration of the New Parent educaiton program. (PDF)

S2 File PEMH 2020-22_fulldataset_raw. (XLSX)
