# Supplementary material for: The effectiveness of a new parent education intervention program for primary school-age children and parents in Hong Kong
Source: PLoS One. 2025 Sep 15;20(9):e0331308. doi: 10.1371/journal.pone.0331308 (PMC12435718; doi:10.1371/journal.pone.0331308)
Supplement: S1 File — (PDF) [file pone.0331308.s001.pdf]

THE HONG KONG 香 港 醫 訊

MEDICAL DIARY

www.fmshk.org

VOL.27 NO.5 May 2022

Child Psychiatry

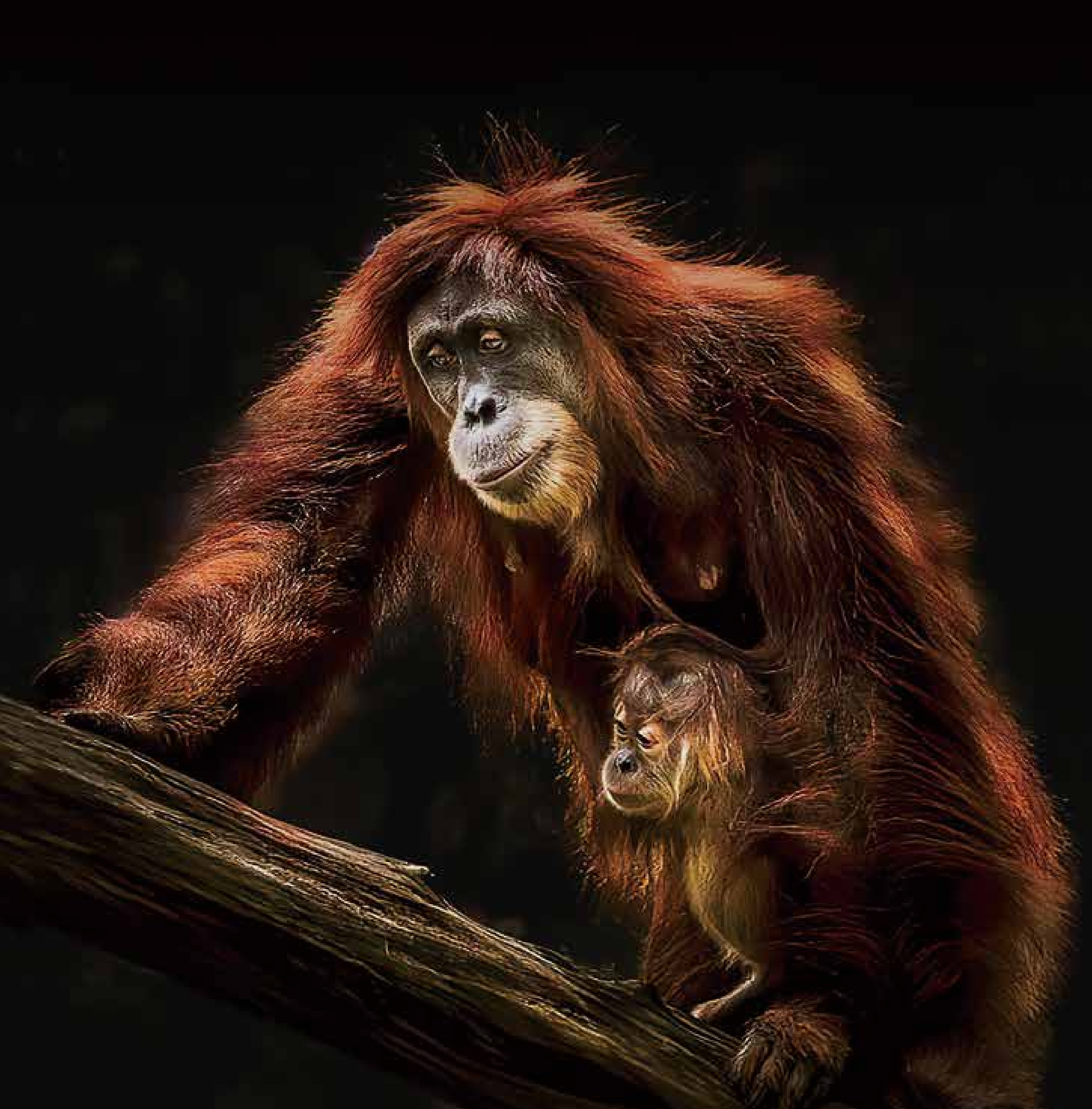

OFFICIAL PUBLICATION FOR THE FEDERATION OF MEDICAL SOCIETIES OF HONG KONG ISSN 1812 - 1691

Lexapro®

the power to tackle depression / anxiety at its core

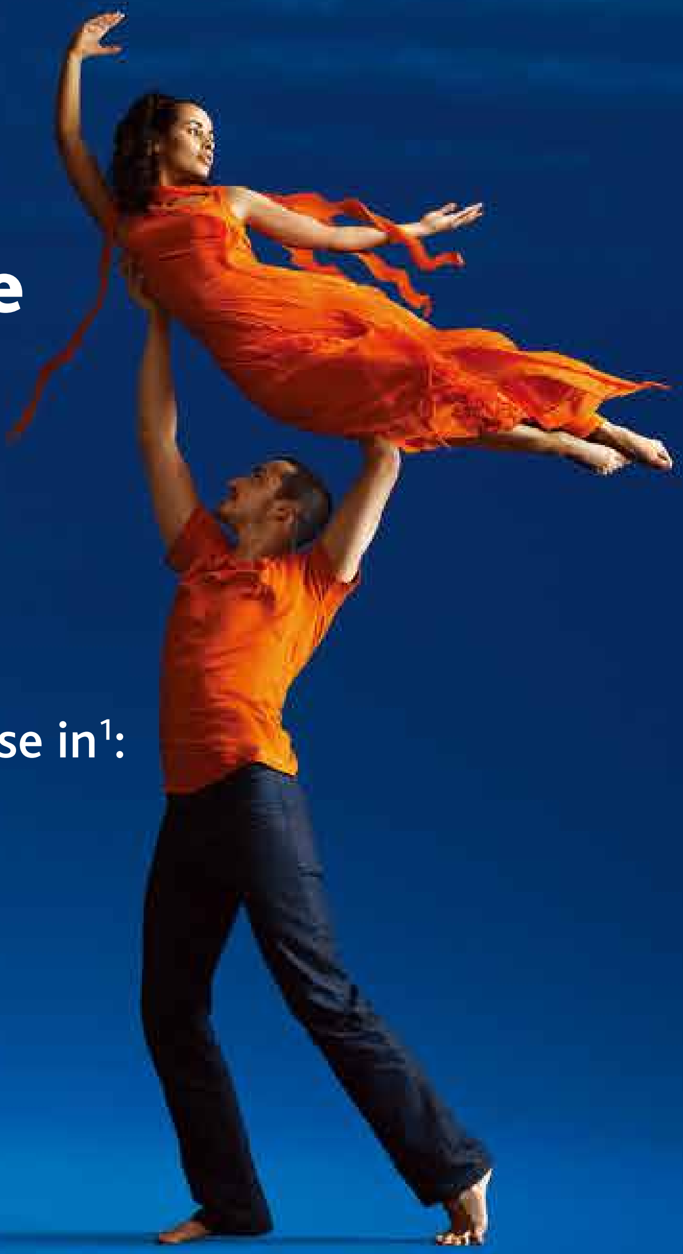

Lexapro® is approved for use in[1]:

- Major Depressive Disorder (MDD)
- Generalized Anxiety Disorder (GAD)
- Social Anxiety Disorder (SAD)
- Panic Disorder (PD)
- Obsessive-Compulsive Disorder (OCD)

Reference: 1. Lexapro® Summary of Product Characteristics. May 2017.

Lexapro Abbreviated Prescribing Information
Presentation: Escitalopram Film-coated tablets 5 mg, 10 mg, 15 mg, 20 mg. **Indication:** Treatment of major depressive episodes, Treatment of panic disorder with or withoutagoraphobia, Treatment of social anxiety disorder (social phobia), Treatment of generalised anxiety disorder, Treatment of obsessive-compulsive disorder (OCD). **Dosage** Adults: Usual dosage is 10 mg once daily. Depending on individual patient response, the dose may be increased to a maximum of 20 mg daily. An initial starting dose of 5 mg once daily is recommended in the treatment of elderly (>65 years), in panic disorder and in patients with reduced hepatic function. Children and adolescents (<18 years): Lexapro should not be used. Discontinuation: Discontinue gradually over a period of at least one to two weeks to reduce the possibility of discontinuation symptoms. **Contraindications:** Concomitant treatment with non-selective, irreversible monoamine oxidase inhibitors (MAO-inhibitors). Patients with known QT interval prolongation or congenital long QT syndrome. Concomitant treatment with pimozide. Should not be used during pregnancy unless clearly needed and after careful consideration of the risk/benefit ratio. Breast-feeding is not recommended. **Precautions:** Depression is associated with an increased risk of suicidal thoughts, self-harm and suicide. It is a general clinical experience that the risk of suicide may increase in the early stages of recovery. Close supervision of patients and in particular those at high risk should accompany drug therapy especially in early treatment and following dose changes. Patients (and caregivers) should be alerted about the need to monitor for any clinical worsening, suicidal behaviour or thoughts and unusual changes in behaviour and to seek medical advice immediately if these symptoms present. SSRIs should be avoided in patients with unstable epilepsy, and patients with controlled epilepsy should be closely monitored. SSRIs should be used with caution in patients with a history of mania/hypomania. SSRIs should be discontinued in any patient entering a manic phase. Treatment with SSRIs may alter glycaemic control. Insulin and/or oral hypoglycaemic dosage may need to be adjusted. The use of SSRIs/SNRIs has been associated with the development of akathisia. Hyponatraemia has been reported rarely with the use of SSRIs. There have been reports of cutaneous bleeding abnormalities. Escitalopram has been found to cause a dose-dependent prolongation of the QT interval. **Interactions:** Caution is advised when taken in combination with MAO-inhibitors or medicinal products known to prolong the QT interval, serotonergic medicinal products, products lowering the seizure threshold, lithium, tryptophan, St. John's Wort, oral anticoagulants or antiplatelet agents (NSAIDs), and products predominantly metabolised by the enzymes CYP2C19, CYP3A4 and CYP2D6. **Undesirable effects:** Adverse reactions are most frequent during the first or second week of treatment and usually decrease in intensity and frequency with continued treatment. Very common: Nausea. Common: Decreased/increased appetite, weight increased, anxiety, restlessness, abnormal dreams, libido decreased, anorgasmia, insomnia, somnolence, dizziness, paraesthesia, tremor, sinusitis, yawning, diarrhoea, constipation, vomiting, dry mouth, sweating increased, arthralgia, myalgia, ejaculation disorder, impotence, fatigue and pyrexia. Uncommon: weight decreased, bruxism, agitation, nervousness, panic attack, confusional state, taste disturbance, sleep disorder, syncope, mydriasis, visual disturbance, tinnitus, tachycardia, epistaxis, gastrointestinal haemorrhages (including rectal haemorrhage), urticaria, alopecia, rash, pruritus, metrorrhagia, menorrhagia, oedema. Rare: anaphylactic reaction, aggression, depersonalisation, hallucination, serotonin syndrome, bradycardia. Not known: thrombocytopenia, inappropriate ADH secretion, hyponatraemia, anorexia, mania, suicidal ideation, suicidal behaviour, dyskinesia, movement disorder, convulsion, psychomotor restlessness/akathisia, electrocardiogram QT prolonged, orthostatic hypotension, hepatitis, liver function test abnormal, ecchymosis, angioedemas, urinary retention, galactorrhoea, priapism. Class effects: Epidemiological studies, mainly conducted in patients 50 years of age and older, show an increased risk of bone fractures in patients receiving SSRIs and TCAs. **Overdose:** Cases of overdose with escitalopram doses between 400 mg and 800 mg have been reported without any severe symptoms. **Legal category:** POM. **Date of preparation or last review** May 2017.

Further information is available upon request

Lundbeck HK Limited
Suite 4303, Central Plaza,
18 Harbour Road, Wanchai, Hong Kong
Tel: 2244 8888 www.lundbeck.com

Lundbeck

Lexapro®
escitalopram

HK-LU-2021.06-AD02

Contents

Editorial

- **Editorial** 2
  Dr Phyllis KL CHAN

Medical Bulletin

- **Exploration of the New Parent Education in Mental Health Programme (PEMH) at Hong Kong Primary Schools - Interim Results** CME 5
  Dr Calvin PW CHENG, Mr Tommy KH FONG & Dr May LAM
- **MCHK CME Programme Self-assessment Questions** 11
- **Practice of Parent Education from Experience at the 3 Tier Levels: at Clinic, School and Community** 13
  Ms Wai-ping CHAN
- **The Happy Kids Charter - Practical Tips to Promote Happy and Holistic Development in Children** 16
  Dr Krystal CK LEE & Dr Phyllis KL CHAN
- **Frontline Social Workers' Experience in the Implementation of the Parent Education in A Mental Health Project at Primary Schools** 20
  Ms Yick-man LAM, Ms Bubble SW LEE & Ms Mei-ling WONG
- **The Impact of Covid-19 on the Mental Health of Primary School Parents in Hong Kong and Overseas** 24
  Dr Vincent YC TANG, Dr Krystal CK LEE & Dr Roger NG

Lifestyle

- **Inspecting the World in Another Perspective - The World of Macrophotography** 27
  Dr Victor NS WONG

Dermatology Quiz

- **Dermatology Quiz** 18
  Dr Lai-yin CHONG

Medical Diary of May 31

Calendar of Events 32

Scan the QR-code

To read more about The Federation of Medical Societies of Hong Kong

Disclaimer

All materials published in the Hong Kong Medical Diary represent the opinions of the authors responsible for the articles and do not reflect the official views or policy of the Federation of Medical Societies of Hong Kong, member societies or the publisher.

Publication of an advertisement in the Hong Kong Medical Diary does not constitute endorsement or approval of the product or service promoted or of any claims made by the advertisers with respect to such products or services.

The Federation of Medical Societies of Hong Kong and the Hong Kong Medical Diary assume no responsibility for any injury and/or damage to persons or property arising from any use of execution of any methods, treatments, therapy, operations, instructions, ideas contained in the printed articles. Because of rapid advances in medicine, independent verification of diagnoses, treatment method and drug dosage should be made.

The Cover Shot

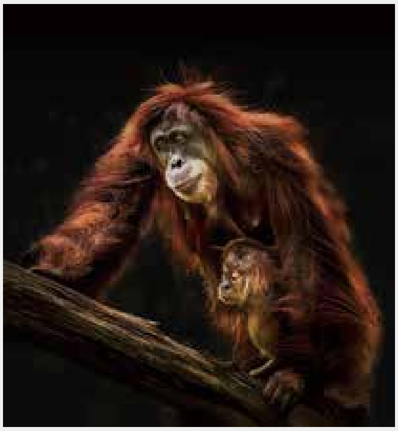

This is a photograph of a female orangutan climbing up the branches with her little child.

Orangutans are great apes native to the rainforests of Indonesia and Malaysia. They are now found only in parts of Borneo and Sumatra. They have been kept in captive facilities around the world since at least the early 19th century. (https://en.wikipedia.org/wiki/Orangutan)

Orangutans are among the most intelligent primates. They use a variety of sophisticated tools and construct elaborate sleeping nests each night from branches and foliage. Fruit is the most important component of an orangutan's diet, but they will also eat vegetation, bark, honey, insects and bird eggs. (https://en.wikipedia.org/wiki/Orangutan)

The mothers and babies have an incredibly close relationship. In the wild, orangutans stay with their mothers until they are around seven or eight years old. They are generally non-aggressive toward humans and each other.

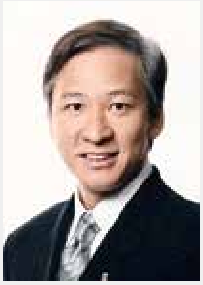

Dr Victor NS WONG
FRCS, FHKCORL, DLORCS, MB, BS

Published by
The Federation of Medical Societies of Hong Kong

EDITOR-IN-CHIEF
Dr CHAN Chun-kwong, Jane
陳真光醫生

EDITORS
Prof CHAN Chi-fung, Godfrey
陳志峰教授 *(Paediatrics)*
Dr CHAN Chi-kuen
陳志權醫生 *(Gastroenterology & Hepatology)*
Dr KING Wing-keung, Walter
金永強醫生 *(Plastic Surgery)*
Dr LO See-kit, Raymond
勞思傑醫生 *(Geriatric Medicine)*

EDITORIAL BOARD
Dr AU Wing-yan, Thomas
區永仁醫生 *(Haematology and Haematological Oncology)*
Dr CHAK Wai-kwong
翟偉光醫生 *(Paediatrics)*
Dr CHAN Hau-ngai, Kingsley
陳厚毅醫生 *(Dermatology & Venereology)*
Dr CHAN, Norman
陳諾醫生 *(Diabetes, Endocrinology & Metabolism)*
Dr CHEUNG Fuk-chi, Eric
張復熾醫生 *(Psychiatry)*
Prof CHEUNG Man-yung, Bernard
張文勇教授 *(Clinical Pharmacology)*
Dr CHIANG Chung-seung
蔣忠想醫生 *(Cardiology)*
Prof CHIM Chor-sang, James
詹楚生教授 *(Haematology and Haematological Oncology)*
Dr CHONG Lai-yin
莊禮賢醫生 *(Dermatology & Venereology)*
Dr CHUNG Chi-chiu, Cliff
鍾志超醫生 *(General Surgery)*
Dr FONG To-sang, Dawson
方道生醫生 *(Neurosurgery)*
Dr HSUE Chan-chee, Victor
徐成之醫生 *(Clinical Oncology)*
Dr KWOK Po-yin, Samuel
郭寶賢醫生 *(General Surgery)*
Dr LAM Siu-keung
林兆強醫生 *(Obstetrics & Gynaecology)*
Dr LAM Hiu-yin, Sonia
林曉燕醫生 *(Radiology)*
Dr LEE Kin-man, Philip
李健民醫生 *(Oral & Maxillofacial Surgery)*
Dr LEE Man-piu, Albert
李文彪醫生 *(Dentistry)*
Dr LI Fuk-him, Dominic
李福謙醫生 *(Obstetrics & Gynaecology)*
Prof LI Ka-wah, Michael, BBS
李家驊醫生 *(General Surgery)*
Dr LO Chor Man
盧礎文醫生 *(Emergency Medicine)*
Dr LO Kwok-wing, Patrick
盧國榮醫生 *(Diabetes, Endocrinology & Metabolism)*
Dr MA Hon-ming, Ernest
馬漢明醫生 *(Rehabilitation)*
Dr MAN Chi-wai
文志衛醫生 *(Urology)*
Dr NG Wah Shan
伍華山醫生 *(Emergency Medicine)*
Dr PANG Chi-wang, Peter
彭志宏醫生 *(Plastic Surgery)*
Dr TSANG Kin-lun
曾建倫醫生 *(Neurology)*
Dr TSANG Wai-kay
曾偉基醫生 *(Nephrology)*
Dr YAU Tsz-kok
游子覺醫生 *(Clinical Oncology)*
Prof YU Chun-ho, Simon
余俊豪教授 *(Radiology)*
Dr YUEN Shi-yin, Nancy
袁淑賢醫生 *(Ophthalmology)*

Design and Production
A-PRO MULTIMEDIA LTD www.apro.com.hk

Editorial

Dr Phyllis KL CHAN

MBBS(HK), FRC Psych (UK), FHKC Psych,
FHKAM (Psychiatry)

Consultant Psychiatrist (partime), Queen Mary Hospital &
Kwai Chung Hospital
Honorary Clinical Associate Professor, Department of Psychiatry,
LKS Faculty of Medicine, The University of Hong Kong
Vice-President (General Affairs), The Hong Kong College of Psychiatrists

Editor

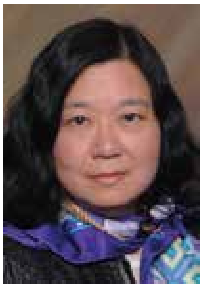

Dr Phyllis KL CHAN

Child and adolescent mental health has been the focus of psychiatric service in the past several years. The ever across-the-board increase in referrals and long waiting time of over two years for non-urgent cases at the Hospital Authority (HA) Child and Adolescent Psychiatric Clinics, coupled with (1) the alarming cluster of student suicides in 2015-16, (2) the recent surge of student suicide again after the physical school term began in Sept 2021, and (3) mental health concerns over children and adolescents during Covid time, have put Child and Adolescent Psychiatry in the limelight.

According to the statistics from the Hong Kong Jockey Club Centre for Suicide Research and Prevention[1], suicide rates for full-time students increased by 76% between 2012 and 2016. Of the 75 youth suicides in 2016, 29 were presumed to be full-time students. The report from the expert group, Committee on Prevention of Student Suicide of the Education Bureau (EDB)[2], released in November 2016, has addressed a long list of factors which have interacted and contributed to students' suicidal behaviours, including mental health issues, psychological concerns, family relationship and adjustment problems, peer relationship problems, school adjustment problems, and academic stress.

The report also showed that 50% of Hong Kong students and teachers show signs of depression arising from the stress of school workload.

Not only students are stressed, but so are their carers. Of the 509 parents of kindergarten, primary and secondary children polled by the Hong Kong Institute of Family Education[3] from April to May 2019, 22.4% said they were under a lot of pressure. The main causes of the stress were their children's emotions (29.1%) and studies (27.7%). A total of 11.9% parents said they spent two hours or more each day with their children on homework while 10.2% did not dedicate any of the day talking to their children in the previous three months.

The Child and Adolescent Mental Health Service in Hong Kong are under-resourced. According to WHO[4], up to 20% of children and adolescents worldwide suffer from a disabling mental illness. The WHO[5] estimates that about 4-6% of children who have distressing or disabling psychiatric difficulties are in need of clinical intervention. In Hong Kong, the overall prevalence estimate of mental disorders among the sampled adolescents was 16.4%[6] (Leung et al., 2008). The public healthcare system of Hong Kong also observes a growing number of children and adolescents diagnosed with mental problems. As one indication, the caseload of the Child and Adolescent Psychiatric Team of the HA rose from 18,900 in 2011-12 to 28,800 in 2015-16, representing an increase by more than 50%.

The position statement of the Child and Adolescent Psychiatry clinical division of the Hong Kong College of Psychiatrists[7] has highlighted the priorities in managing this complex problem. Adequate resources in current psychiatric services, in tier-2 (out-reach child and adolescent mental health team at school) and also in tiers 3 and 4 (specialist child and adolescent psychiatric out-patient clinic, day hospital and in-patient services) are needed to ensure efficient and effective services for those identified patients who need timely psychiatric intervention.

Currently effort is being put into the pilot "Student Mental Health Support Scheme"[8] under Food and Health Bureau (FHB), in which child and adolescent psychiatric mental health team from the HA including advanced practice nurses (APN), clinical psychologists, and social workers are deployed to provide tier-2 school-based service to students with previously known or unknown mental issues in collaboration with school professionals such as school social workers, school guidance teachers and educational psychologists. This service has already been rolled out to over 200 schools and is being evaluated on how to be rolled out hopefully territory-wide to all eligible schools in Hong Kong effectively.

Before the titanic change in education and school system as well as in mental health policy and service could be made, changes in the mentality of parents, the public and all of us on mental illness could be fostered. Destigmatisation of mental illness is the uphill arduous battle we have to fight, and is the main theme of the College's Public Awareness Committee's[9] public education work as destigmatisation of mental illness could minimise obstacles to access to service, enhance medication adherence and facilitate voluntary hospitalisation of patients at high risk or with severe mental illness.

There is a service gap and societal need in having a systematic and evidence-based parenting education, as stated in recommendations made by various task forces and expert panel groups. The recommendations by the Committee on Prevention of Student Suicide[2] (Final Report, November 2016), in line with the WHO guiding principles, are directed towards three levels, universal, selective and indicated prevention. Family life and parent education (FL&PE), in particular on positive parenting, should be more widely promoted and delivered. The FL&PE should be based on a theoretically sound and empirically validated framework, covering developmental, preventive and remedial levels of work and targeting families in different stages of development on attitude, knowledge and skills.

One important mental health work that mental health professionals could do at this stage with impact is developing and implementing a parent education in mental health (PEMH) programme, in line with the EDB's ongoing work on setting up the framework for parent education for various age group students, from kindergarten[10] to primary school and secondary school.

Children are our future. Child and adolescent mental health is an essential and important part of overall health. Good parent education on mental health and thus ensuring a good start in the child's life will help children and adolescents develop lifelong resilience to adversity and to develop their full potential. There are different stages of child and adolescent development, each stage being associated with varying physical, social, emotional and mental health need. Thus, evidence-based and tailor-made Parent Education in Mental Health (PEMH) according to different developmental stages is very important.

Parenting is co-related to many different outcomes and various studies have shown that parenting is related to many different outcomes in child and adolescent development:

- Good parenting has wide-ranging benefits for the child, from better cognitive and psychosocial development to protection from disease and mortality, resulting in better child health and development, especially for the neediest populations[11] (WHO, 2006)
- Parenting has a pervasive impact on children's development[12] (Collins et al., 2000)
- The impact of parenting on child development includes: good mental and physical health, healthy lifestyles, the ability to confidently engage with others, and the development of self-esteem and a positive sense of self[13] (AAPCH, 2017)

In this issue, Dr Calvin CHENG illustrated with his one-year data how well the programme of parent education in mental health worked. Dr Krystal LEE presented the "Happy Kids Charter" Project[14] questionnaire research asking school representatives and parents on their perception of the student stress index and concept of Happy Kids Charter for primary school children. Dr Vincent TANG reviewed the study commissioned by the Hong Kong College of Psychiatrists on the mental health well-being of parents of special educational need students. The paper by nurse consultant Miss W P CHAN and non-governmental organisation (NGO) colleagues shared with us their frontline experience in conducting parent education at different tier levels as well as their experience in conducting the 'Parent Education in Mental Health' programme at schools, especially during Covid time. The lifestyle page by Dr Victor WONG on macro-photography is highly professional and intriguing but at the same time easy to read and understand.

References

1. The Hong Kong Jockey Club Centre for Suicide Research and Prevention, https://csrp.hku.hk
2. Final Report of "The Committee on Prevention of Student Suicides", https://www.edb.gov.hk/attachment/en/student-parents/crisis-management/about-crisis-management/CPSS_final_report_en.pdf
3. Hong Kong Institute of Family Education, http://www.ife.org.hk/
4. Caring for Children and Adolescents with Mental Disorder, WHO, 2003.
5. Atlas, Child and Adolescent Mental Health Resources, WHO, 2005.
6. Leung, et al (2008). Prevalence of DSM-IV disorders in Chinese adolescents and the effects of an impairment criterion: a pilot community study in Hong Kong. Eur Child Adolesc Psychiatry, 17(7), 452-61.
7. Position Statement of Student Suicide (English version), Hong Kong College of Psychiatrists, http://www.hkcpsych.org.hk/index.php?option=com_docman&task=doc_download&gid=3281&Itemid=332&lang=en
8. Student mental health support scheme, https://mentalhealth.edb.gov.hk/en/support-programmes/student-mental-health-support-scheme.html
9. The public awareness committee, the Hong Kong College of Psychiatrists https://www.hkcpsych.org.hk/index.php?option=com_content&view=article&id=84&Itemid=306&lang=en
10. Curriculum Framework on Parent Education (Kindergarten); Education Bureau, Sept 2021
11. World Health Organization, Bulletin of the World Health Organization: Bulletin de L'Organisation Mondiale de la Santé. 2006: World Health Organization.
12. Collins, et al (2000). Contemporary research on parenting: The case for nature and nurture.
13. Australasian Association of Parenting & Child Health, https://www.aapch.com.au/
14. Happy Kids Charter, https://www.edb.gov.hk/attachment/en/teacher/prevention-of-student-suicides/Family_1.pdf

Restore Patients' Functioning from Depression

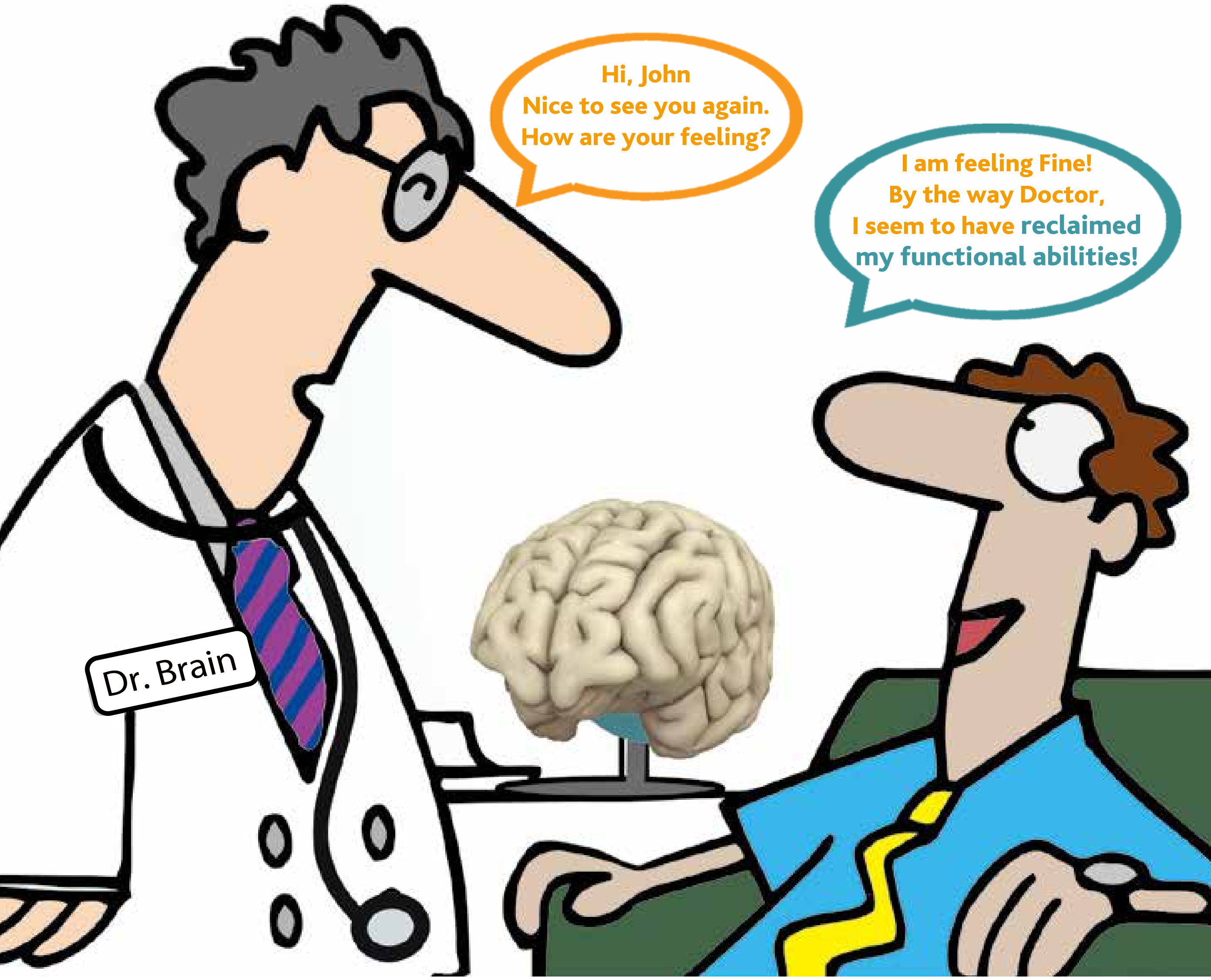

BRINTELLIX® (VORTIOXETINE) – ABBREVIATED PRESCRIBING INFORMATION
Brintellix®: Active Substance: Vortioxetine Hydrobromide. **Presentation:** Film-coated tablets 5mg, 10mg and 20mg. **Indication:** Treatment of major depressive episodes in adults. **Dosage:** Adults: starting and recommended dose is 10mg, once-daily, taken with or without food. Elderly ≥65 years: Starting dose 5mg. Children and adolescents (<18 years): should not be used. Discontinuation: Patients can abruptly stop taking the medicinal product without the need for a gradual reduction in dose. **Contraindications:** Hypersensitivity to vortioxetine or to any of the excipients. Combination with MAO-inhibitors. Should not be used during pregnancy or lactation unless clearly needed and after careful consideration of the risk/benefit. **Special warnings and precautions:** Depression is associated with an increased risk of suicidal thoughts, self-harm and suicide. It is a general clinical experience that the risk of suicide may increase in the early stages of recovery. Close supervision of high-risk patients should accompany drug therapy. Patients (and caregivers) should be alerted about the need to monitor for any clinical worsening, suicidal behavior or thoughts and unusual changes in behaviour and to seek medical advice immediately if these symptoms present. Should be introduced cautiously in patients who have a history of seizure or in patients with unstable epilepsy. Patients should be monitored for the emergence of signs and symptoms of Serotonin Syndrome or Neuroleptic Malignant Syndrome. Should be used with caution in patients with a history of mania/hypomania and should be discontinued in any patient entering a manic phase. Patients treated with antidepressants, including vortioxetine, may also experience feelings of aggression, anger, agitation and irritability. Patient's condition and disease status should be closely monitored. There have been reports of cutaneous bleeding abnormalities with the use of SSRIs/SNRIs. Hyponatraemia has been reported rarely with the use of SSRIs/SNRIs. Mydriasis has been reported in association with use of antidepressants, including vortioxetine. This mydriatic effect has the potential to narrow the eye angle resulting in increased intraocular pressure and angle-closure glaucoma. Caution should be exercised for patients with renal or hepatic impairment. **Interactions:** Caution is advised when taken in combination with MAO-inhibitors, serotonergic medicinal products, products lowering the seizure threshold, lithium, tryptophan, St. John's Wort, oral anticoagulants or antiplatelet agents, and products predominantly metabolised by the enzymes CYP2D6, CYP3A4, CYP2C9, and Cytochrome P450. There have been reports of false positive results in urine enzyme immunoassays for methadone in patients who have taken vortioxetine. **Undesirable effects:** Very common: Nausea. Common: abnormal dreams, dizziness, diarrhoea, constipation, vomiting, pruritus, including pruritus generalised. Uncommon: flushing, night sweats. Rare: Mydriasis (which may lead to acute narrow angle glaucoma). Not known: Anaphylactic reaction, Hyponatraemia, Insomnia, Serotonin Syndrome, Haemorrhage (including contusion, ecchymosis, epistaxis, gastrointestinal or vaginal bleeding), Angioedema, Urticaria, Agitation, Aggression, Rash. **Overdose:** Symptomatic treatment. The most frequently reported symptoms were nausea and vomiting for overdoses of up to 80 mg and seizure and serotonin syndrome for overdoses above 80 mg. **Legal category:** POM. **Marketing Authorisation Holder:** Lundbeck HK Limited, Suite 4303, Central Plaza, 18 Harbour Road, Wanchai, Hong Kong. **Revision Date:** Jan 2021 based on HK SmPC dated Sep 2020. **Full prescribing information is available upon request.**

HK-LU-2021.09-AD01

Lundbeck

Lundbeck HK Limited
Suite 4303, Central Plaza,
18 Harbour Road, Wanchai, Hong Kong
Tel: 2244 8888 www.lundbeck.com

Thanks to

Brintellix®
vortioxetine

Exploration of the New Parent Education in Mental Health Programme (PEMH) at Hong Kong Primary Schools - Interim Results

Dr Calvin PW CHENG
MBBS(HK), FHKCPsych, FHKAM(Psychiatry)
Specialist in Psychiatry
Clinical Assistant Professor, Department of Psychiatry, The University of Hong Kong
Honorary Associate Consultant, Department of Psychiatry, Queen Mary Hospital

Mr Tommy KH FONG
MPsyMed, BSc (Psychology)
Department of Psychiatry, The University of Hong Kong

Dr May LAM
Vice President (Censor/ Education), the Hong Kong College of Psychiatrists

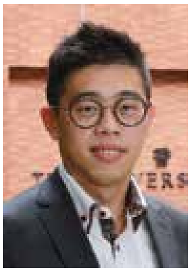
Dr Calvin PW CHENG

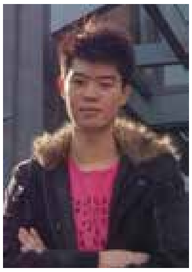
Mr Tommy KH FONG

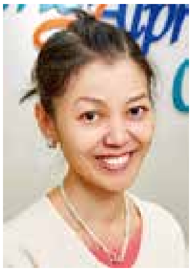
Dr May LAM

This article has been selected by the Editorial Board of the Hong Kong Medical Diary for participants in the CME programme of the Medical Council of Hong Kong (MCHK) to complete the following self-assessment questions in order to be awarded 1 CME credit under the programme upon returning the completed answer sheet to the Federation Secretariat on or before 31 May 2022.

Mental health problems remain a growing concern for adolescents in Hong Kong, with the overall prevalence being around 16.5% in 2008.[1] Hospital Authority figures also demonstrated that the caseload of the Child and Adolescent Psychiatric Team has increased by over 50%, from 18,900 cases in 2011-12 to 28,800 cases in 2015-16. Although social workers and health professionals from non-governmental organisations (NGOs) play a mediating role in assisting children and adolescents in facing stressors, parents play a more essential role in support of their children's mental health. This is important, as child and adolescent mental health is a core component of overall health. Having a good start in life and having parents educated about mental health can help children and adolescents develop lifelong resilience to adversity and can further maximise their individual potentials.[2] Parenting style and skills therefore play a critical factor in the child's development. Good parenting can have wide-ranging benefits for the child, from better cognitive and psychosocial development to protection from disease and mortality, resulting in better child health and development, especially for the neediest populations.[3] Furthermore, the impact of parenting on child development[4] includes: good mental and physical health, healthy lifestyles, the ability to confidently engage with others, and the development of self-esteem and a positive sense of self.

Unfortunately, family relationships have increasingly become a concern in Hong Kong. In a poll done by the Hong Kong Institute of Family Education in 2019, 22.4% of 509 interviewed parents of kindergarten, primary and secondary children reported to be under a lot of pressure, with 71% respondents rated within three to five on a 5-point Likert scale.[5] The main causes of stress are related to their children's emotions and academics. Recent societal events in Hong Kong have also further perpetuated the parents' perceived stress. In a recent survey, 80% of 515 parents interviewed reported that over 80% of them struggled in taking care of their children, as a result of school closures during the coronavirus epidemic.[6] In particular, parents who were required to work from home faced the highest levels of stress. Overall, communication between parents and school-aged children seemed to be lacking in the Hong Kong norm. There is a need to establish frequent and efficient parent-child communication so as to maintain a healthy family relationship. Furthermore, children and adolescents have different physical, social, emotional, and mental health needs at different stages of development. Evidence-based and tailor-made Parent Education in Mental Health (PEMH) is therefore critical to meet the needs of children and adolescents at different developmental stages, and to improve communication between parents and children.

In order to enhance parenting skills, alleviate parental stress, and improve parent-child communication, a two-year local PEMH programme in 2020 was conducted in Hong Kong, thanks to collaboration from psychiatrists, NGOs and six local primary schools. The objectives of this programme are (1) to let parents understand the emotional and social development of children and adolescents; (2) to strengthen the emotional bonding between parent and child; (3) to increase positive parental involvement in their children's education, behaviour and well-being; and (4) to learn about stress management skills which may reduce the stress of the parents. This programme was divided into three levels. Level one is for all parents (universal programme) including education talks and workshops (Details as shown in Fig. 1). Level two is an intermediate level programme, and is provided to those who are interested in further strengthening their parenting skills. The format would be mainly small-group workshops focused on specific topics. Level three is geared towards complex cases, adopting a one-to-one case management approach. The effectiveness of the programme would be evaluated through pre- and post-assessments on parents involved in this programme. Since the study is still on-going, the current analysis will only be focused on the interim results from the first academic year (i.e. 2020-2021) in order to assess the early effectiveness and feasibility of the programme.

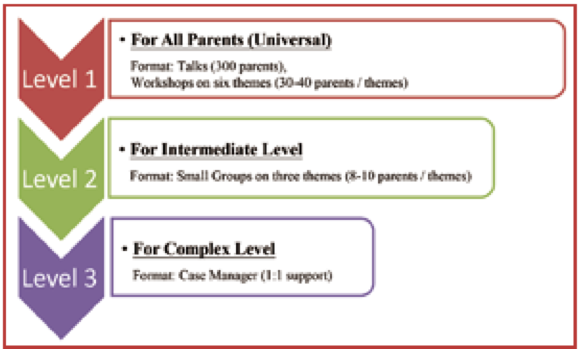

Fig. 1. Multi-Level Targets of Parent Education in Mental Health (PEMH) (Personal collection)

METHODS

Study Design and Participants

Parents from the dedicated local primary schools were recruited for the evaluation study. All parents who joined the talks and workshops from PEMH programme were instructed to complete baseline and follow-up questionnaires in Traditional Chinese via an online survey platform Qualtrics. Follow-up responses would be collected within the end of each academic year (i.e. 2021 July and 2022 July). Parents who have not participated in the PEMH programme within the academic year were also advised to take part in the follow-up survey for comparison. This study was approved by the Institutional Review Board of The University of Hong Kong/Hospital Authority, Hong Kong West Cluster (HKU/HA HKW IRB) (reference no.: UW 20-472).

Outcome Measures

Demographics

The parents' basic demographics, including age, gender, years of education, place of birth, religion, marital status, number of children, occupation, household income, and mental health were collected. In addition, their children's mental health, school class and academic performance were collected upon study entry.

Parenting stress

The stress levels of parents were examined by rating sentences representing positive parenthood themes and negative components through the 18-item Parental Stress Scale (PSS-18).[7] The PSS-18 used a 5-point Likert scale ranging from 1 (strongly disagree) to 5 (strongly agree), with higher scores indicating greater parental stress. The Chinese validated version of the scale has shown high reliability and consistency.[8,9]

Parenting style

Parenting styles were assessed by the Parenting Styles and Dimensions Questionnaire (PSDQ).[10,11] This shorter reworking version of the Parenting Practices Questionnaire adopted a 5-point Likert scale anchored by 1 (never) to 5 (always), with 32 items measuring the degree of authoritative, authoritarian, and permissive parenting styles. Larger numbers of index scores indicated a broader use of the corresponding parenting practices. The scores could be further manipulated into seven sub-categories (permissive; physical coercion; non-reasoning/punitive; verbal hostility; warmth and support; autonomy granting; regulation). Good reliability and validity have been shown for the PSDQ Chinese version.[12]

Self-Efficacy

The 10-item General Self-Efficacy Scale (GSE) was adopted to assess if parents were confident in confronting their child.[13] The self-reporting 4-point Likert scale ranged from 1 (not at all true) to 4 (exactly true), with higher scores indicating greater self-efficacy. The Chinese validated version of the GSE has shown great reliability and consistency.[14]

Children's attributes

The 25-item Strengths and Difficulties Questionnaire (SDQ) was used to identify potential positive or negative psychological attributes demonstrated by children.[15] Parents were required to identify if the listed items applied to their children by answering "not true", "somewhat true", and "certainly true". The scores could be sub-categorised into 5 classes: emotional symptoms, peer relationship problem, conduct problem, hyperactivity/ inattention, and prosocial behaviour; the overall difficulty scores were calculated by summarising the item scores of the first four classes excluding prosocial behaviour. The Chinese version of the scale has shown great reliability and validity.[16]

Data analysis

All analyses were performed using IBM SPSS Statistics (version 26) software. Descriptive statistics was used to observe participants' sociodemographic characteristics. Since all participating parents were off-labelled, all the differences between pre- and post-scores including stress, self-efficacy, parenting styles, and children attributes were determined by two-tailed between-group independent sample t-tests with a significance level set at $p < 0.05$. The follow-up assessment scores were also compared with those who have not participated in the PEMH programme within this academic year using between-group independent sample t-tests. Missing data for the outcome measures were handled by multiple imputations.[17]

RESULTS

By the end of the 2020-2021 academic year, a total of 2,246 parents across the six dedicated primary schools have participated in the PEMH programme. Among those, 1,771 of them completed the baseline questionnaire, resulting in a baseline response rate of 78.9%. After excluding duplicate entries and incomplete responses, a total of 1,393 (78.7%) valid responses were included in the final analyses. Table 1 describes the sociodemographic characteristics of the participants. Respondents were predominantly female (n = 1169, 84.4%). Nearly 90% of parents were aged between 31 and 50 years, with a mean age of 40.06 years. One-third (33.2%) have obtained a Bachelor's degree or above.

More than one-third of participants (37.4%) reported a monthly household income of less than HK$20,000, with 36.5% having a full-time work. About half (51.7%) of the sample had two children, yet 7.8% of overall respondents were divorced or separated, and 6.1% were not living with a partner or family. The mean age of children was 8.24 years, with slightly more reported males (51.5%). Regarding the health profile, 5.4% of the participants had a personal history of mental disorders, and 2.0% reported that their children had mental disorders.

Table 1. Sociodemographic characteristics of parents in PEMH programme (Unpublished data from PEMH programme)

	Overall (n = 1393)	
	n	%
Gender		
Male	216	15.6
Female	1169	84.4
Age		
18 – 30	37	2.8
31 – 40	638	48.5
41 – 50	572	43.4
51 or above	70	5.3
Marital status		
Married/ In relationship	1,226	92.2
Divorced/ Separated/ Widowed	104	7.8
Number of child(ren)		
1	481	38.4
2	648	51.7
3	110	8.8
4 or more	14	1.1
Education level		
Elementary or below	67	4.9
High School/ College	852	61.9
University or higher	457	33.2
Housing		
Private	616	46.4
Home Ownership Scheme	121	9.1
Public	392	29.5
Others	198	14.9
Living status		
Living with Partner	623	47.1
Living with family	619	46.8
Living alone/ Others	81	6.1
Number of persons living together		
2 persons	37	3.0
3 persons	304	24.5
4 persons	481	38.7
5 persons or more	419	33.8
Occupation status		
Full-time	485	36.5
Part-time	113	8.5
Homemaker/ Unemployed/ Retired	731	55.0
Monthly household income (HK$)		
< 20,000	476	37.4
20,000 – 39,999	336	26.4
40,000 – 59,999	127	10.0
≥ 60,000	334	26.2
Sufficient income for daily needs		
Yes	909	70.5
No	381	29.5
Family history on mental disorders		
Yes	46	3.6
No	1247	96.4
Personal history on mental disorders		
Yes	70	5.4
No	1,232	94.6
Child's gender		
Male	645	51.5
Female	608	48.5
Child's age		
5 or below	28	2.3
6 – 8	717	59.6
9 – 11	422	35.0
12 or above	37	3.1
Child's history on mental disorders		
Yes	25	2.0
No	1,235	98.0

Note. n = total number of participants. Only valid responses were included.

For the interim follow-up, an overall of 805 individual responses were collected. Of which, 601 survey responses were obtained from parents who participated in the PEMH programme, with 487 (81.0%) completed questionnaires included in post analysis. Table 2 presents the results of an independent sample t-tests on the PSS, GSE, PSDQ, and SDQ scale scores. Parents expressed significantly lower parental stress as evaluated by PSS after participating in the PEMH programme ($t = -2.00$, $p = 0.046$). Significant changes in parenting dimensions and styles were observed, with fewer parents adapting the permissive ($t = -2.66$, $p = 0.008$) and authoritarian ($t = -2.90$, $p = 0.004$) parenting styles as measured by PSDQ. In addition, parents demonstrated significantly fewer non-reasoning ($p < 0.05$) and verbal hostility ($p < 0.001$) parenting styles after a year of PEMH participation. An increasing trend of conforming to autonomy granting (from 3.73 to 3.79) under authoritative parenting was also noted. Decreases in SDQ total difficulties scores (mean difference = 0.32) and fewer hyperactivity problems (mean difference = 0.24) were measured, although the differences were not statistically significant. There was no significant difference on general self-efficacy scores.

Another 224 valid survey responses were received from parents who had not participated in the PEMH programme in this academic year before. Table 3 shows the comparison of the psychometric assessment scores between participants who participated in PEMH and those who did not. Parents who have not participated in the PEMH programme before reported significantly fewer emotional symptoms from their children as compared to those who have participated ($t = 2.22$, $p = 0.026$). Trends of having more competent self-efficacy (GSE total scores: 23.66 vs 22.93) and fewer hyperactivity problems in children (SDQ hyperactivity scores: 4.34 vs 4.66) were found in parents participated in PEMH, but such difference have not been shown to be statistically significant.

DISCUSSION

During the first-year establishment of the PEMH programme, parents have already reflected positive outcomes in parenting their children. Our study provides early empirical evidence that parenting education in mental health programme pioneered in Hong Kong local primary schools helps to alleviate parental stress and shift parenting dimensions. Parents who participated in the PEMH programme demonstrated less permissive parenting styles and adopted a more interactive parenting approach, which might have contributed to the reduced psychosocial difficulties shown in children, in particular for hyperactivity or inattention. This study suggested that PEMH is effective in enhancing positive parental involvement, managing stress, and strengthening the emotional bond between parents and children so as to facilitate a better family relationship.

One of the major observations seen would be the transient alternations of the parenting styles, which is undeniably the main aspiration through exercising the PEMH programme. Despite a lack of conclusive evidence that indicates a switch from permissive and authoritarian parenting towards authoritative

Certificate Course for Doctors, Radiographers, and Nurses who are working in the Emergency Department, Family Medicine, or women's clinic

• Course No. C379 **• CME/CNE Course**

Certificate Course on

Medical Ultrasound 2022

(Video Lectures)

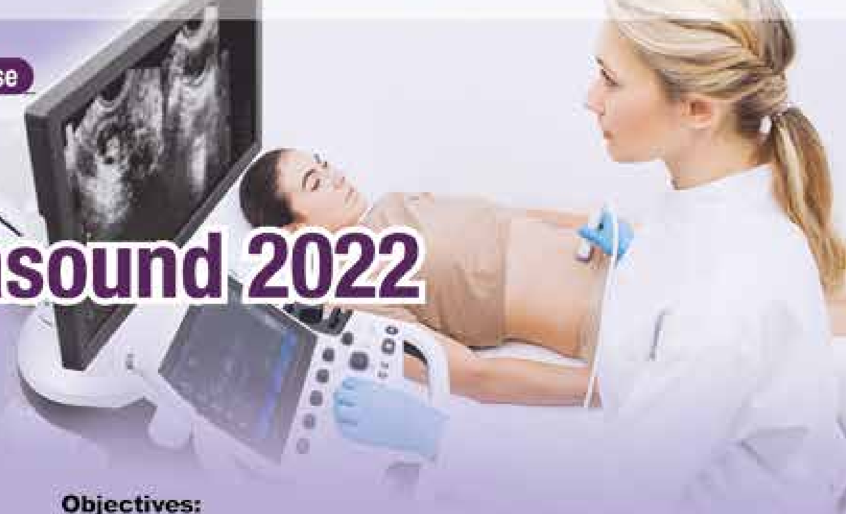

Jointly organised by

The Federation of Medical Societies of Hong Kong

HKSUM Hong Kong Society for Ultrasound in Medicine

Hong Kong Society for Ultrasound in Medicine

Objectives:

To provide up-to-date knowledge on point-of-care (POC) ultrasound to diagnose common and important medical problems seen in the acute, ward, and clinic settings. Ultrasonography in women's health including breast, gynaecology, and obstetrics will be covered. The course will be suitable for doctors, radiographers, and nurses who are working in the emergency department, family medicine, or women's clinic.

Date	Topics	Speakers
5 July 2022	Principle of ultrasound and basic techniques (for beginners)	Dr CHONG Yee-ming, Alan<br>Resident<br>Radiology, Queen Mary Hospital
12 July 2022	Point-of-care ultrasound for emergency (for Accident & Emergency)	Dr. CHAN Lok-hei, Derek<br>Resident<br>Radiology, Queen Mary Hospital
19 July 2022	Point-of-care ultrasound for general (for Family Medicine)	Dr TANG Hok-him, Wisely<br>Resident<br>Radiology, Queen Mary Hospital
26 July 2022	Mammogram + breast ultrasound	Dr. LAM Poy-wing, Tina<br>Chief of Service<br>Radiology, Queen Mary Hospital
2 August 2022	Gynaecological ultrasound	Dr. Grace HO<br>Consultant<br>Radiology, Queen Mary Hospital
9 August 2022	New ultrasound technology and safety (including regulations & control)	Dr. LEUNG Kwok-yin<br>President<br>Hong Kong Society for Ultrasound in Medicine

Date : 5, 12, 19, 26 July & 2, 9 August 2022 (Every Tuesday)
Duration of session: 1.5 hours (6 sessions)
Time : 7:00 pm – 8:30 pm
Course Feature: Video lectures (with Q&A platform for participants to post the questions)
Quiz for doctors: DOCTORS are required to complete a quiz after the completion of each lecture
Language Media : Cantonese (Supplemented with English)
Course Fee : HK$1,000
Certificate : Awarded to participants with a minimum attendance of 70% (4 out of 6 sessions)
Deadline : 27 June 2022
Enquiry : Tel.: 2527 8898 Fax : 2865 0345 Email : vienna.lam@fmshk.org

CME / CNE/ CPD (Radiographers) Accreditation in application
Online Application from website: http://www.fmshk.org

Table 2. Independent sample t-tests comparing baseline and follow-up assessment scores (Unpublished data from PEMH programme)

	Baseline		Follow-up			
	M	SD	M	SD	t	p-Value
Parental Stress Scale (PSS-18)	52.07	3.53	51.70	3.62	– 2.00	0.046*
General Self-Efficacy Scale (GSE-10)	23.42	5.16	23.66	5.17	0.86	0.392
Parenting Styles and Dimensions Questionnaire (PSDQ)						
Permissive	2.58	0.56	2.50	0.53	– 2.66	0.008**
Authoritarian	2.23	0.54	2.14	0.50	– 2.90	0.004**
Physical coercion	1.88	0.62	1.83	0.57	– 1.63	0.104
Non-reasoning/ Punitive	2.03	0.62	1.97	0.57	– 2.03	0.043*
Verbal hostility	2.76	0.68	2.64	0.64	– 3.50	< 0.001***
Authoritative	3.87	0.52	3.89	0.52	0.85	0.394
Warmth and support	4.02	0.58	4.05	0.57	0.96	0.337
Autonomy granting	3.73	0.58	3.79	0.59	1.86	0.063
Regulation	3.86	0.60	3.84	0.60	– 0.54	0.586
Strengths & Difficulties Questionnaires (SDQ)						
Emotional symptoms	2.77	2.06	2.66	1.99	– 0.98	0.328
Peer relationship problems	2.70	1.70	2.76	1.65	0.62	0.539
Conduct problems	2.34	1.49	2.32	1.43	– 0.31	0.760
Hyperactivity/ inattention	4.58	2.37	4.34	2.30	– 1.90	0.057
Prosocial behaviour	6.74	1.91	6.78	1.90	0.40	0.690
Total difficulties	12.40	5.41	12.08	5.20	– 1.12	0.264

Note. * $p < 0.05$, ** $p < 0.01$, *** $p < 0.001$. M = mean; SD = standard deviation. t-tests (t) comparing baseline and follow-up data.

Table 3. Comparison of assessment scores between PEMH participated and not participated samples (Unpublished data from PEMH programme)

	Participated		Not Participated			
	M	SD	M	SD	t	p-Value
Parental Stress Scale (PSS-18)	51.70	3.62	51.52	3.95	0.58	0.566
General Self-Efficacy Scale (GSE-10)	23.66	5.17	22.93	5.65	1.70	0.090
Parenting Styles and Dimensions Questionnaire (PSDQ)						
Permissive	2.50	0.53	2.52	0.54	– 0.40	0.692
Authoritarian	2.14	0.50	2.16	0.59	– 0.40	0.688
Physical coercion	1.83	0.57	1.79	0.67	0.75	0.456
Non-reasoning/ Punitive	1.97	0.57	2.04	0.66	– 1.40	0.163
Verbal hostility	2.64	0.64	2.66	0.73	– 0.37	0.710
Authoritative	3.89	0.52	3.88	0.49	0.30	0.763
Warmth and support	4.05	0.57	4.04	0.55	0.17	0.868
Autonomy granting	3.79	0.59	3.73	0.56	1.18	0.239
Regulation	3.84	0.60	3.87	0.54	– 0.55	0.585
Strengths & Difficulties Questionnaires (SDQ)						
Emotional symptoms	2.66	1.99	2.30	1.87	2.22	0.026*
Peer relationship problems	2.76	1.65	2.91	1.70	– 1.15	0.249
Conduct problems	2.32	1.43	2.19	1.52	1.10	0.271
Hyperactivity/ inattention	4.34	2.30	4.66	2.43	– 1.70	0.089
Prosocial behaviour	6.78	1.90	6.86	2.04	– 0.50	0.620
Total difficulties	12.08	5.20	12.07	5.55	0.03	0.973

Note. * $p < 0.05$, ** $p < 0.01$, *** $p < 0.001$. M = mean; SD = standard deviation. t-tests (t) omparing participated PEMH and not participated PEMH sample.

parenting, the significant diminution of permissive and authoritarian parenting styles could abundantly influence the psychosocial development of the children. It has been revealed that paternal authoritarian parenting styles can contribute to poorer mental health in adulthood, including symptomatic problems, the risk to self and others, life functioning, and psychological well-being.[18] Ultimately, with the positive trend of switching in parenting styles insight, we could expect fewer psychological problems during children's later developmental stages.

The unprecedented COVID-19 outbreak has precipitated extensive changes in school education locally, such as switching to distance-learning or even class suspension. It is not a surprise that children receiving distance learning at home has further aggravated the long accumulated stress in parents, as parents have to juggle between monitoring their children's schoolwork while managing their own work from home at the same time.[19] Indeed, the initiative of the PEMH programme has not accounted for the influence of the pandemic. As most of the PEMH talks and workshops were conducted online, parents might probably be less engaged in discussions in the online environment. Still, the significant reduction in parental stress through PEMH further exhibited the imperative proposition of parent education in improving family relationships, especially with increased family time during the pandemic era.

Accordingly, the interim results of our study could be one of the point prevalence in revealing the pandemic repercussions on family relationships. The mean total scores of PSS in both baseline and follow-up in this study were higher than the scores from parents of children aged 6 - 12 emanated from another local study (n = 17,029; PSS mean total: 49.72) conducted within the first outbreak period (March 2020).[20] Similar phenomenon was also observed in the total difficulties scores measured by SDQ (baseline: 12.40; follow-up: 12.08 vs 11.59).20 It is undoubtedly alarming that parents are observed to have higher parental stress, and children have poorer psychological attributes since the start of the prevailing pandemic. The establishment of the PEMH programme therefore offered an opportunity for parents to reflect on their parenting inadequacies, and at the same time, formulate a comfortable platform for outreach services that help needy parents to deal with family issues.

In order to evaluate the effectiveness of the PEMH programme, our study compared the parents who have and have not participated in the PEMH programme. Parents were still in concern of their children with high emotional symptoms regardless of one-year PEMH participation. The continuation of children suffering emotional problems might be related to the disrupted normal social activities, along with the increased usage of electronic devices and inadequate sleep or exercise during the coronavirus epidemic.[19] Despite so, it was observed that participation in the PEMH programme yielded more positive outcomes: (1) parents demonstrated improved self-efficacy, and (2) children exhibited fewer negative attributes. It is expected that notable improvements could accrue from further parenting education, as an early introduction of parent education can help prepare parents to better handle potential issues that their children will face at different developmental stages, which may, as a result, improve the mental health of children and adolescents. This is important as evidence has suggested that as much as 50% of mental illnesses developed in adulthood could be prevented in children with better mental health.[21]

The results from this study should be interpreted with caution in accordance with the following limitations. Due to the overall data collection via an electronic platform, a lower attribution rate was acknowledged without direct assessment and monitoring from programme contributors. Furthermore, possible reporting bias could be resulted in view of the epidemic impact, but not solely on the PEMH programme. Owing to the off-label and non-compulsory nature of the study, the strength of the statistical analyses was limited by the imbalance of survey responses collected between baseline and follow-up. Furthermore, this study only included the interim results from first year introduction of PEMH programme. The finalised findings after two-year completion might have conflicting conclusions from the current outcomes.

In conclusion, our study consolidates the importance of parent education in mental health. PEMH is showing monumental potential in strengthening the emotional bonding between parent and child through increasing positive parental involvement in their children's education, behaviour and well-being, and hence alleviating parental stress and maintaining healthy family relationships. The prevalence of high parental stress and psychological problems in children reflected in the findings highlight the importance of resolving the negligence of boiling parenting pressure. This study could be viewed as the cornerstone of parent education with respect to the broader imputation of PEMH on parents and educators in the pre-schooling stage.

References

1. Leung, P.W., et al., Prevalence of DSM-IV disorders in Chinese adolescents and the effects of an impairment criterion. European child & adolescent psychiatry, 2008. 17(7): p. 452-461.
2. Schaefer, E.S., Goals for parent and future-parent education: Research on parental beliefs and behavior. The Elementary School Journal, 1991. 91(3): p. 239-247.
3. World Health Organization, Bulletin of the World Health Organization: Bulletin de L'Organisation Mondiale de la Santé. 2006: World Health Organization.
4. Steinberg, L., et al., Impact of parenting practices on adolescent achievement: Authoritative parenting, school involvement, and encouragement to succeed. Child development, 1992. 63(5): p. 1266-1281.
5. Chiu, P. One-fifth of Hong Kong parents under too much strain at home, survey finds, leaving little time to ask children about their problems. 2019 [cited 2020 May 30]; Available from: https://www.scmp.com/news/hong-kong/education/article/3010844/one-fifth-hong-kong-parents-under-too-much-strain-home.
6. Low, Z. More than 80 per cent of Hong Kong's parents highly stressed as schools remain shut amid coronavirus epidemic, poll finds. 2020 [cited 2020 May 30]; Available from: https://www.scmp.com/news/hong-kong/education/article/3075839/more-80-cent-hong-kongs-parents-highly-stressed-schools.
7. Berry, J.O. and W.H. Jones, The parental stress scale: Initial psychometric evidence. Journal of Social and Personal Relationships, 1995. 12(3): p. 463-472.
8. Leung, C. and S.K.M. Tsang, The Chinese Parental Stress Scale: Psychometric Evidence Using Rasch Modeling on Clinical and Nonclinical Samples. Journal of Personality Assessment, 2010. 92(1): p. 26-34.
9. Cheung, S.-K., Psychometric properties of the Chinese version of the Parental Stress Scale. Psychologia: An International Journal of Psychology in the Orient, 2000.
10. Robinson, C.C., et al., The parenting styles and dimensions questionnaire (PSDQ). Handbook of family measurement techniques, 2001. 3: p. 319-321.
11. Robinson, C.C., et al., Authoritative, authoritarian, and permissive parenting practices: Development of a new measure. Psychological reports, 1995. 77(3): p. 819-830.
12. Fu, Y., et al., Can parenting styles and dimensions questionnaire (PSDQ) be used in China? Psychology, 2013. 4(06): p. 535.
13. Schwarzer, R., et al., Measures in health psychology: A user's portfolio. Causal and control beliefs. Windsor, UK: Nfer-Nelson, 1995: p. 35-7.
14. Zhang, J.X. and R. Schwarzer, Measuring optimistic self-beliefs: A Chinese adaptation of the General Self-Efficacy Scale. Psychologia: An International Journal of Psychology in the Orient, 1995.
15. Goodman, R., The Strengths and Difficulties Questionnaire: a research note. Journal of child psychology and psychiatry, 1997. 38(5): p. 581-586.
16. Lai, K.Y., et al., Validation of the Chinese version of the strengths and difficulties questionnaire in Hong Kong. Social psychiatry and psychiatric epidemiology, 2010. 45(12): p. 1179-1186.
17. Schlomer, G.L., S. Bauman, and N.A. Card, Best practices for missing data management in counseling psychology. Journal of Counseling psychology, 2010. 57(1): p. 1.
18. Uji, M., et al., The impact of authoritative, authoritarian, and permissive parenting styles on children's later mental health in Japan: Focusing on parent and child gender. Journal of child and family studies, 2014. 23(2): p. 293-302.
19. Lau, E.Y.H. and K. Lee, Parents' views on young children's distance learning and screen time during COVID-19 class suspension in Hong Kong. Early Education and Development, 2021. 32(6): p. 863-880.
20. Tso, W.W., et al., Vulnerability and resilience in children during the COVID-19 pandemic. European child & adolescent psychiatry, 2020: p. 1-16.
21. Kessler, R.C., et al., Lifetime prevalence and age-of-onset distributions of DSM-IV disorders in the National Comorbidity Survey Replication. Archives of general psychiatry, 2005. 62(6): p. 593-602.

MCHK CME Programme Self-assessment Questions

Please read the article entitled "Exploration of the New Parent Education in Mental Health Programme (PEMH) at Hong Kong Primary Schools - Interim Results" by Dr Calvin PW CHENG, Mr Tommy KH FONG and Dr May LAM and complete the following self-assessment questions. Participants in the MCHK CME Programme will be awarded CME credit under the Programme for returning completed answer sheets via fax (2865 0345) or by mail to the Federation Secretariat on or before 31 May 2022. Answers to questions will be provided in the next issue of The Hong Kong Medical Diary.

Questions 1-10: Please answer T (true) or F (false)

1. Mental health problems are not common among children and adolescents in Hong Kong.
2. Good parenting can help children develop better physical and mental health.
3. Parents who were required to work from home faced the highest levels of carer stress.
4. Children with better mental health could prevent as much as 50% of mental illnesses developed in adulthood.
5. The demand of psychiatric services has remained static in the recent 10 years.
6. The main causes of the parents' stress are related to their children's emotions and their academics.
7. The authoritative parenting style usually includes physical coercion and verbal hostility.
8. Poorer adulthood mental health could be contributed to by authoritarian parenting style.
9. Children suffered high emotional symptoms during the pandemic due to more conflicts with their parents.
10. Early parent education, for example, in the pre-schooling stage, is recommended in order to increase positive parental involvement and maintain healthy family relationships.

ANSWER SHEET FOR MAY 2022

Please return the completed answer sheet to the Federation Secretariat on or before 31 May 2022 for documentation. 1 CME point will be awarded for answering the MCHK CME programme (for non-specialists) self-assessment questions.

Exploration of the New Parent Education in Mental Health Programme (PEMH) at Hong Kong Primary Schools - Interim Results

Dr Calvin PW CHENG
MBBS(HK), FHKCPsych, FHKAM(Psychiatry)
Specialist in Psychiatry
Clinical Assistant Professor, Department of Psychiatry, The University of Hong Kong
Honorary Associate Consultant, Department of Psychiatry, Queen Mary Hospital

Mr Tommy KH FONG
MPsyMed, BSc (Psychology)
Department of Psychiatry, The University of Hong Kong

Dr May LAM
Vice President (Censor/ Education), the Hong Kong College of Psychiatrists

1 ☐ 2 ☐ 3 ☐ 4 ☐ 5 ☐ 6 ☐ 7 ☐ 8 ☐ 9 ☐ 10 ☐

Name (block letters):____________________ HKMA No.: ______________ CDSHK No.: ____________

HKID No.: __ __ - __ __ __ __ X X (X) HKDU No.: ______________ HKAM No.: ____________

Contact Tel No.:____________________ MCHK No. / DCHK No.: ______________(must fill in)

Answers to April 2022 Issue

Intra-arterial Thrombectomy for Acute Stroke Management

1. T **2.** T **3.** T **4.** T **5.** F **6.** F **7.** F **8.** T **9.** F **10.** F

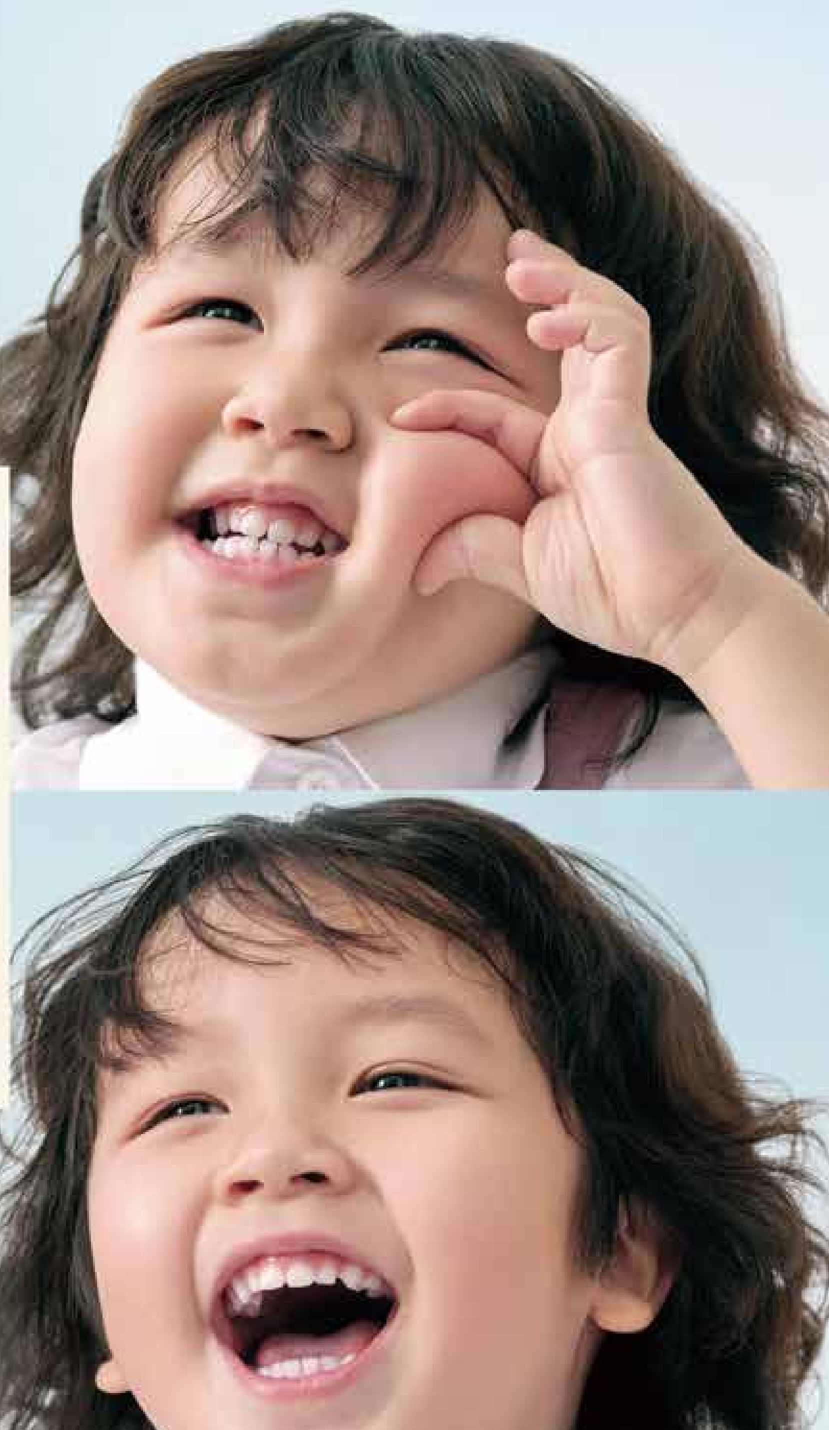

由外而內，
樂得純真。

Elidel®膚樂得®無類固醇抗濕疹乳霜

- 有效舒緩濕疹徵狀*，減低復發風險[1,2]
- 適合孩子敏感肌膚間歇性長期使用[1,2]
- 修復外在天然屏障，補充肌膚濕度[3,4]

消炎·補濕·止癢[2,4] 2歲或以上適用[1,2]

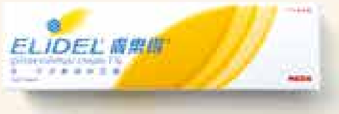

ELIDEL

[illegible]

參考資料：[illegible]

ELIDEL SUMMARY OF PRODUCT INFORMATION [illegible]

FULL PRESCRIBING INFORMATION IS AVAILABLE UPON REQUEST.

Mylan Pharmaceutical Hong Kong Ltd., a Viatris company
Suites 2401-07 & 12, 24/F., One Island East, 18 Westlands Road, Quarry Bay, Hong Kong. T 2290 7100 F 2873 0006 W www.viatris.com
©2022 VIATRIS — All Rights Reserved PP-ELI-HKG-0020 APR 2022

VIATRIS
暉致

Practice of Parent Education from Experience at the 3 Tier Levels: at Clinic, School and Community

Ms Wai-ping CHAN
Nurse Consultant (Psychiatry/Child and Adolescent), Queen Mary Hospital, Hong Kong West Cluster

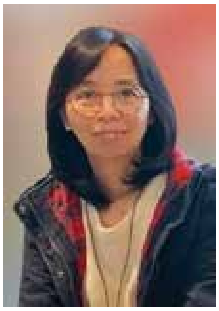
Ms Wai-ping CHAN

INTRODUCTION

Parent education is the provision of specific knowledge and child-rearing skills to parents and other caregivers, with the objective of enhancing a child's health and development.[1,2]

Parents are our children's first mentors. Parents play a vital role in children's development. Our parents have always been saying that they were not taught to be parents, and their parenting practices are passed on from generation to generation. As our society evolves and with new parenting challenges and struggles, parent education is of utmost importance.

In this article, it would be my pleasure to share my experience of the practice of parent education from different perspectives.

CHILD and ADOLESCENT PSYCHIATRIC SPECIALIST CLINIC (TERTIARY LEVEL)

The Mental Health Review Report[3] recommends the adoption of a three-tier stepped care service model to facilitate cross-sectoral and multi-disciplinary collaboration in the delivery of child and adolescent mental health services. At the tertiary level, intensive care and crisis intervention is required for those having complex and enduring problems.

The child and adolescent psychiatric service of Queen Mary Hospital first started in 1970. Our service was initially focused on neurodevelopmental disorders such as Attention Deficit Hyperactivity Disorder (ADHD), Autistic Spectrum Disorder (ASD), Tics, etc. The scope of our service has been gradually broadened since the 1980s to include the management of child abuse, disruptive behavioural disorders, emotional and mood disturbances, substance abuse, and suicidal behaviours. We provide comprehensive psychiatric services through a multi-disciplinary approach. Our programmes include clinical assessment, treatment, and training packages for patients with ADHD, ASD, Dyslexia, Tourette Syndrome, Eating Disorders, etc. All treatment programmes are evidenced- and manual-based, such as ADHD/ASD Pre-primary class, Homework Supervision Group, Parent Management Training, ADHD/ASD Heterosexual/Interpersonal Training Group, Reading and Handwriting Group, Habit Reversal Training, etc.

Parent education is an essential element in most treatment programmes. Establishing a good rapport with parents is our first step to engaging them. Parents and children are invited to attend our Nurse Assessment Clinic before the first medical appointment. Nurses would carry out comprehensive assessments with established assessment guidelines and provide relevant parenting advice and community resources to patients and their carers based on the clinical assessment. In order to enhance better continuity of care, our Nurse clinic provides timely support to parents and patients with complex needs in the specialist clinic. Even if the child was admitted to our in-patient ward, we provided carer support as needed as it is one of the important components of care.

Case sharing

John, six years old, diagnosed with ADHD and ASD, is being followed up at the Child and Adolescent Psychiatric Specialist Clinic at Queen Mary Hospital. He was reluctant to complete homework at home. His mother found it difficult to supervise him. Inconsistent parenting was noted. His mother felt distressed to handle the child, and she sometimes would resort to physical punishment. Hence, both the child and his mother were referred to our Day Hospital for attending the Executive Function Homework Supervision Workshop and Nurse Clinic for parent management training. There were three parent education sessions and one child session in the homework supervision workshop.

After completing the sessions, his mother could understand the relationship between ADHD and executive function deficits, and could thus understand the child's difficulties in homework. Besides, she could acknowledge the child's own emotions, reduce the child's stress on homework, and be able to enhance the child's executive function skills on homework. Hence, with parent education, the mother acquires the skill of stress management over homework and is empowered to enhance the child's executive function in homework and study. In addition, the mother became more confident in supervising the child's homework, as she had the chance to practise and apply the skills during the child session, with the therapist giving her instant feedback. In addition, his mother learned effective and positive parenting skills through the parent management training sessions. Consequentially, the child's behaviour and the mother-child relationship improved.

STUDENT MENTAL HEALTH SUPPORT SCHEME (SMHSS)[4] (SECONDARY LEVEL)

The scheme aligns with the Stepped Care Model, which facilitates cross-sectoral and multi-disciplinary

collaboration to provide Tier-2 intervention for students with mental health needs at school. The school-based platform comprises parents, teachers, educational psychologists, school social workers, and healthcare professionals. The team would review the progress of nominated students (both known and unknown cases to Hospital Authority (HA) Child and Adolescent psychiatric services), and adjust the intervention strategies and care plans if necessary.

Parent education plays a vital part in the scheme. The team members (psychiatric nurses and school social workers) would provide support to the family on parenting skills, parent-child activities, and relationships, and link up community-based social services for the family, to facilitate the implementation of the care plan for the student.

Case sharing

John, thirteen years old, Form two student, diagnosed with ADHD, having been followed up at the Child and Adolescent Psychiatric Specialist Clinic at Queen Mary Hospital, was referred to the scheme because he had frequent temper tantrums at school and poor drug compliance after promotion to secondary school. First, the multi-disciplinary team collected information and performed a preliminary assessment. It was noted that his grades had dropped and the teachers always scolded him for his inattentiveness and forgetfulness. His drug compliance was poor as he thought that he had already grown up and was reluctant to take the drug. His mother had high expectations of him and always used materialistic rewards to encourage him to study. However, there were many parent-child conflicts, and the mother felt distressed handling the child, especially during the Covid-19 period.

Following this understanding, the team formulated an integrated assessment and care plan which would increase the child's understanding and insight of his problems with ADHD, enhance his behaviour control at school, and monitor his mental condition, drug-taking pattern, medication side-effects, and drug compliance. Besides, SMHSS nurses discussed with his teacher to set an appropriate behavioural programme for him whereby his teachers would help monitor his behaviour in school. The educational psychologist helped perform an academic assessment, provide accommodation as needed, and encourage the child to apply effective learning strategies.

In addition, school social workers coach the mother's effective parenting skills to improve parents' understanding of child's problems, set realistic expectations, and thus improve the parent-child relationship.

COMMUNITY (PRIMARY LEVEL)

Parents play a significant role in early childhood development. Universal parent education programmes are conducted by various sectors in the community, such as health, social welfare, and education. Apart from those in need, the programmes are beneficial to other families in general.[5] Positive Parenting Programme (Triple P) is one of the most successful evidence-based parenting programmes in the world, based on more than 35 years of research.[6] Parents are provided with simple, practical strategies to build strong and healthy family relationships. The programme was piloted from 2001 to 2002 in Hong Kong. Based on the results, it was found effective in decreasing child behavioural problems and dysfunctional styles and improving parental competency, as well as enhancing marital relationships.[7] Hence, it has been introduced to all Maternal and Child Health Centres since then.

Recently, in order to expand the coverage of community-based parent education, there have been two pilot community collaborative programmes - Parent Education in Mental Health (PEMH) and New Service Protocol for Attention Deficit Hyperactivity Disorder (ADHD+). PEMH offers evidence-based and tailor-made talks or workshops according to different developmental stages of primary school students, and a continuous six-year curriculum. These talks or workshops target "hidden" parents, parents of students with Special Education Needs, and grassroots parents as well. ADHD+ offers manualised treatment packages for mild and moderate ADHD children, with or without comorbidities, not receiving HA or private psychiatric care, in community-based service settings. During the on-site support sessions, we noticed that all parents were actively involved and engaged in group activities of parent management training. We received lots of positive feedback, such as "Parent-child relationship has improved", "I have learned how to praise my child and my child feels good", "I feel relaxed", "No more homework battles", etc.

CONCLUSION

Almost everyone benefits from parent education.[8] Wilder Research conducted a literature review on parent education programmes in 2016. It revealed that the key benefits of parent education include: improving parental empowerment and competency, increasing positive parenting practices, increasing social connections, improving child behaviour, improving parent-child interactions, improving parental mental health and well-being, and decreasing the use of corporal punishment and risk of child abuse.[9] In Hong Kong, there are a variety of parent education programmes, all with different formats, theoretical assumptions, and clients. These programmes are provided by different sectors on a piecemeal basis. As such, the government should take a leadership role in integrating and developing theory-driven and evidence-based programmes that will adapt to changing social contexts and parental expectations.

References

1. Croake, J. W., & Glover, K. E. (1977). A History and Evaluation of Parent Education. The Family Coordinator, 26(2), 151–158. https://doi.org/10.2307/583363
2. Zepeda, Marlene & Varela, Frances & Morales, Alex. (2004). Promoting Positive Parenting Practices through Parenting Education. Building State Early Childhood Comprehensive Systems Series, Number 13. UCLA Center for Healthier Children, Families and Communities.
3. Mental Health Report, April 2017
4. Operations Guideline on Student Mental Health Support Scheme, March 2020
5. Lindsay, G., Totsika, V. The effectiveness of universal parenting programmes: the CANparent trial. BMC Psychol 5, 35 (2017).
6. Triple P takes the guesswork out of parenting. Triple P Implementation | Official Corporate site. (n.d.). https://www.triplep.net/glo-en/home/
7. https://www.fhs.gov.hk/english/archive/files/reports/Triple_full.pdf
8. Parenting classes benefit all, especially lower-income families. (2016, December 5). Life at OSU.
9. The Benefits of Parenting Education A Review of the Literature for the Wilder Parent Education Center Key benefits of Parenting Education. (2016).

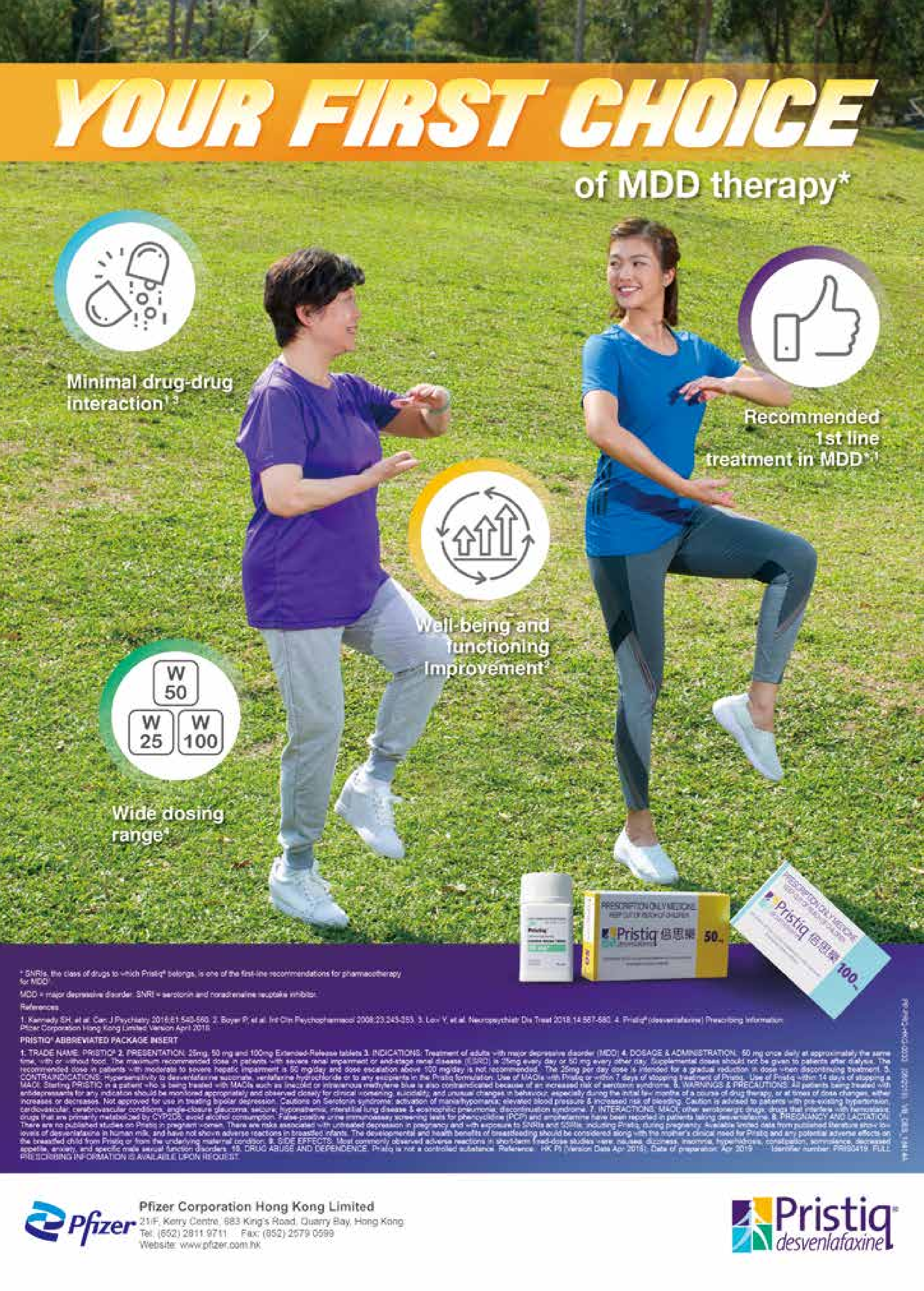

YOUR FIRST CHOICE
of MDD therapy*
Minimal drug-drug interaction[1-3]
Recommended 1st line treatment in MDD*[1]
Well-being and functioning improvement[2]
W 50
W 25
W 100
Wide dosing range[4]
PRESCRIPTION ONLY MEDICINE
KEEP OUT OF REACH OF CHILDREN
Pristiq 倍思樂
50mg
100mg
* SNRIs, the class of drugs to which Pristiq® belongs, is one of the first-line recommendations for pharmacotherapy for MDD[1]
MDD = major depressive disorder; SNRI = serotonin and noradrenaline reuptake inhibitor.
References
1. Kennedy SH, et al. Can J Psychiatry 2016;61:540-560. 2. Boyer P, et al. Int Clin Psychopharmacol 2008;23:243-253. 3. Low Y, et al. Neuropsychiatr Dis Treat 2018;14:567-580. 4. Pristiq® (desvenlafaxine) Prescribing Information. Pfizer Corporation Hong Kong Limited Version April 2018.
PRISTIQ® ABBREVIATED PACKAGE INSERT
1. TRADE NAME: PRISTIQ® 2. PRESENTATION: 25mg, 50 mg and 100mg Extended-Release tablets 3. INDICATIONS: Treatment of adults with major depressive disorder (MDD) 4. DOSAGE & ADMINISTRATION: 50 mg once daily at approximately the same time, with or without food. The maximum recommended dose in patients with severe renal impairment or end-stage renal disease (ESRD) is 25mg every day or 50 mg every other day. Supplemental doses should not be given to patients after dialysis. The recommended dose in patients with moderate to severe hepatic impairment is 50 mg/day and dose escalation above 100 mg/day is not recommended. The 25mg per day dose is intended for a gradual reduction in dose when discontinuing treatment. 5. CONTRAINDICATIONS: Hypersensitivity to desvenlafaxine succinate, venlafaxine hydrochloride or to any excipients in the Pristiq formulation. Use of MAOIs with Pristiq or within 7 days of stopping treatment of Pristiq. Use of Pristiq within 14 days of stopping a MAOI. Starting PRISTIQ in a patient who is being treated with MAOIs such as linezolid or intravenous methylene blue is also contraindicated because of an increased risk of serotonin syndrome. 6. WARNINGS & PRECAUTIONS: All patients being treated with antidepressants for any indication should be monitored appropriately and observed closely for clinical worsening, suicidality, and unusual changes in behaviour, especially during the initial few months of a course of drug therapy, or at times of dose changes, either increases or decreases. Not approved for use in treating bipolar depression. Cautions on Serotonin syndrome; activation of mania/hypomania; elevated blood pressure & increased risk of bleeding. Caution is advised to patients with pre-existing hypertension, cardiovascular, cerebrovascular conditions, angle-closure glaucoma, seizure, hyponatremia, interstitial lung disease & eosinophilic pneumonia; discontinuation syndrome. 7. INTERACTIONS: MAOI, other serotonergic drugs, drugs that interfere with hemostasis, drugs that are primarily metabolized by CYP2D6, avoid alcohol consumption. False-positive urine immunoassay screening tests for phencyclidine (PCP) and amphetamine have been reported in patients taking desvenlafaxine. 8. PREGNANCY AND LACTATION: There are no published studies on Pristiq in pregnant women. There are risks associated with untreated depression in pregnancy and with exposure to SNRIs and SSRIs, including Pristiq, during pregnancy. Available limited data from published literature show low levels of desvenlafaxine in human milk, and have not shown adverse reactions in breastfed infants. The developmental and health benefits of breastfeeding should be considered along with the mother's clinical need for Pristiq and any potential adverse effects on the breastfed child from Pristiq or from the underlying maternal condition. 9. SIDE EFFECTS: Most commonly observed adverse reactions in short-term fixed-dose studies were: nausea, dizziness, insomnia, hyperhidrosis, constipation, somnolence, decreased appetite, anxiety, and specific male sexual function disorders. 10. DRUG ABUSE AND DEPENDENCE: Pristiq is not a controlled substance. Reference: HK PI (Version Date Apr 2018); Date of preparation: Apr 2019 Identifier number: PRIS0419. FULL PRESCRIBING INFORMATION IS AVAILABLE UPON REQUEST.
Pfizer Corporation Hong Kong Limited
21/F, Kerry Centre, 683 King's Road, Quarry Bay, Hong Kong
Tel: (852) 2811 9711 Fax: (852) 2579 0599
Website: www.pfizer.com.hk
Pristiq®
desvenlafaxine

The Happy Kids Charter - Practical Tips to Promote Happy and Holistic Development in Children

Dr Krystal CK LEE
MBBS (HKU), FHKCCM (Public Health Medicine), FHKCPsych, FHKAM (Community Medicine), FHKAM (Psychiatry)

Specialist in Psychiatry
Associate Consultant, Department of Psychiatry, Queen Mary Hospital

Dr Phyllis KL CHAN
MBBS(HK), FRC Psych (UK), FHKC Psych, FHKAM (Psychiatry)

Consultant Psychiatrist (partime), Queen Mary Hospital & Kwai Chung Hospital
Honorary Clinical Associate Professor, Department of Psychiatry,
LKS Faculty of Medicine, The University of Hong Kong
Vice-President (General Affairs), The Hong Kong College of Psychiatrists

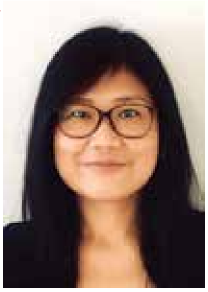
Dr Krystal CK LEE

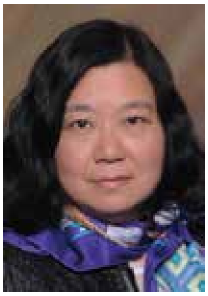
Dr Phyllis KL CHAN

PREAMBLE

A few years back, a spate of students' suicide with widespread media coverage arose the public concern about the mental health of children in Hong Kong. A committee on the Prevention of Student Suicides was set up to investigate the tragic deaths of 71 students in Hong Kong between 2013 and 2016. While there were multiple contributing factors, family relationships and academic stress were the important ones identified.[1] From the phenomenon of 'tiger mom' to the popularity of the phrase 'winning at the starting line', it is apparent that some local parents tend to prioritise achievement over the holistic development and mental well-being of their children. While for most children, an appropriate level of stress can nurture them to respond by setting goals and working hard effectively, long-term or inappropriately high levels of stress may lead to unhealthy brain development, mental and physical illness not only in childhood but in adulthood.[2] It is therefore imperative to promote and educate parents on ways of maintaining good parent-child relationships and managing academic stress.

THE HAPPY KIDS CHARTER

In 2015, a panel of local experts and community partners met, reviewed the evidence and formulated the 'Happy Kids Charter' (the Charter).[3] Referenced from international guidelines, the Charter sets out the broad directions for parents to adopt for nurturing children to develop happily and healthily.[4-6] It contains ten articles covering the following topics: parents' relationship, rest and relaxation, adjustment of expectations, stress-free life, leisure activities, love of nature, communication and interaction, daily exercise, adequate sleep and parent-child reading (Fig. 1). It is generally advised that children of primary school age should have at least 1 hour of exercise and 10-12 hours of sleep every day.[7,8]

In 2018-19 and 2019-20 school years, funded by the District Collaboration Scheme of the Central Western Southern, Island District Social Welfare Office, the Social Welfare Department and the Child and Adolescent Psychiatry team of Queen Mary Hospital promoted the Happy Kids Charter to 9 primary schools in the area. In each of the participating school, a series of activities to promote the happy kids charter, such as gardening, dancing, parents' talk were delivered to the parents and children. Up to a thousand of parents and children joined these activities with positive feedback.

快樂孩子約章 Happy Kids Charter	
1. 父母關係	6. 擁抱自然
2. 休息放鬆	7. 傾計互動
3. 調教期望	8. 每日運動
4. 無拘無束	9. 充足睡眠
5. 閒暇活動	10. 親子伴讀

Fig. 1. Happy Kids Charter (Adapted from https://mentalhealth.edb.gov.hk/uploads/mh/content/resource/Happychildbooklet.pdf)

SURVEY ON PARENTS' ATTITUDE AND PERCEIVED CHILDREN'S STRESS LEVEL

In these participating schools, a survey was conducted in participating schools on parents' attitudes towards the Happy Kids Charter and their perceived children's stress level. Results of 2,306 parents showed that, out of 0-10 with 10 being the maximum stress level, the mean stress levels were 5.56 (SD 1.97) in 2018-19 and 5.13 (SD 2.10) in 2019-20 respectively. No statistically significant difference between children in the upper or lower primary forms was observed (p=0.12). As shown in Table 1, a significant proportion of children had inadequate time for leisure, family time and sleep, all of which were significantly associated with higher perceived stress level. Perceived stress level also significantly correlated with parents' report of children's academic results (p<0.001) with higher perceived stress level among those with worse academic results.

An exceptionally high proportion of parents reported agreement to the Happy Kids Charter (93%) and believed the Charted can improve the overall (86%) and academic performance (70%) of their children as well as improving their mental condition (81%). Most reckoned parent-child relationship being the most important element among the Charter, and yet the most difficult task to carry out. (Fig. 3) The top reasons for difficulties in executing the charter were homework (58%) and revision demand (53%) and unavailability of parents (55%).

[a]*Happy Kids Charter was jointly developed by Child and Adolescent Psychiatry of Queen Mary Hospital under the Hospital Authority, Student Health Service of the Department of Health, Jockey Club South Horizons Children and Youth Integrated Services Centre of the Boys' & Girls' Clubs Association of Hong Kong, Caritas Community Centre - Caine Road, Hong Kong Sheng Kung Hui Welfare Council Limited, Hapi Reading Club, and Queen Mary Hospital's Medical Social Services Unit under the Social Welfare Department*

Table 1. Children's activities and perceived stress level
(Reproduced with permission from the Department of Psychiatry, Queen Mary Hospital)

	2019-20			2018-19		
	**Count (%)**	**Mean stress level (S.D.)**	***p*-value (t-test)**	**Count (%)**	**Mean stress level (S.D.)**	***p*-value (t-test)**
**Average duration of home work**						
< 2 hours per day	249 (69%)	4.8 (2.0)	<0.01	941	5.2 (1.9)	<0.01
>= 2 hours per day	112 (31%)	5.8 (2.1)		1,034	5.9 (1.9)	
**Average duration of exercise**						
No exercise	66 (18%)	5.4 (2.2)	0.22*	269	6.1 (2.1)	<0.05^
< 1 hour per day	273 (76%)	5.1 (2.1)		762	5.6 (2.0)	
>= 1 hour per day	22 (6%)	5.0 (2.0)		898	5.3 (1.9)	
**Average duration of leisure**						
< 1 hour per day	251 (70%)	5.5 (2.1)	<0.01	1,347	5.8 (1.9)	<0.01
>= 1 hour per day	110 (30%)	4.2 (1.8)		598	4.9 (2.0)	
**Average duration of family time**						
< 4 hours per week	198 (55%)	5.4 (2.2)	<0.01	1,184	5.8 (1.9)	<0.01
>=4 hours per week	163 (45%)	4.8 (1.9)		757	5.1 (2.0)	
**Average duration of sleep**						
< 8 hours per day	171 (47%)	5.6 (2.2)	<0.01	242	6.1 (2.0)	<0.01
>=8 hours per day	190 (53%)	4.7 (1.9)		1,717	5.5 (2.0)	

*comparison between those with any and nil exercise
^comparison by ANOVA

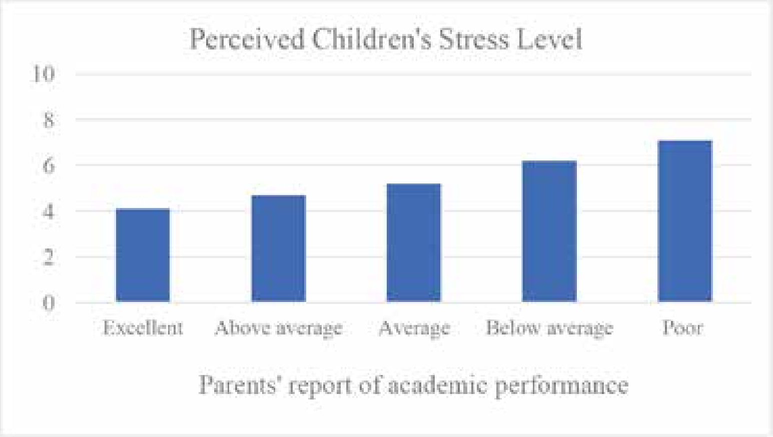

Fig. 2. Perceived children's stress level and children's academic performance reported by parents (2019-20) (Reproduced with permission from the Department of Psychiatry, Queen Mary Hospital)

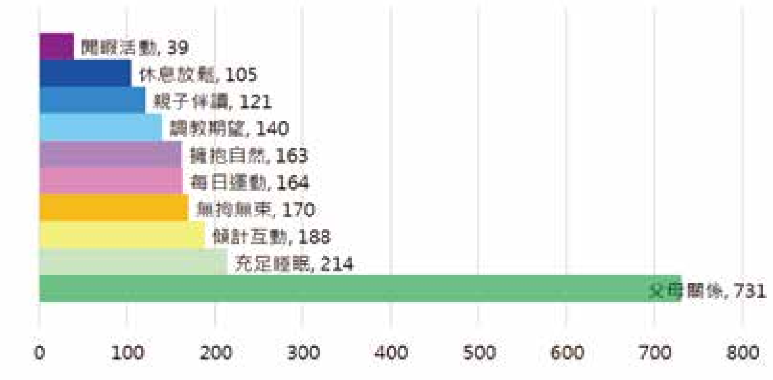

Fig. 3. Parent's report of the most difficult item in the Happy Kids Charter to execute (Reproduced with permission from the Department of Psychiatry, Queen Mary Hospital)

CONCLUSION

Although the findings above did not demonstrate a direct causal relationship, attention should be paid to children who spent less time on exercise orleisure, and those children who spent more time on homework with worse academic performance. Efforts to look into and possibly manage parents' expectation and children's stress level may be beneficial to the children's development and parent-child relationship.

Maintaining a good parent-child relationship is perceived to be the most difficult and the most important task by many primary school parents. At the time of writing, Hong Kong is experiencing an unprecedented pandemic fueled by COVID-19. Schools are suspended and some parents have changes in their work patterns and daily routines. To keep up with a harmonious parent-child relationship may be even more difficult given these alterations. The Happy Kids Charter serves as a timely reminder for readers on how to promote to parents on nurturing a generation of healthy kids for Hong Kong's future. With adjustments in parental expectation and being bestowed adequate time for play, leisure reading and chatting, children shall be able to develop happily despite uncertainties abound.

References

1. Family Council Secretariat. Final Report of the Committee on Prevention of Student Suicides. 2015. (https://www.familycouncil.gov.hk/en/home/files/6_Paper_FC_26_Final%20report%20on%20prevention%20of%20studen.pdf)
2. Nelson CA, Scott RD, Bhutta ZA, et al. Adversity in childhood is linked to mental and physical health throughout life. BMJ. 2020;371:m3048.
3. 「家校齊減壓- 提昇家長及學童的精神健康計劃」籌委. 2015. 快樂孩子約章 (https://mentalhealth.edb.gov.hk/uploads/mh/content/resource/Happychildbooklet.pdf)
4. National Center on Birth Defects and Developmental Disabilities, Division of Human Development and Disability. Positive Parenting Tips for Healthy Child Development. Centre for Diseases Prevention. (https://www.cdc.gov/ncbddd/childdevelopment/positiveparenting/pdfs/middle-childhood-6-8-w-npa.pdf)
5. American Psychological Association. 2019, October 24. How to help children and teens manage their stress. (http://www.apa.org/topics/child-development/stress)
6. Tremblay MS, Gray C, Babcock S, et al. Position Statement on Active Outdoor Play. Int J Environ Res Public Health. 2015 Jun 8;12(6):6475-505.
7. Hirshkowitz M, Whiton K, Albert SM, et al. National Sleep Foundation's updated sleep duration recommendations: final report. Sleep Health. 2015 Dec;1(4):233-243.
8. U.S. Department of Health and Human Services. Physical Activity Guidelines for Americans, 2nd edition. Washington, DC: U.S. Department of Health and Human Services; 2018.

Dermatology Quiz

Dr Lai-yin CHONG

MBBS(HK), FRCP(Lond, Edin, Glasg), FHKCP, FHKAM(Med)

Specialist in Dermatology & Venereology

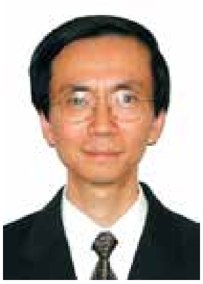

Dr Lai-yin CHONG

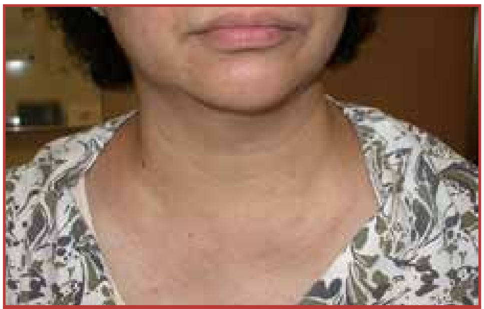

Fig. 1: Erythematous macular lesions at anterior neck

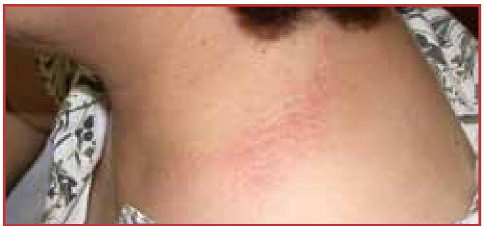

Fig. 2: Erythematous popular lesions at lateral and posterior neck

This 40-year-old lady developed this itchy rash over her neck in past one month (Fig. 1 & 2). There were no skin lesions elsewhere. She attributed these lesions to frequent sun exposure. However, there was no improvement even after strict sun protection.

Questions

1. What important questions should you ask in the history?
2. What is your diagnosis?
3. What test will you order to find out the cause?

(See P.33 for answers)

Take Charge of ADHD

Ritalin LA®

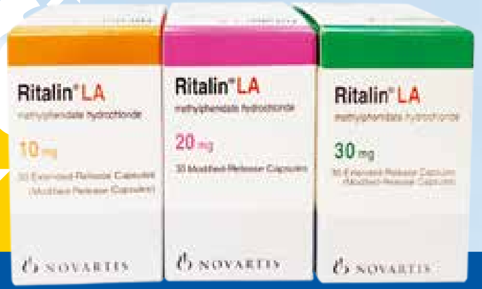

Onset of efficacy at 1 to 1.5 hours[2]

Higher plasma MPH concentration over the first 8 hours than the OROS MPH product[3]

Bimodal release for ONCE-DAILY dosing[1]

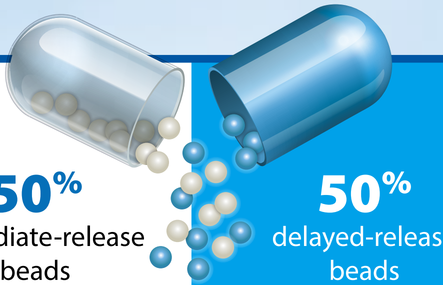

50% immediate-release beads

50% delayed-release beads

Can be taken WITH OR WITHOUT FOOD[1]

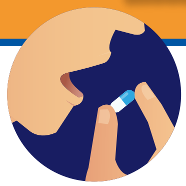

Swallowed whole

OR

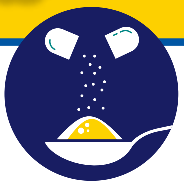

Sprinkled on soft food

Recommended as a FIRST-LINE ADHD treatment[4-7]

American Academy of Pediatrics

NICE National Institute for Health and Care Excellence

CADDRA Canadian ADHD Resource Alliance

Adult ADHD

Children and adolescents

Adults

ADHD=attention deficit hyperactivity disorder. MPH=DL-*threo*-methylphenidate. OROS=osmotic, controlled-release oral delivery system.

RITALIN® / RITALIN® LA

Important note: Before prescribing, consult full prescribing information. **Presentation:** ♦Immediate-release (Ritalin®) tablets containing 10 mg methylphenidate hydrochloride. ♦ Modified-release capsules (Ritalin® LA) containing 10 mg, 20 mg, 30mg methylphenidate hydrochloride. **Indications:** Attention-deficit/hyperactivity disorder (ADHD) in children aged 6 years and older; ADHD in adults (Ritalin LA only); narcolepsy (Ritalin tablets only) **Dosage and administration: ADHD:** Children (6 years and over) Ritalin Tablets: ♦ For children, start with 5 mg once or twice daily and increase in increments of 5 to 10 mg weekly. Ritalin LA Capsules: ♦ for patients who are currently not taking methylphenidate: starting dose is 20 mg. Patients may begin treatment with Ritalin LA 10 mg if necessary. ♦ for patients who are currently taking methylphenidate: the recommended dose of Ritalin LA should be equal to the total daily dose of the immediate-release formulation ♦ Daily dosage should not exceed 60 mg. ♦ Ritalin LA dosage may be adjusted at weekly intervals in 10 mg increments for children ♦ Ritalin LA is for once daily administration. Adult ♦ Only the Ritalin LA formulation should be used for the treatment of ADHD in adults. ♦ for patients who are currently not taking methylphenidate: starting dose is 20 mg. ♦ for patients who are currently taking methylphenidate: the recommended dose of Ritalin LA should be equal to the total daily dose of the immediate-release formulation ♦ Daily dosage should not exceed 80 mg. ♦ Use of Ritalin LA in patients over 60 years of age has not been studies in controlled trials. ♦ Ritalin LA dosage may be adjusted at weekly intervals in 20 mg increments for adults. ♦ Ritalin LA is for once daily administration. Narcolepsy ♦ Daily dosage should not exceed 60 mg. ♦ For adults treated for narcolepsy, the usual daily dose is 20 to 30 mg. **Contraindications:** ♦ anxiety and tension states, agitation, a family history or diagnosis of Tourette's syndrome, glaucoma, hyperthyroidism, pre-existing cardiovascular disorders including uncontrolled hypertension, angina pectoris, arterial occlusive disease especially coronary arteries; heart failure, haemodynamically significant congenital heart disease, cardiomyopathies, myocardial infarction, cardiac arrhythmia and channelopathies (disorders caused by the dysfunction of ion channels), treatment with monoamine oxidase inhibitors, and also within a minimum of 14 days following discontinuation of a monoamine oxidase inhibitor (hypertensive crises may result), phaeochromocytoma, known drug dependence or alcohol abuse; severe depression, anorexia nervosa, psychotic symptoms or suicidal tendency, since Ritalin might worsen these conditions; known hypersensitivity to methylphenidate or to any component of the formulation. **Warnings and precautions:** ♦ Treatment with methylphenidate is not indicated in all cases of ADHD and should be considered only after detailed history taking and evaluation of the patient. The decision to prescribe methylphenidate should depend on the physician's assessment of the chronicity and severity of the symptoms and in paediatric patients, the appropriateness to the child's age. Prescription should not depend solely on the presence of isolated behavioural characteristics. When the symptoms are associated with acute stress reactions, treatment with methylphenidate is usually not indicated. ♦ Generally should not be used in patients with structural cardiac abnormalities or other serious cardiac disorders that may increase the risk of sudden death. ♦ Pre-existing cardiovascular disorders, a family history of sudden death and ventricular arrhythmia should be assessed before initiating treatment. ♦ Caution in patients with pre-existing hypertension. Blood pressure should be monitored during treatment. ♦ Patients who develop symptoms suggestive of cardiac disease should undergo prompt cardiac evaluation. Misuse of stimulants of the central nervous system may be associated with sudden death and other serious cardiovascular adverse events. ♦ Patients with pre-existing cerebrovascular abnormalities should not be treated with Ritalin. ♦ Patients with additional risk factors (history of cardiovascular disease, concomitant medications that elevate blood pressure) should be assessed regularly for neurological/psychiatric signs and symptoms. ♦ Pre-existing psychiatric disorders and a family history of psychiatric disorders should be assessed before initiating treatment. ♦ Should not be initiated in patients with acute psychosis, acute mania or acute suicidality. ♦ In case of emergent psychiatric symptoms (e.g. hallucinations or mania, aggressive behaviour and suicidal tendency) or exacerbation of pre-existing psychiatric symptoms, Ritalin should not be given to patients unless the benefit outweighs the potential risk. ♦ Family history should be assessed and clinical evaluation for tics or Tourette's syndrome in children should precede ADHD treatment. ♦ Patients should be regularly monitored for the emergence or worsening of tics during initiating treatment. ♦ Growth should be monitored during treatment as clinically necessary, treatment interruption may be considered. ♦ Caution in patients with epilepsy. ♦ Patients who develop priapism should seek immediate medical attention. ♦ Not recommended together with serotonergic drugs due to risk of serotonin syndrome. ♦ Chronic abuse can lead to marked tolerance and psychological dependence. ♦ Caution in emotionally unstable patients. ♦ Careful supervision during withdrawal. ♦ Patients requiring long-term therapy should be carefully monitored. Consider appropriate medical intervention in the event of hematological disorders. ♦ should not be used for children under 6 years of age. ♦ Patient should refrain from driving and using machinery if dizziness, drowsiness, blurred vision, hallucination or other CNS side effects occur. ♦ Prolonged and painful erections, sometimes requiring surgical intervention, have been reported with methylphenidate products in both pediatric and adult patients. Priapism was not reported with drug initiation but developed after some time on the drug, often subsequent to an increase in dose. Priapism has also appeared during a period of drug withdrawal (drug holidays or discontinuation). Patients who develop abnormally sustained or frequent and painful erections should seek immediate medical attention. **Pregnancy, lactation, females and males of reproductive potential Pregnancy:** ♦ Ritalin should not be given to pregnant women unless the potential benefit outweighs the risk to the fetus. **Lactation:** ♦ Case reports showed that methylphenidate was distributed into breast milk. A decision should be made whether to abstain from breast-feeding or to abstain from Ritalin therapy, taking into account the benefit of breast-feeding to the child and the benefit of therapy to the woman. **Adverse drug reactions:** ***Very common:*** nasopharyngitis, decreased appetite, nervousness, insomnia, nausea, dry mouth, irritability. ***Common:*** anxiety, restlessness, sleep disorder, agitation, tremor, dyskinesia, headache, drowsiness, dizziness, tachycardia, palpitation, arrhythmias, changes in blood pressure and heart rate (usually an increase), cough, abdominal pain, vomiting, dyspepsia, toothache, rash, pruritus, urticaria, fever, scalp hair loss, hyperhidrosis, arthralgia, feeling jittery, weight decreased. **Rare:** difficulties in visual accommodation, blurred vision, angina pectoris, moderately reduced weight gain and slight growth retardation during prolonged use in children. **Very rare:** leucopenia, thrombocytopenia, anemia, hypersensitivity reactions, including angioedema and anaphylaxis, hyperactivity, psychosis (sometimes with visual and tactile hallucinations), transient depressed mood, convulsions, choreoathetoid movements, tics or exacerbation of existing tics and Tourette's syndrome, cerebrovascular disorders including vasculitis, cerebral hemorrhages and cerebrovascular accidents, abnormal liver function, thrombocytopenic purpura, exfoliative dermatitis, erythema multiforme, muscle cramps. Frequency **not known:** priapism. Very rare reports of poorly documented neuroleptic malignant syndrome (NMS) have been received. In most of these reports, patients were also receiving other medications. It is uncertain what role Ritalin played in these cases. ***Reported with other methylphenidate-containing products***: pancytopenia, auricular swelling, irritability, aggression, affect lability, abnormal behavior or thinking, anger, suicidal ideation or attempt (including completed suicide), mood altered, mood swings, hypervigilance, mania, disorientation, libido disorder, apathy, repetitive behaviors, over-focusing, confusional state, dependence, cases of abuse and dependence have been described, more often with immediate release formulations, reversible ischaemic neurological deficit, migraine, diplopia, mydriasis, visual disturbance, cardiac arrest, myocardial infarction, peripheral coldness, Raynaud's phenomenon, pharyngolaryngeal pain, dyspnea, diarrhea, constipation, angioneurotic edema, erythema, fixed drug eruption, myalgia, muscle twitching, hematuria, gynaecomastia, chest pain, fatigue, sudden cardiac death, cardiac murmur. **Interactions:** ♦ **Concomitant use contraindicated:** MAO inhibitors (currently or within the preceding 2 weeks). ♦ Caution when used concomitantly with drugs that elevate blood pressure, coumarin anticoagulants, anticonvulsants, centrally acting alpha-2 agonists (e.g. clonidine), direct and indirect dopaminergic drugs (e.g. tricyclic antidepressants, DOPA, antipsychotics), phenylbutazone. ♦ **Alcohol:** Patients should abstain from alcohol during treatment. ♦ Ritalin should not be taken on the day of a planned surgery due to risk of sudden blood pressure increase during surgery. ♦ May induce false positive laboratory tests for amphetamines. ♦ Risk of serotonin syndrome with concomitant use of methylphenidate and serotonergic drugs. **Packs and prices:** ♦ Ritalin Tablets: 10mg (200's) ♦ Ritalin LA Capsules: 10mg (30's), 20mg (30's and 100's) and 30mg (30's) **Legal classification:** P1S1S3 Ref: Australia (Oct 2014) (2016-PSB/GLC-0847s + 2017-PSB/GLC-0880-s)

References:

1. Ritalin® 10/ Ritalin® LA Hong Kong prescribing information. 2. Brams M, et al. Postgrad Med 2008;120:69-88. 3. Markowitz JS, et al. Clin Pharmacokinet 2003;42:393-401. 4. Wolraich ML, et al. Pediatrics 2019;144:e20191682. 5. Canadian ADHD Resource Alliance (CADDRA). Canadian ADHD Practice Guidelines, Fourth Edition. CADDRA 2018. 6. National Institute for Health and Care Excellence. Attention deficit hyperactivity disorder: diagnosis and management 2018. Available at: www.nice.org.uk/guidance/ng87 (Accessed on: 18 Nov 2019). 7. Kooij JJS, et al. Eur Psychiatry 2019;56:14-34.

HK2107065075

NOVARTIS

Novartis Pharmaceuticals (HK) Ltd
7/F, Citi Tower, One Bay East, 83 Hoi Bun Road,
Kwun Tong, Kowloon, Hong Kong
Tel: (852) 2882 5222 Fax: (852) 2577 0274

Frontline Social Workers' Experience in the Implementation of the Parent Education in A Mental Health Project at Primary Schools

Ms Yick-man LAM

RSW, Senior Manager, Youth Services, St. James' Settlement

Ms Bubble SW LEE

MSSc, PDG(Psy), BSW, RSW

Supervisor, Ho Yuk Ching Educational Psychology Service Centre (Sha Tin), Tung Wah Group of Hospitals

Ms Mei-ling WONG

Supervisor, Pre-school education & special projects
The Boys' & Girls' Clubs Association of Hong Kong
MBA, University of South Australia
MSW, The Hong Kong Polytechnic University

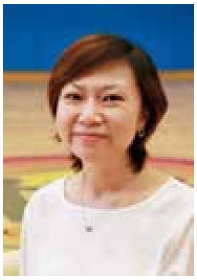
Ms Yick-man LAM

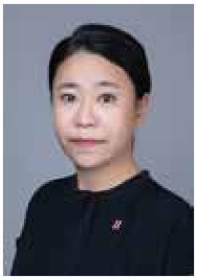
Ms Bubble SW LEE

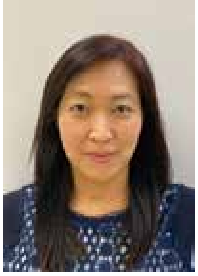
Ms Mei-ling WONG

INTRODUCTION

The "Strategic Work in Parent Education in Mental Health – Primary School" project, led by the Hong Kong College of Psychiatrists, aimed to apply an age-specific parenting framework that was previously validated empirically by the Child and Adolescent Psychiatry Team at Queen Mary Hospital. Based on the framework, a six-year parenting training curriculum was developed and customised for school settings. Frontline social workers from the Boys' & Girls' Clubs Association of Hong Kong, St James' Settlement, Tung Wah Group of Hospitals were trained to implement the programme for six primary schools during the 2020-2022 academic years.

SCHOOL COOPERATION AND LIAISON IN PARENT EDUCATION

The best educational outcomes can be achieved when teachers, students and parents collaborate in a healthy partnership.

To lay the groundwork that fosters this partnership, the priority is to gain the support of the school principals, who can mobilise the guidance and counselling team as well as every form's headteachers to encourage parents to attend parenting education courses. Some principals further formulate policies to provide incentives to mobilise parents' active participation in parenting courses and promote the culture of continuous learning among parents regarding their children's growth in various stages. In brief, it is important for parents to believe that the schools are willing to involve parents in children's education, and that the school educators will provide guidance and support for the parents.

The most accurate predictor of students' academic achievement is neither socioeconomic status nor how prestigious the school is. It is the extent to which families encourage learning at home and involve themselves in their children's education.[1]

Teachers who focus on parent engagement could often see a profound change in their classrooms when parents are engaged. The greater the extent parents get involved in their children's education, the better their entire class's motivation, behaviour, and grades become.[2] Besides, it is also important to enhance teachers' perspectives in educating students not only as individuals but also as the whole family behind the students. As teachers are often busy with teaching and related administrative work and school social workers are occupied with handling students in crisis, they can hardly spare time for parent engagement even though they know that involving parents is essential. If a third party can line up with school social workers and teachers through delivering parenting education and conducting formal or informal meetings at schools from time to time with parents, schools can formulate better need assessments of parents and provide more support for students. From the perspective of parents, when they gradually understand that schools put emphasis both on children and parenting education, they are encouraged and motivated to participate actively in school.

PARENT ENGAGEMENT SKILLS

In this project, social workers work closely with parents via talks, workshops and groups. Parents need to feel that they are active participants in partnership with social workers. Recognising parents' expertise on their own lives and their children's lives, doing things with families rather than to the families is crucial. (Moran and others 2004)

Social workers will listen and respond to what service the families prefer to have and how and when the service could be provided. From frontline experience, the following elements are very important in engaging parents:

1. Respect: appreciating parents as individuals, trusting their fundamental ability to manage and make a difference in their family lives, and inviting parents to share their parenting experience without being judgmental.

2. Genuineness: being sensitive, honest and trustworthy.

3. Empathy: showing an empathetic response to the difficulties and challenges parents are facing in their lives, and being able to perceive the circumstances from parents' point of view.

4. Friendliness: bringing in an approachable, encouraging atmosphere and a consistently calm, steady and warm approach

5. Expertise: the knowledge and experience brought in by the social workers to complement the parent's existing knowledge and skills, both in building the relationship and in providing information and support.

The key to successful parent engagement is working in partnership. Effective communication with active listening is key to building rapport with parents, be it at one-to-one occasions such as at pre-training group interview, caring call to parents in between workshop sessions, post-group interview and at-group training sessions. We can give individual support to parents if needed either in the programme or at other appropriate and convenient times for parents.

CASEWORK INTERVENTION FOR HARD-TO-REACH PARENTS (NON-ENGAGED PARENTS)

The phrase 'hard to reach' or "non-engaged parents" is often used to describe parents who do not access services nor join any school activities and programmes. However, these parents often have parenting difficulties and need support. This non-engagement could be accounted for by the inaccessibility of some parents as well as that the services are hard to access for some parents. There are agency barriers to overcome, such as the timing of the programmes and travel distance, and barriers connected to parents, such as their attitude and feelings.

To engage parents who traditionally do not access services, social workers need to consider providing the following:

- Time - Enough time should be given to develop relationships with families in a way that is acceptable to the families. Social workers can show concerns about the parent's needs and parenting difficulties through WhatsApp messages and regular phone calls.

- Support - One-to-one support would be provided to parents who have difficult to practise their parenting skills. In addition, social workers have to be alert of crisis, such as suspected child abuse, lack of financial and materials support during epidemic and to provide timely intensive support and crisis intervention.

- Providing community resources - Social workers could make flexible and responsive decisions about how best to support families by involving other parties where necessary, such as family services and school social work services.

- Fine-tuning to Covid time - Social workers should fine tune their support on specific needs and circumstances, such as providing online parent workshops during the epidemic on the topics related to parents' mental health and wellbeing during this period.

- Peer support - Parents from Parent-Teacher Association could act as peer supporters and help to share parenting experience and deliver parenting skills. They can actively approach some non-engaging parents through different networks and encourage parents to seek help or join the parenting activities.

It is important, in some cases, social workers have to 'take the service to the parent' rather than wait for the parent to come. By providing flexible non-stigmatising universal parenting programme and services, social workers can establish relationships with families in a natural setting. Families can then access support from social workers when needed. In case some families may need intensive or crisis services, it would be easier for the families if support is offered by familiar and trusted social workers who have developed a relationship with parents.

For example, a mother suspected her primary 2 daughter had special needs in studying. But she was hesitant to disclose to the school and thus could not receive any service. After joining the training group and having attended one-to-one case consultation, the mother was willing to seek service for her daughter and to be more empathetic and genuine in the parent-child relationship. Building such relationships takes time and this continuity of care is important in maintaining a trusting relationship with families.

CHANGE OF LEARNING MODE

The impact of COVID-19 on students and schools over the past two years was tremendous and ever-changing, with online learning replacing in-person environments during more critical periods. The project team adapted in similar ways, by planning and incorporating online programmes to supplement in-person teaching. This online format offered an accessible learning environment for parents, catering to the needs of the community during the pandemic, with repeated school suspensions, online lessons, and working from home arrangements becoming the new norm. Along with these changes, parents were able to participate in this project online, gaining knowledge and skills in handling children's mental health as well as Covid-related issues.

FEEDBACK FROM PARENTS

Feedback from parents and caregivers was collected through focus groups and individual sharing after training sessions. Participants who had joined at least one of the project activities were invited to give feedback.

1. The structured age-specific parenting curriculum was highly appreciated. The majority of parents found the age-specific parenting knowledge and skills taught was useful. They also appreciated the teaching of structured mental health knowledge, as it helped them to strengthen the emotional bonding with their

children, while at the same time improving their own stress management skills. All parents agreed that an age-specific parenting curriculum should be systematically promoted and delivered during the primary school years.

2. Parent workshops and coaching sessions were helpful in relieving parental stress. Parents found that they gained a better understanding of their children's needs by learning about the emotional and social developmental stages during the primary school years. Parents reported that coaching sessions were particularly useful as facilitators used real-life examples to illustrate specific skills. The parents appreciated being offered the chance to share their parenting difficulties during the workshops. They also derived insights into new ways of dealing with specific parenting challenges during the coaching session.

3. Parents appreciated the schools' efforts in promoting parent education. The project team and the school implemented an award scheme to rally parents' participation. Parents who attended more than half of all the sessions reported that their self-efficacy in parenting skills increased. They became more confident in applying the knowledge and skills they had learned into daily life. In addition, parents appreciated the respective school's effort to promote parent education and this project in general.

4. The project encouraged help-seeking from professionals in times of need. Low-income parents reported that the project provided an effective channel for them to seek help on parenting skills. Parents were more willing to reach out to school teachers and social workers, as the latter had a better understanding of the former's difficulties and limitations. Parents also commented that the project had encouraged them to make use of the school support services available to them.

5. A comprehensive parent-child programme was recommended. Looking forward, parents suggested having a more comprehensive programme curriculum for them to learn with their spouse and children together as a group (e.g., parent-child workshops). They showed interest in topics such as helping their children manage stress, relaxation and mindfulness techniques, and conflict resolution in marital relationships.

FEEDBACK FROM SCHOOL

Feedback was also collected from school principals, teachers, school social workers, and Parent-Teacher Association members.

1. School involvement would enhance parents' participation. In addition to the aforementioned participation award scheme, project activities were integrated and promoted into the content of various school events, including "Primary One Adaptation Programme" and "Parents' Day". School principals and teachers felt that this was helpful in increasing parent interest and participation in the project.

2. Structured programme enriched parents' understanding of child development. The school guidance teachers and social workers appreciated the structure of the project curriculum, as it helped parents learn the skills and knowledge in a progressive manner. They also found that the workshops helped the parents enhance their understanding of their children's social-emotional needs and developmental issues, which in turn helped them address difficulties more effectively.

3. Early identification of family needs and crisis. Parent-Teacher Association members also responded positively to the project, as they expressed that the project encouraged parents to reflect on their different family roles. Parents became more willing to seek consultation from project workers and school personnel to address their familial issues, leading to earlier identification of possible family crises and more timely referrals of suitable community resources to help address parenting and emotional challenges.

CONCLUSION

Given that the primary school years are a prime time for a child's mental development, a systematic and age-specific parenting programme is highly recommended as a preventive and supportive tool to equip parents with sufficient knowledge and skills for nurturing their children. The "Strategic Work in Parent Education in Mental Health – Primary School" project aims to do just that, by putting forth a parenting curriculum based on a mental health support framework that can help parents with primary school-aged children. Furthermore, the project encourages even closer home school cooperation with the wider goal of providing a better environment for growth and learning for students.

The authors would like to thank Ms Carmen Yuet-ying CHEONG, Ms Sau-mei LAW, Ms Sybil Wing-sik WONG for their contribution in this article by sharing their frontline experiences.

References

1. PTA, N. (2000). Building Successful Partnerships: A Guide for Developing Parent and Family Involvement Programs. (pp. 11-12). Bloomington, Indiana: National PTA, National Education Service.
2. Ferlazzo, J. (2011, May). Involvement or Engagement? ASCD, pp. 10-14.

唯穩思™
Vyvanse®
(lisdexamfetamine dimesylate)

No.1 ADHD MEDICATION[2] IN THE WORLD

Available now!

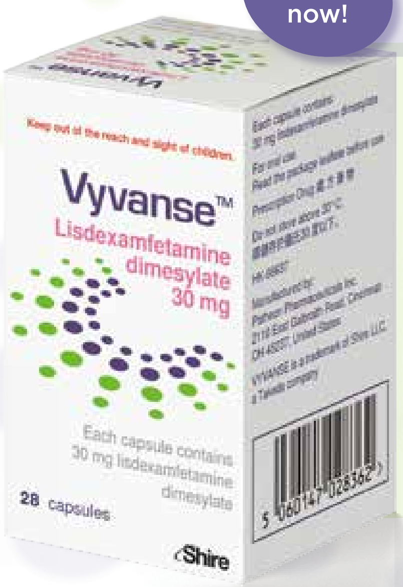

Prodrug formulation for
ADHD TREATMENT
in Children, Adolescents & Adults[1]

Indication: VYVANSE® is indicated for the treatment of Attention Deficit / Hyperactivity Disorder (ADHD)[1]

- **Novel Prodrug formulation** in ADHD treatment[1]
- **Once daily, Capsule or Capsule free administration**[1]
- Long acting efficacy **for up to 13 Hours in children 6-12 year old**[3*] **& up to 14 hours in adults**[4#]

* Study design: Study 311 was a randomised, double-blind, multicentre, placebo-controlled, crossover, analogue-classroom study14 of children (6 - 12 years) with a primary diagnosis of ADHD. Following screening (~3 weeks) and washout (up to 1 week), eligible subjects received open-label VYVANSE, titrated from 30 mg QD, to reach an optimal dose in 4 weeks. Afterwards, 117 patients were randomised (1:1) to receive VYVANSE at the optimised QD dose × 1 week followed by placebo QD × 1 week or vice versa, in a double-blind manner. The primary end point was the SKAMP-D subscale score at 1.5 hours post-dose on the last day of each week (dosed at 7 a.m.)

Study design: Study 316 was a randomised, double-blind, placebo-controlled, 2-way crossover study21 of adults (18 - 55 years) with a primary diagnosis of ADHD, conducted in a simulated adult workplace environment (AWE). Following a 4-week, open-label dose-optimisation phase, 127 patients were then randomized to receive their optimised dose of VYVANSE × 7 days followed by placebo × 7 days or vice versa. The primary efficacy end point was the total PERMP scale scores averaged over all post-dose time points during the visits at week 5 and 6

Abbreviated Prescribing Information - VYVANSE® (lisdexamfetamine dimesylate) Capsules 20 mg, 30 mg, 50 mg (Hong Kong PI Nov 2019)
Indication: VYVANSE is indicated for the treatment of Attention Deficit Hyperactivity Disorder (ADHD) **Dosage:** The recommended starting dose is 30 mg once daily in the morning in patients ages 6 and above. Dosage may be adjusted in increments of 10 mg or 20 mg at approximately weekly intervals up to maximum dose of 70 mg/day. **Administration:** Take VYVANSE by mouth in the morning with or without food; avoid afternoon doses because of the potential for insomnia. Swallow VYVANSE capsules whole, or open capsules, empty and mix the entire contents with yogurt, water, or orange juice. Do not take anything less than one capsule per day. A single dose should not be divided. **Contraindications:** Known hypersensitivity to amphetamine products or other ingredients of Vyvanse. Patients taking monoamine oxidase inhibitors (MAOIs), or within 14 days of stopping MAOIs. **Warnings & Precautions:** Potential for abuse and dependence, serious cardiovascular reactions, blood pressure and heart rate increases, psychiatric adverse reactions, suppression of growth, peripheral vasculopathy including Raynaud's phenomenon, serotonin syndrome. **Adverse Reactions (incidence ≥5% and at a rate at least twice placebo):** reported in children, adolescents, and/or adults were anorexia, anxiety, decreased appetite, decreased weight, diarrhea, dizziness, dry mouth, irritability, insomnia, nausea, upper abdominal pain, and vomiting.
Full prescribing information is available upon request.

References: 1. Vyvanse (lisdexamfetamine dimesylate) capsules Hong Kong Prescribing information Nov 2019 version **2.** DOF: IQVIA worldwide sales data MAT 09/2021 **3.** Wigal SB et al. Child Adolesc Psychiatry Ment Health 2009;3:17. **4.** Wigal T et al. 316 study group. Behav Brain Funct 2010;6 :34

To Report Suspected Side Effects for Takeda Products
AE.HongKong@takeda.com

Medical Information and other Inquiries for Takeda Products
MedinfoHK@takeda.com

Takeda

Takeda Pharmaceuticals (Hong Kong) Limited
23/F & 24/F East Exchange Tower,
38 Leighton Road, Causeway Bay, Hong Kong
Tel : 2133 9800 Fax : 2856 2728

C-APROM/HK/Vyv/0043 (03/2022)

The Impact of Covid-19 on the Mental Health of Primary School Parents in Hong Kong and Overseas

Dr Vincent YC TANG
MBChB (CUHK), MRCPsych (UK), FHKCPsych, FHKAM (Psychiatry)
Specialist in Psychiatry
Associate Consultant, Department of Psychiatry, Queen Mary Hospital

Dr Krystal CK LEE
MBBS (HKU), FHKCCM (Public Health Medicine), FHKCPsych, FHKAM (Community Medicine), FHKAM (Psychiatry)
Specialist in Psychiatry
Associate Consultant, Department of Psychiatry, Queen Mary Hospital

Dr Roger NG
President, the Hong Kong College of Psychiatrists

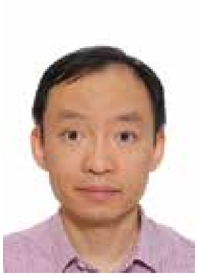
Dr Vincent YC TANG

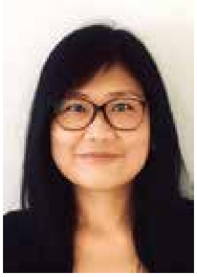
Dr Krystal LEE.

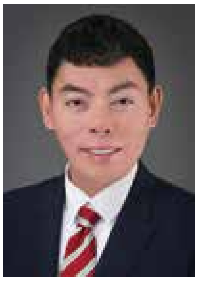
Dr Roger NG

INTRODUCTION

Multiple waves of Covid-19 outbreaks in Hong Kong have resulted in the periodic closure of schools, playgrounds, beaches, museums and amusement parks. To lower the chance of catching Covid-19, many kids have been kept at home much of the time. Parents have had to pick up the slack, becoming full-time teachers and coming up with various activities that can be done at home. Some parents have also had to work from home during Covid-19, increasing the time spent together with their children as well as the chance for conflict. Children and adolescents may have difficulty adjusting to the unpredictable changes in their academic, social and family lives, whereas parents may simultaneously be facing unemployment, reduced income or other uncertainties as Covid-19 disrupts the economy. These stressors could induce emotional and behavioural changes in both children and their caretakers, and could even result in family breakdown or child abuse. For example, the number of child abuse cases listed in the Child Protection Registry in Hong Kong jumped to 1,367 in 2021, a rise of 45% from 940 in 2020. The number of child abuse cases had been stable previously: 1,006 in 2019 and 1,064 in 2018.[1] Thus the pandemic, a temporary phenomenon, could cause a lasting impact if parents are unable to cope. The plight of parents of children with special needs may be even more severe: the closure of special schools and outbreaks at hostels cum day activity centres would mean the loss of training and the temporary return to the homes of these children who are difficult to care for in the home setting.

LITERATURE REVIEW

Recent studies in Hong Kong and overseas have tried to quantify the impact of Covid on parents. Such studies are difficult to compare, however, due to the different methodologies and measures used, as well as the different underlying populations and severity of Covid and Covid-related social distancing measures in the respective jurisdictions. This review will only include published studies on mental health disorders and not other constructs such as parental stress or well-being.

A longitudinal study by Feinberg et al. in the US followed 129 family dyads with an average of 2.3 children before and during the pandemic. Using the Strengths and Difficulties Questionnaire (SDQ, a 25-item measure reported by caregivers), the percentage of children with emotional and peer relationship problems increased from 5.3% to 12.1%, and the percentage of children with conduct and hyperactivity problems increased from 12.1% to 16.5%. Using the Centre for Epidemiological Studies-Depression scale (CES-D, a 20-item measure reported by caregivers), the percentage of parents with depression rose from 13.6% prior to the pandemic to 34% of parents during the pandemic.[2]

A Hong Kong cross-sectional study by Chan et al. compared 51 parents of children with developmental disorders (often denoted as Special Educational Needs, SEN) versus 78 parents of children with typical development from 12 mainstream schools during the first wave of the pandemic in May 2020. Using the Patient Health Questionnaire-9 (PHQ-9, a self-reported 9-question instrument to screen for depression), 25.5% of parents (n = 13) of developmentally-disordered children had depression compared to 10.3% of parents (n = 8) of typically-developed children ($x^2 = 5.25$, $p = .02$). Using the General Anxiety Disorder-7 (GAD-7, a self-reported 7-question instrument to screen for anxiety), no significant difference was found in the prevalence of probable generalised anxiety disorder between parents of children with developmental disorders (13.7%, n = 7) and those of children with typical development (5.1%, n = 4), ($x^2 = 2.92$, $p = .09$).[3] To take this into perspective, Choi et al. in 2020 found 19% had depression and 14% had anxiety in a random population-based sample of Hong Kongers[4].

PRELIMINARY FINDINGS FROM THE HONG KONG COLLEGE OF PSYCHIATRISTS

The Hong Kong College of Psychiatrists also conducted a cross-sectional study on the mental health of various populations in Hong Kong during the Covid-19 pandemic as part of the College's Care4ALL programme. Parents of primary school children were one of the populations studied. 633 parents were recruited from 8 primary schools from 12 June through 4 November 2020 (during the 3rd wave), and filled in an online self-reporting survey. PHQ-9 was used to screen for depression, GAD-7 for anxiety and the Impact of Event Scale-Revised (IES-R, a 22-item self-report measure for DSM-IV that assesses subjective distress caused by traumatic events) for Post-Traumatic Stress Disorder (PTSD).

The full results of the Care4ALL study will be published in due course, but some preliminary findings are shown here to enlighten our readers. A significant number of parents suffered mental illness during the third wave of Covid-19. 13% of parents had moderate to severe depressive symptoms as scored by the PHQ-9. 13% of parents had moderate to severe anxiety symptoms as scored by the GAD-7. 14% of parents had significant PTSD symptoms as scored by the IES-R. 22% of parents scored clinically for at least one of depression, anxiety or PTSD, whereas 6% scored clinically for all three.

Parents of children with special educational needs fared significantly worse. Of those with Attention Deficit Hyperactivity Disorder (ADHD) children, 47% had moderate to severe depressive symptoms, 47% had moderate to severe anxiety symptoms and 49% had significant PTSD symptoms. Of those with Autism Spectrum Disorder (ASD) children, 43% had moderate to severe depressive symptoms, 37% had moderate to severe anxiety symptoms and 37% had significant PTSD symptoms.

SUMMARY

Covid-19 has adversely affected the mental health of parents. Fig.1 shows the point prevalence estimates of mental health disorders derived from the studies reviewed herein. The methodology, the severity of Covid-19 at the time and place and the underlying population differences are just some of the potential factors accounting for the differences. Note that the Hong Kong studies were done before the latest and most severe "Omicron" fifth wave, whereas the Feinberg et al. study took place in the US at a time when the actual pandemic might have been more severe than in Hong Kong.

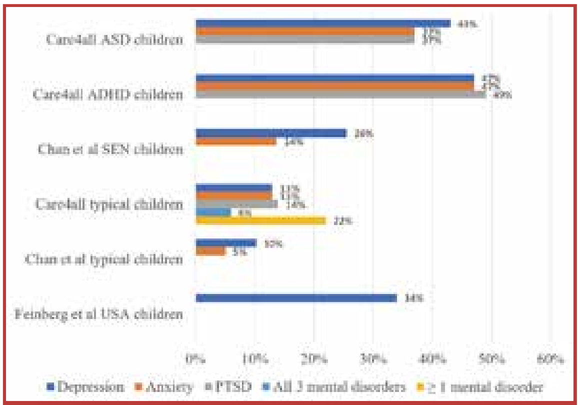

Fig. 1: Point Prevalence of Parents with Mental Disorders with typical and SEN children (Adapted from the studies reviewed in this article, namely Chan et al[3], Feinberg et al[2], HKCPsych Care4all programme[5])

It will be important to conduct longitudinal follow-up studies to observe changes before, during and after the pandemic to gain a fuller understanding of the impact of the pandemic on parents' mental health and of the factors that moderate it. This approach can also quantify the cumulative effects of Covid as we enter the third year of the ever-mutating virus.

Targeted mental health interventions to support parents at this crucial time may be able to lessen the mental health consequences for both parents and children, especially those with SEN. Social distancing measures can also be designed with safe ways to preserve some social and leisure activities to prevent mental health problems. Government public campaigns could help reassure the populace about the perceived threat and subjective vulnerability by stressing the safety and importance of vaccination.

References

1. Wong, Dennis. Child Abuse in Hong Kong. South China Morning Post 30/3/2022. https://multimedia.scmp.com/infographics/news/hong-kong/article/3172267/child-abuse/index.html
2. Feinberg M.E., Mogle J.A., Lee J.K. et al. Impact of the COVID-19 Pandemic on Parent, Child, and Family Functioning. Family Process. 2021; 61(1): 361-374.
3. Chan, R.C.H., Fung, S.C. Elevated Levels of COVID-19-Related Stress and Mental Health Problems Among Parents of Children with Developmental Disorders During the Pandemic. Journal of Autism and Developmental Disorders. 2022; 52: 1314–1325.
4. Choi E.P.H., Hui B.P.H., and Wan E.Y.F. Depression and Anxiety in Hong Kong during COVID-19. International Journal of Environmental Research and Public Health. 2020; 17(10):3740.
5. Hong Kong College of Psychiatrists Care4all Programme, unpublished data.

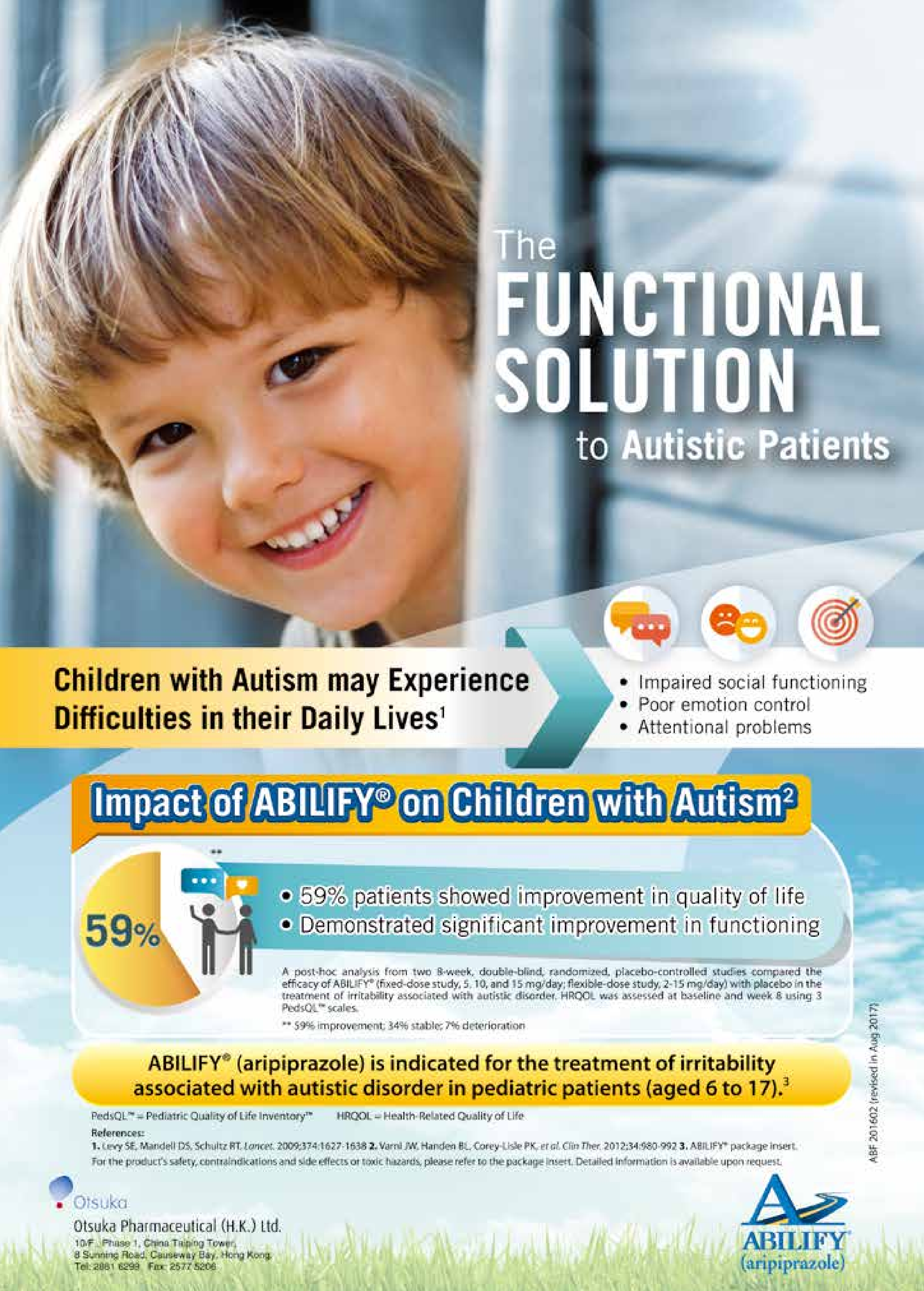
The FUNCTIONAL SOLUTION to Autistic Patients
Children with Autism may Experience Difficulties in their Daily Lives[1]
• Impaired social functioning
• Poor emotion control
• Attentional problems
Impact of ABILIFY® on Children with Autism[2]
59%
**
• 59% patients showed improvement in quality of life
• Demonstrated significant improvement in functioning
A post-hoc analysis from two 8-week, double-blind, randomized, placebo-controlled studies compared the efficacy of ABILIFY® (fixed-dose study, 5, 10, and 15 mg/day; flexible-dose study, 2-15 mg/day) with placebo in the treatment of irritability associated with autistic disorder. HRQOL was assessed at baseline and week 8 using 3 PedsQL™ scales.
** 59% improvement; 34% stable; 7% deterioration
ABILIFY® (aripiprazole) is indicated for the treatment of irritability associated with autistic disorder in pediatric patients (aged 6 to 17).[3]
PedsQL™ = Pediatric Quality of Life Inventory™ HRQOL = Health-Related Quality of Life
References:
1. Levy SE, Mandell DS, Schultz RT. Lancet. 2009;374:1627-1638 2. Varni JW, Handen BL, Corey-Lisle PK, et al. Clin Ther. 2012;34:980-992 3. ABILIFY® package insert.
For the product's safety, contraindications and side effects or toxic hazards, please refer to the package insert. Detailed information is available upon request.
ABF 201602 (revised in Aug 2017)
Otsuka
Otsuka Pharmaceutical (H.K.) Ltd.
10/F., Phase 1, China Taiping Tower,
8 Sunning Road, Causeway Bay, Hong Kong.
Tel: 2881 6299 Fax: 2577 5206
ABILIFY
(aripiprazole)

Inspecting the World in Another Perspective - The World of Macrophotography

Dr Victor NS WONG
FRPS, GPSA, AFIAP

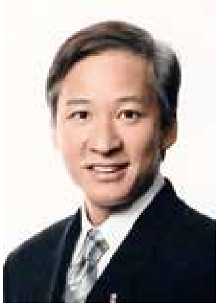

Dr Victor NS WONG

As an outcome of the pandemic, we've had much more time to pursue interests that we usually can't by virtue of our careers. Photography is one of them.

I believe that everyone has artistic potential within themselves. A quality untapped and to be discovered. Perhaps with a bit of inspiration, your artistic side will be catalysed. Of course, this comes with learning and practising, but most importantly, a powerful drive (not the camera!).

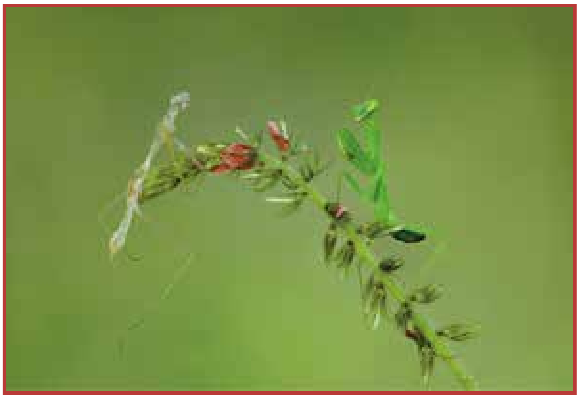

Praying Mantis and its newly shedded skin (Personal collection)

I first began macro-photography five years ago and became more intrigued by insect photography during the pandemic. I started spending much of my free time looking for interesting insects in the local parks of Hong Kong. I was surprised to find a large variety of these little creatures surrounding us. They hide inconspicuously in their own natural yet glamorous wonderlands. Probably due to the hot, humid climate in this locality, these invertebrates are very active and colourful while also exhibiting a diversity of behaviours. In my opinion, the best time to explore this miniature world should be in the spring and the fall.

Searching for insects/invertebrates is quite a task. I must deliberately avoid areas with insecticides, the use of which is unfortunately prevalent in most country parks. This also means that often I have to work in intense heat and humidity while being surrounded by mosquitoes!

My recent visits to different reserves have demonstrated to me how local wildlife can adapt and thrive in the smallest of habitats. Life spans of insects are incredibly short (usually few weeks, rarely beyond a year). It's quite common to find totally different characters after a few weeks at the same location.

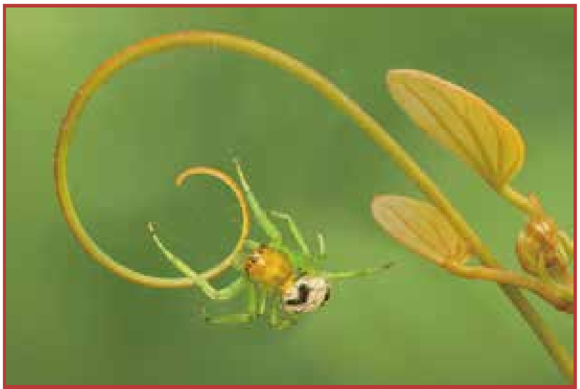

Kidney Garden Spider (Personal collection)

Macro-photography requires a lot of practice. I feel that with time, I have improved gradually since my first day. Now I can locate at least 5-6 different species during each trip, as I gaze carefully underneath leaves or branches, or even on handrails. Paying attention to small inconspicuous spots on the plants can offer big rewards sometimes. A little bit of flashlight helps a lot to locate interesting subjects.

I would start as early as five in the morning when the temperature was a bit cooler and insect movement was minimal. I portrayed insects in their natural environment and close relationship with local flora, featuring seasonal behaviours that included feeding, mating, parental care, and peer grouping. I tried to look for backlit subjects, which created dramatic and vibrant images. There were also curled perches that gave reasonable separation of the subjects against the background, which made the scenes look marvellous. As the sun rose, insect activity would increase. Simple gestures like loud sounds or blowing air sometimes halted the insects' movements for a couple of seconds, just enough for a rapid capture.

With my macro lenses, I used a technique called focus stacking. It means to shoot continuously at a subject from the head to the tail and to composite the images together afterwards to ensure sharpness from head to tail. For this, I mostly used a Nikon D850 mounted on a tripod. For moving subjects, I would use high-speed continuous handheld manual shooting mode to achieve an end-to-end sharpness. A fill flash was

used when needed. I kept my shutter speed constantly at 1/100 to 1/200 sec, aperture f/4 to f/8 on continuous focus stacking while f/8- f/16 on single shots. The ISO ranges from 100 to 1,600. I usually kept shooting very close to my subjects under natural light conditions. Occasionally I would use my Sony A1, 100-400mm lens with 10mm autofocus extension tube for dragonflies and damselflies. This camera was quick enough at high-speed continuous shooting mode to focus stack a slowly moving insect in a second.

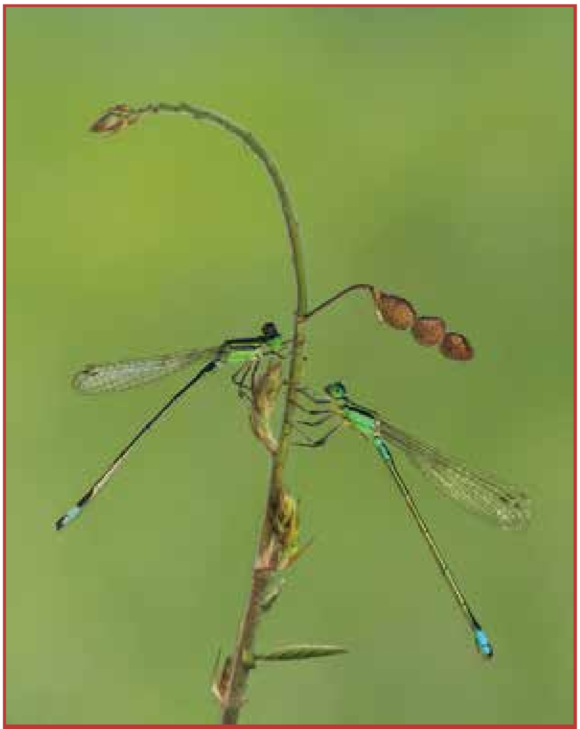
Common Bluetail Damselfies (Personal collection)

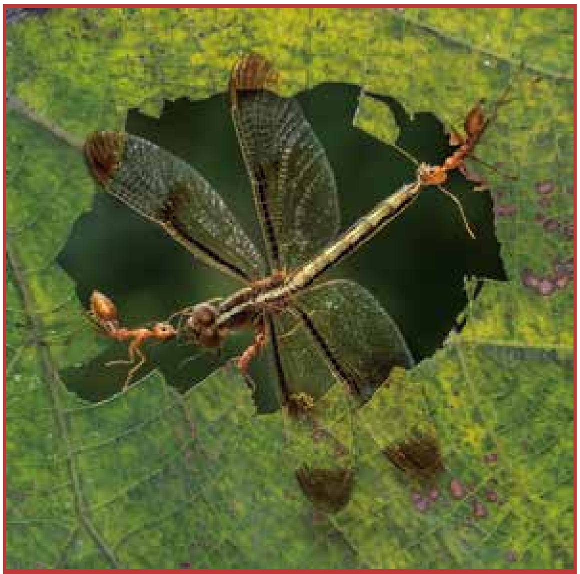
Pied Paddy Skimmer Dragonfly captured by Weaver Ants (Personal collection)

Failure is a frequently occurring thing. There are thousands of pictures you will end up deleting. If you have one good picture, then it's worth it!

This is my homage to a small world that lies hidden away, yet very near and dear to all of us.

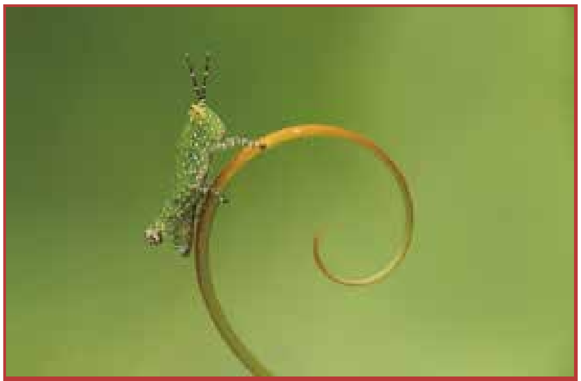
Rice Grasshopper (Personal collection)

Certificate Course for Healthcare Professionals • Course No. C378 • CME/CNE Course

Certificate Course on

Cytogenomics 2022

(Video Lectures)

Jointly organised by

The Federation of Medical Societies of Hong Kong

The Hong Kong Society of Cytogenomics

Objectives:

Introduction of cytogenomics, new approaches in science and technologies for Genetic and Genomic studies in science and medicine

Date	Topics	Speakers
9 June 2022	Understanding Genes, Chromosomes, Inheritance and Diseases	Chev. Chan Wing Kwong<br>President of The Hong Kong Society of Cytogenomics<br>Specialist in Cytogenetics
16 June 2022	The Ethical Issues in Genetic Counselling	Dr. Lam Tak Sum, Stephen<br>Honorary Professor<br>Faculty of Medicine<br>Chinese University of Hong Kong
23 June 2022	The Clinical Applications of Short-and Long-Read NGS Approaches	Dr. Chan Tsun Leung, Chris<br>Honorary Associate Professor<br>Department of Pathology<br>The University of Hong Kong
30 June 2022	Recent Advances in Prenatal Diagnosis	Dr. Kan Sik Yau, Anita<br>Consultant<br>Department of Obstetrics & Gynaecology<br>Queen Mary Hospital
7 July 2022	Cytogenetics in Haematological Malignancies	Dr. Wong Wai Shan<br>Consultant, Department of Pathology<br>Queen Elizabeth Hospital
14 July 2022	Cytogenetics of Postnatal and Constitutional Disorders	Dr. Luk Ho Ming<br>Consultant<br>Clinical Genetics Service Unit<br>Hong Kong Children's Hospital

Date : 9, 16, 23, 30 June & 7, 14 July 2022 (Every Thursday)
Duration of Session : 1.5 hours (6 sessions)
Time : 7:00 pm – 8:30 pm
Course Feature: Video lectures (with Q&A platform for participants to post the questions)
Quiz for doctors: DOCTORS are required to complete a quiz after the completion of each lecture
Language Media : Cantonese (Supplemented with English)
Course Fee : HK$1,000
Certificate : Awarded to participants with a minimum attendance of 70% (4 out of 6 sessions)
Deadline : 2 June 2022
Enquiry : The Secretariat of The Federation of Medical Societies of Hong Kong
Tel.: 2527 8898 Fax : 2865 0345 Email : vienna.lam@fmshk.org

CME / CNE / CPD (Medical Laboratory Technologists) Accreditation in application
Online Application from website: http://www.fmshk.org

THE FEDERATION OF MEDICAL SOCIETIES OF HONG KONG
香 港 醫 學 組 織 聯 會

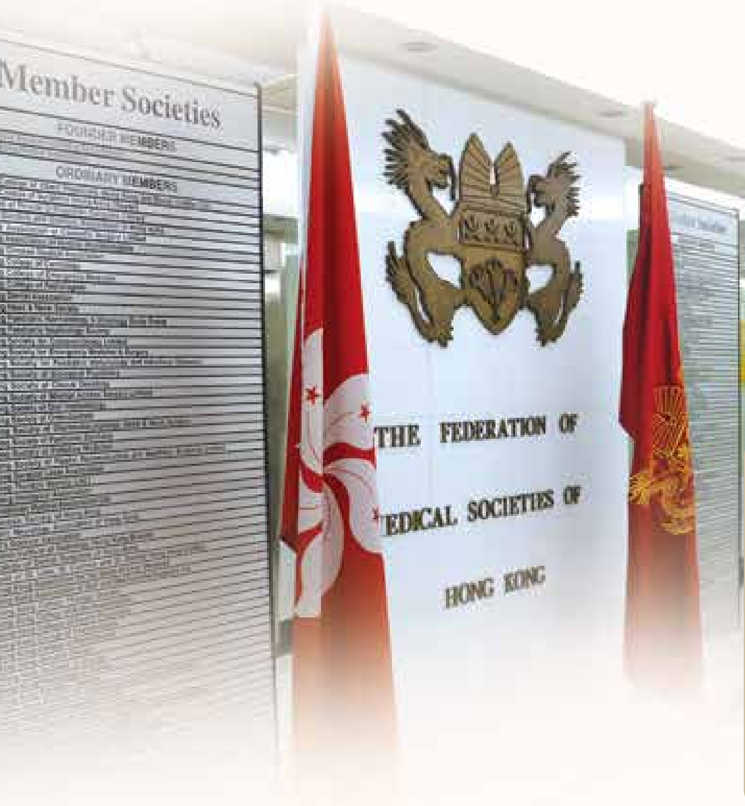

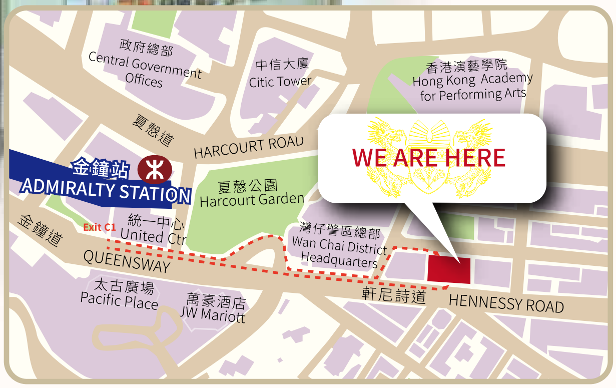

Location: **4/F., Duke of Windsor Social Service Building, 15 Hennessy Road, Wan Chai, Hong Kong**

ROOM RENTAL PROMOTION
Book now & get FREE 2 hours

FMSHK Member Societies are offered 2 hours FREE rental exclusively.
(Applicable to societies who haven't used the rental service before)

Suitable for Meeting / Seminar / Press Conference / Personal Gathering

Well Equipped for Rental:
Sound system : microphones /
Notebook with LCD projector /
42" TV / Broadband Internet & wifi /
Refreshment Ordering, Drinks Ordering /
Printing & Photocopy Services

Multi Function Room I

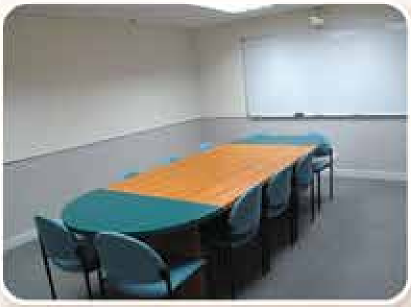

Lecture Hall

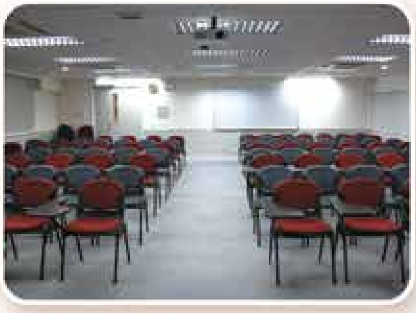

Council Chamber

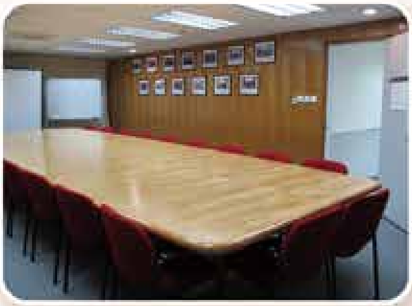

For enquiry and booking, please contact the Secretariat at 2527 8898.
http://www.fmshk.org/rental

Sunday	Monday	Tuesday	Wednesday	Thursday	Friday	Saturday
1	2	★ Zoom Live<br>HKMA-HKSH CME Programme 2021-2022 (Online)<br>Topic: Updates on Management of Type 2 Diabetes Mellitus<br>3	4	★ Zoom Live<br>Common Benign Perianal Diseases - Online<br>5	★ Zoom Live<br>Management of BPH in the Right Time - Online<br>6	7
8	9	★ Zoom Live<br>A New Way To Protect Older Adults Against Influenza - Recombinant Vaccine Technology - Online<br>10	★ The Hong Kong Neurosurgical Society Monthly Academic Meeting -To be confirmed<br>11	★ Zoom Live<br>HKMA-HKSTP CME Lecture - Diagnosis Of Common And Rare Neurological Diseases: Technological Updates And Clinical Applications (Online)<br>12	★ Zoom Live<br>Individualized Treatment in Osteoporosis - Beware of Increased Fracture Risk in Diabetes Patients - Online<br>13	14
15	★ Zoom Live<br>Prevention and risk reduction of CRC with the recent advancement of gut microbiome research - Online<br>16	★ Zoom Live<br>HKMA-GHK CME Programme 2021 - 2022 - Update in BPH Management (Online)<br>17	18	★ Zoom Live<br>Certificate Course for GPs 2022 - Updates on Management of COVID-19 - Online<br>19	★ Zoom Live<br>Updates on the Management of H. Pylori infections – Diagnosis and Treatment Approach - Online<br>20	21
22	23	24	★ Zoom Live<br>Updates of COVID-19 Vaccines in Youth and Children - Online<br>25	26	★ Zoom Live<br>Today's psoriasis management - Aim to clear - (Online)<br>27	28
29	★ Zoom Live<br>Recommendations on Prediabetes Management - Online<br>30	★ Zoom Live<br>Chronic Venous Disease: A Common Pain Point with Effective Treatment Strategies - Online<br>31				

Date / Time	Function	Enquiry / Remarks
**3 TUE** 2:00 PM	**Zoom Live**<br>**HKMA-HKSH CME Programme 2021-2022 (Online)**<br>**Topic: Updates on Management of Type 2 Diabetes Mellitus**<br>Organiser: Hong Kong Medical Association & Hong Kong Sanatorium & Hospital<br>Speaker: Dr YEUNG Chun-yip	HKMA CME Dept.<br>3108 2507<br>1 CME Point
**5 THU** 2:00 PM	**Zoom Live**<br>**Common Benign Perianal Diseases - Online**<br>Organiser: HKMA-KLN East Community Network<br>Speaker: Dr MAK Wing-chung, Tony	Mr Jeffrey CHEUNG<br>3108 2514<br>1 CME Point
**6 FRI** 2:00 PM	**Zoom Live**<br>**Management of BPH in the Right Time - Online**<br>Organiser: HKMA-KLN City Community Network<br>Speaker: Dr CHAN Chun-ki	Ms Candice TONG<br>3108 2513<br>1 CME Point
**10 TUE** 2:00 PM	**Zoom Live**<br>**A New Way To Protect Older Adults Against Influenza - Recombinant Vaccine Technology - Online**<br>Organiser: Hong Kong Medical Association<br>Speaker: Dr WONG King-ying	HKMA CME Dept.<br>3108 2513<br>1CME Point
**11 WED** 7:30 AM	**The Hong Kong Neurosurgical Society Monthly Academic Meeting -To be confirmed**<br>Organiser: Hong Kong Neurosurgical Society<br>Speaker: Dr CHEUNG Ling-kit	College of Surgeons of Hong Kong<br>Dr Calvin MAK<br>2595 6456<br>1.5 CME point
**12 THU** 2:00 PM	**Zoom Live**<br>**HKMA-HKSTP CME Lecture - Diagnosis Of Common And Rare Neurological Diseases: Technological Updates And Clinical Applications (Online)**<br>Organiser: Hong Kong Medical Association & Hong Kong Science Park<br>Speaker: Prof CHAN Ho-yin, Edwin	HKMA CME Dept<br>3108 2507<br>1CME Point
**13 FRI** 2:00 PM	**Zoom Live**<br>**Individualized Treatment in Osteoporosis - Beware of Increased Fracture Risk in Diabetes Patients - Online**<br>Organiser: HKMA-Shatin Community Network<br>Speaker: Dr Enoch WU	Ms Candice TONG<br>3108 2513<br>1CME Point
**16 MON** 2:00 PM	**Zoom Live**<br>**Prevention and Risk Reduction of CRC with the Recent Advancement of Gut Microbiome Research - Online**<br>Organiser: Hong Kong Medical Association<br>Speaker: Dr LAM Long-yan	HKMA CME Dept.<br>3108 2514<br>1CME Point
**17 TUE** 2:00 PM	**Zoom Live**<br>**HKMA-GHK CME Programme 2021 - 2022 - Update in BPH Management (Online)**<br>Organiser: Hong Kong Medical Association & Gleneagles Hong Kong Hospital<br>Speaker: Dr LAM-pei, Wayne	HKMA CME Dept<br>3108 2507<br>1CME Point
**19 THU** 2:00 PM	**Zoom Live**<br>**Certificate Course for GPs 2022 - Updates on Management of COVID-19 - Online**<br>Organiser: HKMA-KLN East Community Network, HA-United Christian Hospital &HK College of Family Physicians<br>Speaker: Dr Lily CHENG Shui-kuen	Ms Judy YU<br>3949 3043<br>1CME Point
**20 FRI** 2:00 PM	**Zoom Live**<br>**Updates on the Management of H. Pylori infections – Diagnosis and Treatment Approach - Online**<br>Organiser: Hong Kong Medical Association<br>Speaker: Dr LAM Long-yan, Kelvin	HKMA CME Dept.<br>3108 2507<br>1CME Point
**25 WED** 2:00 PM	**Zoom Live**<br>**Updates of COVID-19 Vaccines in Youth and Children - Online**<br>Organiser: Hong Kong Medical Association<br>Dr LUK Chi-kong, David	HKMA CME Dept.<br>2527 8452<br>1CME Point
**27 FRI** 2:00 PM	**Zoom Live**<br>**Today's psoriasis management - Aim to clear - (Online)**<br>Organiser: Hong Kong Medical Association<br>Speaker: Dr LAM Yuk-keung	HKMA CME Dept.<br>3108 2514<br>1CME Point
**30 MON** 2:00 PM	**Zoom Live**<br>**Recommendations on Prediabetes Management - Online**<br>Organiser: Hong Kong Medical Association<br>Dr AU YEUNG Yick-cheung	HKMA CME Dept.<br>3108 2507<br>1CME Point
**31 TUE** 2:00 PM	**Zoom Live**<br>**Chronic Venous Disease: A Common Pain Point with Effective Treatment Strategies - Online**<br>Organiser: HKMA-YTM Community Network<br>Speaker: Dr CHENG, Mina	Ms Candice TONG<br>3108 2513<br>1CME Point

Answers to Dermatology Quiz

Answers:

1. History of contact with any allergens or irritants should be elicited, such as necklaces, skincare products, perfumes, new scarfs, etc. On direct questioning, she admitted that she had been wearing a new necklace occasionally in the past few months.

2. The fundamental rule in diagnosing skin disease is to analyse the morphology and distribution of the skin lesions. In this patient, the morphology of the lesions was rather non-specific. The clue in diagnosis was the distribution, which surrounded the neck in a circular pattern and confined to the area of the necklace or scarf. The clinical impression is contact dermatitis.

3. Practically patch test is still the most important and useful test for contact dermatitis, even though it is only useful in allergic contact dermatitis. In irritant contact dermatitis, the diagnosis is mainly based on clinical grounds. In this patient, the patch test showed a strong reaction to nickel, which is still be most common allergen in causing allergic contact dermatitis in females.

Dr Lai-yin CHONG

MBBS(HK), FRCP(Lond, Edin, Glasg), FHKCP, FHKAM(Med)

Specialist in Dermatology & Venereology

The Federation of Medical Societies of Hong Kong
4/F Duke of Windsor Social Service Building, 15 Hennessy Road, Wanchai, HK
Tel: 2527 8898 Fax: 2865 0345

Hon. President
Dr Chok-wan CHAN 陳作耘醫生
Dr Dawson To-sang FONG 方道生醫生
Dr Raymond See-kit LO 勞思傑醫生
President
Prof Bernard Man-yung CHEUNG 張文勇教授
1st Vice-President
Dr Chun-kong NG 吳振江醫生
2nd Vice-President
Dr Ludwig Chun-hing TSOI 蔡振興醫生
Hon. Treasurer
Ms Tina Woan-tyng YAP 葉婉婷女士
Hon. Secretary
Dr Alson Wai-ming CHAN 陳偉明醫生
Executive Committee Members
Dr Jane Chun-kwong CHAN 陳真光醫生
Dr Kingsley Hau-ngai CHAN 陳厚毅醫生
Dr Kai-ming CHAN 陳啟明醫生
Dr Peggy Sau-kwan CHU 朱秀群醫生
Dr Samuel Ka-shun FUNG 馮加信醫生
Ms Ellen Wai-yin KU 顧慧賢小姐
Mr Benjamin Cheung-mei LEE 李祥美先生
Prof Eric Wai-choi TSE 謝偉財教授
Dr Haston Wai-ming LIU 廖偉明醫生
Dr Desmond Gia-hung NGUYEN 阮家興醫生
Dr Kwai-ming SIU 邵貴明醫生
Dr Tony Ngan-fat TO 杜銀發醫生
Mr William Kai-hung TSUI 徐啟雄先生
Dr Victor Hip-wo YEUNG 楊協和醫生
Dr Edwin Chau-leung YU 余秋良醫生
Ms Manbo Bo-lin MAN (Co-opted) 文保蓮女士
Dr Wilfred Hing-sang WONG (Co-opted) 黃慶生博士

Founder Members

British Medical Association (Hong Kong Branch)
英國醫學會(香港分會)

President
Dr Raymond See-kit LO 勞思傑醫生
Vice-President
Dr Adrian WU 鄔揚源醫生
Hon. Secretary
Dr Terry Che-wai HUNG 洪致偉醫生
Hon. Treasurer
Dr Jason BROCKWELL
Council Representatives
Dr Raymond See-kit LO 勞思傑醫生
Dr Tse-ming CHEUNG 張子明醫生
Tel: 2527 8898 Fax: 2865 0345

The Hong Kong Medical Association
香港醫學會

President
Dr CHOI Kin 蔡 堅醫生
Vice- Presidents
Dr Chi-man CHENG 鄭志文醫生
Dr Siu-king MAK 麥肇敬醫生
Hon. Treasurer
Dr Victor Hip-wo YEUNG 楊協和醫生
Hon. Secretary
Dr James Tak-kwan FUNG 馮德焜醫生
Council Representatives
Dr Victor Hip-wo YEUNG 楊協和醫生
Chief Executive
Ms Jovi LAM 林偉珊女士
Tel: 2527 8285 (General Office)
2527 8324 / 2536 9388 (Club House in Wanchai / Central)
Fax: 2865 0943 (Wanchai), 2536 9398 (Central)
Email: hkma@hkma.org Website: http://www.hkma.org

The HKFMS Foundation Limited 香港醫學組織聯會基金

Board of Directors

President
Prof Bernard Man-yung CHEUNG 張文勇教授
1st Vice-President
Dr Chun-kong NG 吳振江醫生
2nd Vice-President
Dr Ludwig Chun-hing TSOI 蔡振興醫生
Hon. Treasurer
Ms Tina Woan-tyng YAP 葉婉婷女士
Hon. Secretary
Dr Alson Wai-ming CHAN 陳偉明醫生
Directors
Mr Samuel Yan-chi CHAN 陳恩賜先生
Dr Samuel Ka-shun FUNG 馮加信醫生
Ms Ellen Wai-yin KU 顧慧賢女士
Dr Raymond See-kit LO 勞思傑醫生
Dr Aaron Chak-man YU 余則文醫生

SmofKabiven®

THE MIX FOR LIFE

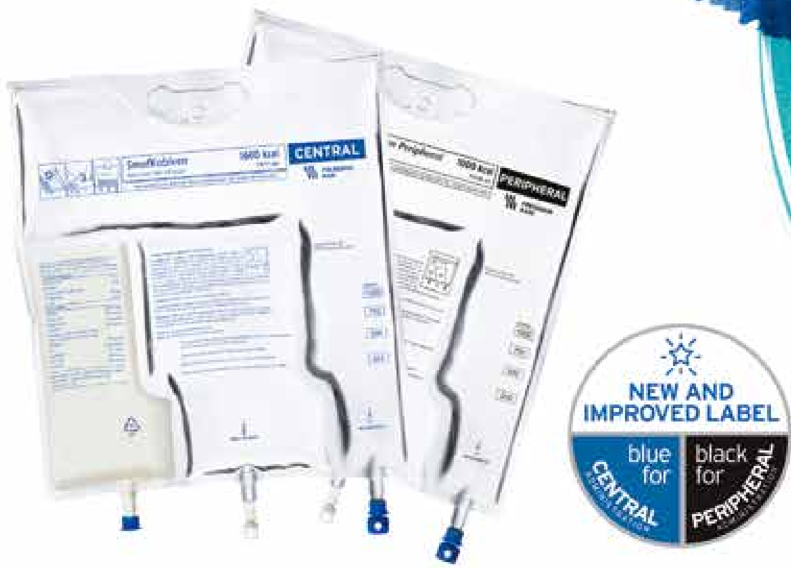

ω-3 enriched PN – proven to improve clinical outcomes with excellent safety profile[1]:

- Significantly reduced length of hospital stay overall by **3 days**.
- Significantly reduced infection rate by **39%**

- Available in different bag sizes (Central: 493/986/1477/1970 ml, Peripheral: 1206/1448/1904 ml)
- Extensive compatibility data with micronutrients

Complete parenteral nutrition therapy with micronutrients

- All PN prescriptions should include a daily dose of multi-vitamins and trace elements[2-3]
- After surgery, in those patients who are unable to be fed via the enteral route, and in whom total or near total parenteral nutrition is required, a full range of vitamins and trace elements should be supplemented on a daily basis[3]

Approved for children ≥ 2 years

SmofKabiven® contains unique SMOFlipid®

SMOFlipid® – A 4-oil mix with a well-balanced fatty acid pattern containing purified natural fish oil

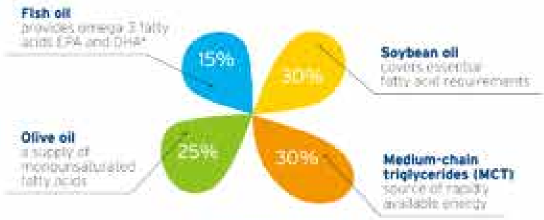

+ additional vitamin E
(approx. 200 mg α-tocopherol/liter) to counteract lipid peroxidation and oxidative stress[4]

*EPA: eicosapentaenoic acid
DHA: docosahexaenoic acid

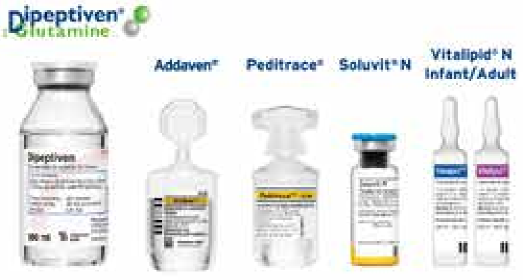

References :
1. L. Pradelli et al. Clinical Nutrition 33 (2014) 785-792
2. Singer et al. (2009) ESPEN Guidelines on parenteral nutrition: Intensive Care. Clinical Nutrition 28: 387-400
3. Braga et al. (2009) ESPEN Guidelines on Parenteral Nutrition: Surgery. Clinical Nutrition, 28: 378-386
4. [illegible] Gastroenterology 2002;123(5):1[illegible] http://www.espen.org/[illegible]

FRESENIUS KABI
caring for life

Fresenius Kabi Hong Kong Ltd.
Room 5001-5027, 50/F, Sun Hung Kai Centre,
30 Harbour Road, Wanchai, Hong Kong
Tel : (852) 2152 1330   Fax : (852) 2119 0815
www.fresenius-kabi.com
